# Global heterogeneity in folic acid fortification policies and implications for prevention of neural tube defects and stroke: a systematic review

**DOI:** 10.1016/j.eclinm.2023.102366

**Published:** 2023-12-12

**Authors:** Matthew Quinn, Jim Halsey, Paul Sherliker, Hongchao Pan, Zhengming Chen, Derrick A. Bennett, Robert Clarke

**Affiliations:** a Clinical Trial Service Unit and Epidemiological Studies Unit, Nuffield Department of Population Health, University of Oxford, Oxford, United Kingdom; b Medical Research Council Population Health Research Unit of University of Oxford, United Kingdom

**Keywords:** Folic acid fortification, Plasma folate, Neural tube defects, Stroke

## Abstract

**Background:**

Folic acid (pteroylmonoglutamic acid) supplements are highly effective for prevention of neural tube defects (NTD) prompting implementation of mandatory or voluntary folic acid fortification for prevention of NTDs. We used plasma folate levels in population studies by country and year to compare effects of folic acid fortification types (mandatory or voluntary folic acid fortification policies) on plasma folate levels, NTD prevalence and stroke mortality rates.

**Methods:**

We conducted systematic reviews of (i) implementation of folic acid fortification in 193 countries that were member states of the World Health Organization by country and year, and (ii) estimated population mean plasma folate levels by year and type of folic acid fortification. We identified relevant English language reports published between Jan 1, 1990 and July 31, 2023 using Google Scholar, Medline, Embase and Global Health. Eligibility criteria were observational or interventional studies with >1000 participants. Studies of pregnant women or children <15 years were excluded. Using an ecological study design, we examined the associations of folic acid fortification types with NTD prevalence (n = 108 studies) and stroke mortality rates (n = 3 countries).

**Findings:**

Among 193 countries examined up to 31 July 2023, 69 implemented mandatory folic acid fortification, 47 had voluntary fortification, but 77 had no fortification (accounting for 32%, 53% and 15% of worldwide population, respectively). Mean plasma folate levels were 36, 21 and 17 nmol/L in populations with mandatory, voluntary and no fortification, respectively (and proportions with mean folate levels >25 nmol/L were 100%, 15% and 7%, respectively). Among 75 countries with NTD prevalence, mean (95% CI) prevalence per 10,000 population were 4.19 (4.11–4.28), 7.61 (7.47–7.75) and 9.66 (9.52–9.81) with mandatory, voluntary and no folic acid fortification, respectively. However, age-standardised trends in stroke mortality rates were unaltered by the introduction of folic acid fortification.

**Interpretation:**

There is substantial heterogeneity in folic acid fortification policies worldwide where folic acid fortification are associated with 50–100% higher population mean plasma folate levels and 25–50% lower NTD prevalence compared with no fortification. Many thousand NTD pregnancies could be prevented yearly if all countries implemented mandatory folic acid fortification. Further trials of folic acid for stroke prevention are required in countries without effective folic acid fortification policies.

**Funding:**

10.13039/501100000265Medical Research Council (UK) and 10.13039/501100000274British Heart Foundation.

**Research in context**

**Evidence before this study**

Folic acid supplements are highly effective for the prevention of neural tube defects (NTDs) if administered before conception and during the first trimester of pregnancy. Consequently, many countries worldwide have implemented either mandatory or voluntary folic acid fortification for prevention of neural tube defects. Observational studies have demonstrated that the risks of NTDs are linearly and inversely associated with maternal red blood cell folate levels (when plotted on logarithmic scales). However, we used plasma folate levels as a surrogate measure of red blood cell folate levels to assess effects of different folic acid fortification types on plasma folate levels, NTD prevalence and stroke mortality rates. We conducted an ecological study of population mean plasma folate levels (n = 108 studies), NTD prevalence (n = 108 countries) and age-standardised stroke mortality rates in three selected countries with different folic acid fortification types.

**Added value of this study**

By 31 July 2023, 69 countries implemented mandatory folic acid fortification, 47 had voluntary fortification, but 77 had no fortification. The proportions having plasma folate levels >25 nmol/L for prevention of NTD were 100%, 15% and 7%, among those with mandatory, voluntary or no fortification, respectively. Mean (95% CI) NTD prevalence rates per 10,000 were 4.19 (4.11–4.28), 7.61 (7.47–7.75) and 9.66 (9.52–9.81) in populations with mandatory, voluntary and no fortification, respectively, but there were no differences in age-standardised stroke mortality rates between the different fortification types.

**Implications of all the available evidence**

There is substantial heterogeneity in folic acid fortification types worldwide resulting in differences of 50–100% higher mean plasma folate levels and 25–50% lower NTD prevalence by different folic acid fortification types (but no differences in stroke mortality rates). The results suggest that many thousand NTD pregnancies could be prevented yearly if all countries implemented mandatory folic acid fortification. Further trials of folic acid for stroke prevention are required in countries that have not yet implemented effective folic acid fortification policies.

## Introduction

Randomised trials have demonstrated that folic acid (pteroylmonoglutamic acid) supplements taken prior to conception and during the first 12 weeks of pregnancy reduced the incidence of neural tube defects (NTDs) by almost 80%.^1^, ^2^, ^3^ Hence, all women of reproductive age are recommended to take 400 μg of folic acid, but since most pregnancies are unplanned, many countries have implemented folic acid fortification of staple foods for the prevention of NTDs.^4^^,^^5^

Mandatory folic acid fortification was first implemented in the United States (USA) (140 μg of folic acid per 100 g of cereal grains) and Canada (150 μg of folic acid per 100 g of cereal grains) in 1998 and in many countries elsewhere in subsequent years.^6^ In contrast, voluntary folic acid fortification was implemented in the United Kingdom, elsewhere in Europe and other regions of the world, but these have resulted in more variable population mean plasma folate levels between and within populations than mandatory fortification.^7^, ^8^, ^9^ However, while previous studies highlighted variable effects of different folic acid fortification types on plasma folate levels in individual countries,^10^^,^^11^ there have been no systematic reviews of the worldwide evidence of folic acid fortification types on plasma folate levels.

Mandatory folic acid fortification may have benefits that extend beyond NTD prevention. Folic acid also lowers plasma homocysteine levels that have been linked with risk of stroke and ischaemic heart disease (IHD),^12^ but it is unclear if these associations are causal. Randomised trials of folic acid conducted in Western populations reported no beneficial effects of folic acid supplements on stroke or IHD, but these trials were conducted after implementation of folic acid fortification.^12^ In contrast, the Chinese Stroke Primary Prevention trial (CSPPT2), was conducted in a population without effective folic acid fortification reported that folic acid reduced the incidence of stroke by 20% (but had no effect on CHD).^13^ Mendelian randomisation (MR) studies of methylene-tetrahydrofolate reductase (*MTHFR C677T*) variant (that is associated with 25% higher plasma homocysteine levels) demonstrated that individuals with TT vs CC genotypes for *MTHFR* had higher stroke incidence in Chinese, but not in Western populations.^14^^,^^15^ The discrepant results of randomised trials of folic acid for stroke prevention and MR studies of *MTHFR* and stroke may reflect the effects of different folic acid fortification types on plasma folate levels.^15^

Maternal red blood cell folate levels are the recommended biomarker for population folate status to assess effects of different folic acid fortification policies.^16^ Population studies conducted in Europe and in China reported that risks of NTDs were linearly and inversely associated with maternal red-blood cell folate levels (when each were plotted on logarithmic scales), and demonstrate that a doubling in red-blood cell folate levels was associated with a halving in NTD risk.^17^^,^^18^ Unfortunately, access to red-cell folate assays is limited to a few specialist research laboratories worldwide. In contrast, plasma folate levels are widely used in clinical practice and research studies to assess population folate status in diverse populations. Little is known about the effects of different folic acid fortification types on plasma folate levels, NTD prevalence and stroke mortality.^19^, ^20^, ^21^, ^22^, ^23^, ^24^ Moreover, the quality of folate assays has varied over several decades, which may underestimate differences between populations. However, microbiological plasma folate assays that are standardised for assay differences have no such biases, and hence, can be more reliably compared between populations over different calendar periods.^25^

We conducted an ecological study to assess the associations of population mean plasma folate levels with risk of neural tube defects and age-standardised stroke mortality by folic acid fortification types. The aims of the study were (i) to assess population mean plasma folate levels in healthy adults before and after implemention of different folic acid fortification policies between 1990 and 2023; (ii) examine associations of country-specific differences in previously reported NTD prevalence by different folic acid fortification policies^26^; and (iii) examine trends in age-standardised stroke mortality by different fortification policies in selected countries with reliable evidence on plasma folate levels recorded over several decades.

## Methods

### Review of folic acid fortification policies

We searched published studies on national folic acid fortification policies by country and calendar year to clarify details of implementation of mandatory or voluntary folic acid fortification policies by 24 July 2023 in 193 countries that were member states of the World Health Organisation (WHO).^27^ Google scholar was searched using the terms: “national policy or legislation”, “folic acid”, “fortification” and “country name”. Mandatory fortification was defined as a policy of the compulsory addition of folic acid at a specified standard to staple food products, chiefly wheat flour, maize or rice. Voluntary fortification was defined as the presence of national guidance on the addition of folic acid to food products by food manufacturers. The dates for introduction of folic acid fortification refer to dates of legislation mandating implementation of fortification. No fortification was defined as the absence of legislation for either a voluntary or mandatory fortification policy. Published reports were screened to extract details of folic acid fortification policies and were corroborated by national government websites to confirm details of folic acid fortification policies (Table S1, Appendix p17–22).^28^^,^^29^

### Data extraction of folic acid fortification policies

Countries were classified using the World Bank income categories (high income, upper middle income, lower middle income and low income countries).^30^ Data extraction included country, income status, food vehicle, legislation on folic acid and fortification, nutrient level in standard (recommended level of folic acid fortification), nutrient level standard (upper and lower limits of folic acid per kg), year of legislation and legislation source. Changes in folic acid fortification policies and changes in upper and lower limits for nutrient levels for the addition of folic acid to food products were extracted. Individual countries could have multiple folic acid fortification policies for different products. In such situations, the available folic acid fortification standards for wheat flour were presented in the analyses. Several countries with voluntary fortification did not specify standards for folic acid fortification and the level of folic acid defined as a “significant amount” in a product was used as a proxy for nutrient levels in standards in such countries.

### Systematic review of plasma folate levels

**Fig. 1:**
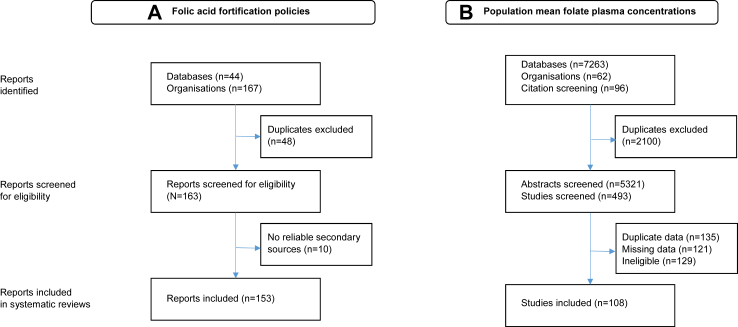
**Flow diagram of included studies on folic acid fortification policies and population mean plasma folate concentrations**. The figure shows the numbers of included studies in systematic reviews of (A) worldwide folic acid fortification policies and (B) worldwide population mean plasma folate concentrations by calendar year and country.

Population studies of plasma folate levels were obtained by searching Medline, Embase and Global Health databases (Fig. 1) using pre-specified keywords initially in August 2021 and updated until July 31, 2023 for publications (full-text articles or conference abstracts) published between January 1, 1990 and July 31, 2023. The study was exempt from institutional ethics approval as only publicly available data were used. A protocol for the systematic review of population mean plasma folate levels by folic acid fortification policies and their associations with NTDs and stroke mortality is included in the Supplementary Methods.

The systematic review followed Preferred Reporting Items of Systematic reviews and Meta-Analyses reporting guidelines.^31^ National food surveys, cross-sectional studies, and control groups of randomised trials and case–control studies were included. Studies were restricted to adults aged 15 years or older, and excluded studies of pregnant women (because of pregnancy-related variation in plasma folate levels and their high use of folic acid supplements).^32^^,^^33^ Studies involving <1000 participants were excluded, but exceptions were made for studies near to the limit if a country would be otherwise unrepresented (such as Ethiopia and Pakistan).^34^^,^^35^ Non-English language publications were excluded unless the data had already been extracted by the WHO Vitamin and Mineral Nutrition Information System (VMNIS), a database of population micronutrient levels from published reports or government databases.^36^ This allowed the inclusion of data for studies conducted in low- and middle-income countries, including Costa Rica, Ecuador, Cote d’Ivoire and Mexico.^37^, ^38^, ^39^, ^40^, ^41^ Serum or plasma folate levels were used interchangeably.^33^ References were managed using Covidence.^42^ The report titles and abstracts were screened, full text reports obtained and reviewed against pre-specified inclusion and exclusion criteria and data from included reports extracted by a single reviewer (MQ) (Table S2, Appendix p23–29). Data extraction and summary results were checked and confirmed by a 2nd reviewer (RC).

### Statistical analyses

Data on serum or plasma folate levels were extracted (or where necessary converted) into SI units (nmol/L) (Table S2, Appendix p23–29). If identical data were presented in several studies, the study with data on the maximum number studied was included. If information on calendar period of data collection was unavailable, the year of publication was used instead. If no lower age limits were reported, data were assumed to be for adults aged 15 years or older. Mean (or median) plasma folate values and standard deviations (SD) were extracted where possible. If plasma folate levels were presented as medians or geometric means, these were converted to arithmetic means assuming a normal distribution.^43^ Where SDs were unavailable these were imputed using mean standard errors, 95% CIs or interquartile ranges or other measures of spread provided by the authors. Population mean folate levels were assumed to have a normal distribution. Grouped arithmetic means (weighted by study size) and standard deviations were estimated by country, decade of study (1990–1999, 2000–2009, 2010–2023), region, income group and fortification type. Data on national populations were obtained from World Health Statistics 2022.^44^ Sensitivity analyses were restricted to analyses of plasma folate levels measured using a microbiological assay that were independent of temporal changes in plasma folate assay reagents. To evaluate the quality of included studies, the analyses of folate levels were provided for any plasma folate assay or a microbiological plasma folate assay.

### NTD prevalence rates

Data on country-specific NTD prevalence by calendar year were obtained from a previously reported country-specific systematic review of NTD prevalence by calendar year (Table S3, Appendix p30–34).^26^ All studies were reclassified by folic acid fortification types by country and calendar year. Studies with missing 95% CI were excluded. The weighted mean (95% CI) values of NTD prevalence rates were obtained by conducting a meta-analysis of country-specific prevalence and their 95% CI using the double arcsine transformation to stabilise variance by folic acid fortification categories using a previously described method.^45^

### Trends in stroke mortality rates

Annual age standardised stroke mortality rates by sex and calendar year for USA, UK and China among adults aged 40–79 years were extracted from World Health Organisation mortality databases and United Nations population tables. Countries were representative of different folic acid fortification policies and also had extensive data on plasma folate levels over several decades. Mortality rates were estimated as the mean of the annual rates in seven component 5-year age groups, and standardised for age by averaging rates across the seven age groups.

### Ethics

No specific ethical approval was sought for this study, as it involved summary data from publicly available data from government sources from previous publications.

### Role of funding source

The funders had no role in the study design, data collection, analysis, data interpretation, writing of any reports, or decisions to submit any paper for publication.

## Results

### A review of implementation of folic acid fortification policies by country and year

**Fig. 2:**
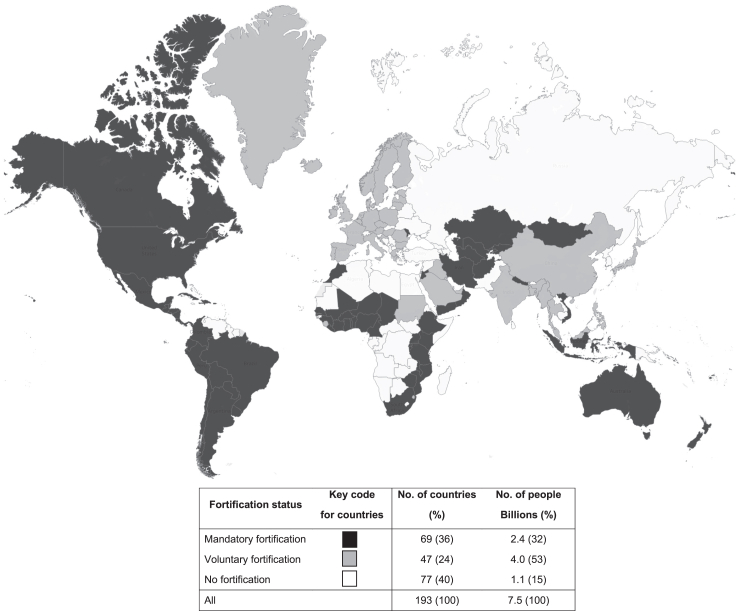
**Distribution of folic acid fortification types by country**. Countries with mandatory folic acid fortification, voluntary folic acid fortification or no folic acid fortification are shown in black, grey and white colours, respectively.

A review of national legislation on folic acid fortification enacted up to 31 July 2023 identified 163 reports (Fig. 1) of which 153 were confirmed by national legislation (or other statutory guidance) on folic acid fortification. Fig. 2 shows a map of the world, indicating that most of North America, South America, Australia and New Zealand and several countries in Africa had implemented mandatory fortification before July 2023. In contrast, most of Europe, China and parts of Africa had implemented voluntary folic acid fortification by July 2023. However, most countries in Eastern Europe or Africa have not yet implemented any folic acid fortification.

Figure S1 and Table S1 show the types of folic acid fortification and doses of folic acid used in standard food vehicles, by country, calendar year and folic acid fortification types (Appendix p17–22). Among 193 countries examined in July 2023, 69 implemented mandatory folic acid fortification, 47 had voluntary folic acid fortification, but 77 countries had no folic acid fortification. Details of the year of implementation of folic acid fortification by country are provided in Figure S2 (Appendix p15). The nutrient level of folic acid in standard food vehicles was higher in populations with mandatory folic acid fortification than those with voluntary folic acid fortification.

**Fig. 3:**
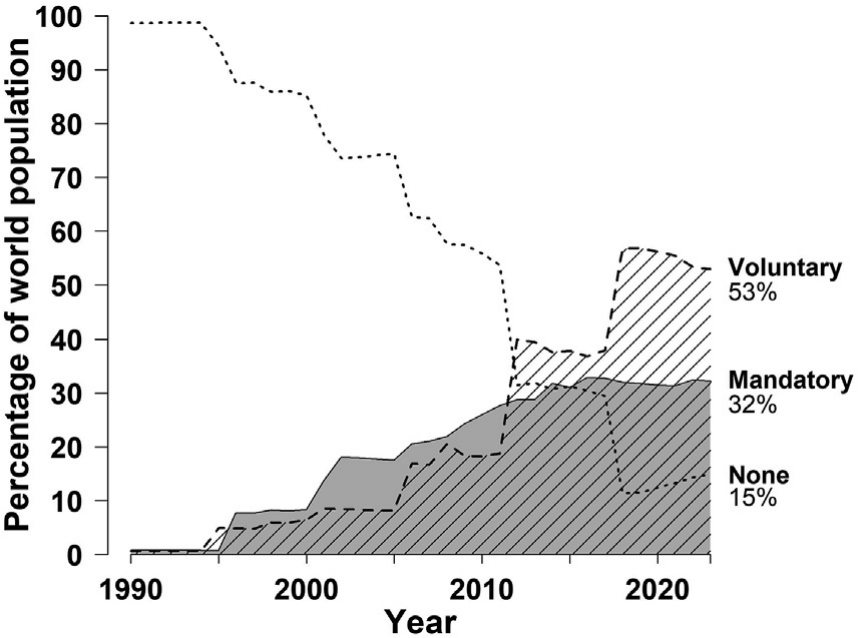
**Cumulative distribution of folic acid fortification types by calendar year**. Percentage of the worldwide populations with mandatory folic acid fortification, voluntary folic acid fortification or no folic acid fortification are shown in dark, shaded or no colours, respectively.

Fig. 3 shows a cumulative distribution of implementation of voluntary or mandatory folic acid fortification in the worldwide population by calendar year and demonstrates a progressive increase in the implementation of mandatory folic acid fortification from 0% in 1995 to 31% in 2023. Among the worldwide population by July 2023, 2.4 billion (32%) had mandatory fortification, 4.0 billion people (53%) had voluntary folic acid fortification, but 1.13 billion (15%) had no folic acid fortification (Table 1).

**Table 1:** National legislation on folic acid fortification by calendar year, geographic region and income level of country.

	None		Voluntary fortification			Voluntary fortification		
	No. of countries	No. of people, millions (%)	No. of countries	No. of people, millions (%)	Median (range) nutrient level standard	No. of countries	No. of people, millions (%)	Median (range) nutrient level standard
**Geographic region**								
Europe	18	331.0 (39.2)	28	509.8 (60.3)	0.30 (0.30–0.30)	1	4.1 (0.5)	1.40 (1.40–1.40)
North America	9	6.0 (1.2)				3	490.4 (98.8)	1.54 (1.50–2.00)
Central & South America	4	29.8 (5.9)	1	32.0 (6.3)	1.20 (1.20–1.20)	18	443.4 (87.8)	1.80 (0.40–3.00)
Africa	20	244.0 (22.1)	3	50.6 (4.6)	1.50 (1.30–2.08)	25	811.0 (73.4)	2.30 (1.30–5.00)
Middle East	7	367.7 (55.9)	5	88.7 (13.5)	1.75 (1.75–2.10)	8	200.8 (30.6)	1.50 (1.00–1.53)
Oceania	9	9.2 (5.2)	2	138.2 (77.2)	1.92 (1.92–1.92)	6	31.5 (17.6)	2.00 (2.00–2.50)
Asia	10	142.9 (3.8)	8	3206.2 (84.2)	1.30 (0.10–2.00)	8	457.4 (12.0)	1.45 (1.00–5.11)
**Income level of country**								
High income	17	79.4 (6.7)	33	675.7 (57.2)	0.30 (0.30–1.75)	9	426.6 (36.1)	1.80 (1.50–2.50)
High middle income	30	328.2 (11.4)	6	1614.1 (56.0)	1.92 (0.30–2.10)	19	940.8 (32.6)	1.80 (0.40–3.00)
Low middle income	20	525.2 (18.1)	6	1717.3 (59.0)	1.30 (0.10–2.00)	24	667.2 (22.9)	1.80 (1.00–5.11)
Low income	10	197.9 (31.9)	2	18.3 (3.0)	1.19 (0.30–2.08)	17	404.0 (65.1)	2.22 (1.00–2.60)
**Calendar year**								
1995	189	3851.9 (94.4)	3	265.4 (4.9)	1.26 (0.60–1.92)	1	33.7 (0.6)	1.75
2000	181	3749.0 (85.3)	5	373.2 (6.5)	1.92 (0.60–2.10)	7	476.1 (8.3)	1.54 (1.50–1.80)
2005	169	3526.8 (74.5)	7	499.9 (8.1)	1.96 (0.60–2.10)	17	1070.1 (17.4)	1.54 (0.40–3.00)
2010	113	2890.4 (55.9)	38	1193.7 (18.2)	0.30 (0.30–2.10)	42	1694.1 (25.9)	1.80 (0.40–3.00)
2015	89	1849.3 (31.2)	46	2634.6 (37.8)	0.30 (0.30–2.10)	58	2154.5 (30.9)	1.80 (0.40–5.00)
2020	78	967.1 (12.4)	49	4139.4 (56.2)	0.30 (0.10–2.10)	66	2309.1 (31.4)	1.80 (0.40–5.11)
**All**^a^	77	1130.7 (14.9)	47	4025.5 (53.0)	0.30 (0.10–2.10)	69	2438.6 (32.1)	1.80 (0.40–5.11)

a Data up to and including July 2023.

### Systematic review of plasma folate levels in population studies

A total of 5321 abstracts were screened to identify 493 reports and data were extracted from 108 population studies of mean plasma folate levels of healthy adults (Fig. 1). Table 2 shows the mean plasma (or serum) folate levels measured by any folate assay. Mean plasma folate levels increased in successive decades and were highest in North and South America, intermediate for Europe and lowest for China.

**Table 2:** Population mean plasma folate concentrations by folic acid fortification type, geographic region and calendar year.

	Plasma folate (by any assay)			Plasma folate (by microbiological assay)		
	No. of studies	No. of people	Mean (SD) nmol/L	No. of studies	No. of people	Mean (SD) nmol/L
**Type of fortification**						
None	67	177,616	16.5 (8.1)	22	59,648	14.0 (7.4)
Voluntary	18	45,369	20.5 (7.3)	5	12,632	24.9 (10.6)
Mandatory	26	94,688	35.6 (11.5)	7	10,012	46.0 (16.5)
**Year of initiation**						
<2000	31	106,027	17.9 (10.8)	8	33,324	12.1 (7.5)
2000–2009	51	113,418	24.4 (15.1)	20	40,773	26.1 (16.4)
2010–2022	28	97,356	26.3 (11.5)	6	8195	17.2 (13.9)
**Geographic region Europe**						
2006 or earlier	24	79,227	15.2 (6.5)	10	36,498	13.4 (8.5)
Post-2006	10	28,778	21.4 (9.0)	5	13,567	24.0 (10.5)
**America/Australia**						
1996 or earlier	8	27,051	18.2 (7.6)			
Post-1996	25	101,131	33.9 (13.0)	6	9159	45.6 (17.8)
**China**						
2012 or earlier	15	27,274	13.2 (7.1)	7	14,262	12.3 (6.8)
Post-2012	5	19,391	20.1 (3.2)			
**All**	108	317,673	22.8 (12.9)	34	82,292	19.5 (14.6)

Plasma folate contentrations are presented for any folate assays and subset measured by a microbiological folate assay.

Means are weighted by study size.

SDs were SD of means over all studies but were unweighted. America includes North, Central & South America.

Total no. studies by fortification status exceeds 108 as 3 studies had components with both voluntary & mandatory fortification. Total no. studies by year of initiation exceeds 108 as 2 studies had components starting in different time period categories.

**Fig. 4:**
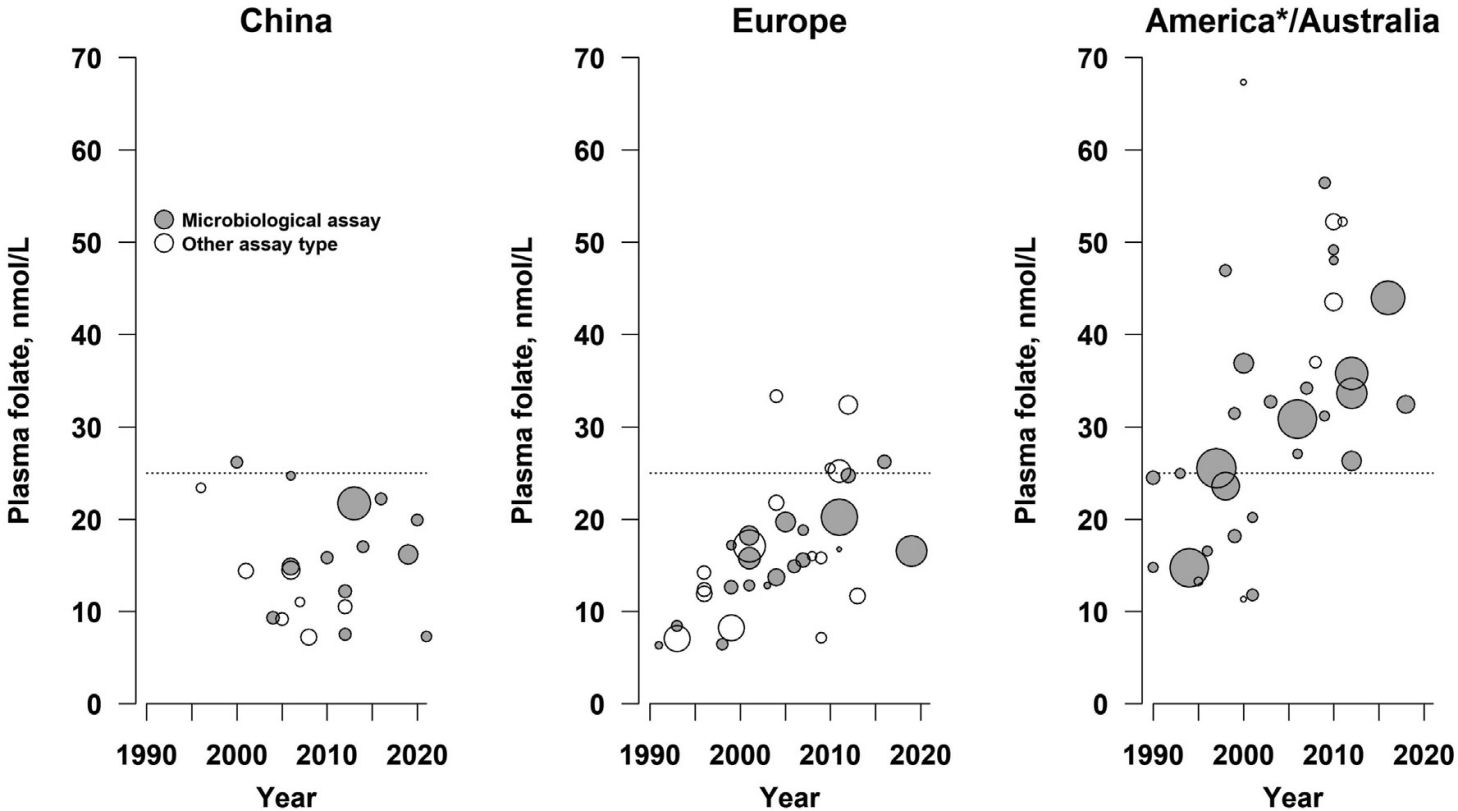
**Plasma folate levels by calendar year in population studies in China, Europe and America/Australia**. Plasma folate levels that were measured using microbiological assays are shown in dark circles and measured by other assays are shown in open circles, respectively. The size of the circles is proportional to the number of individuals studied. The horizontal dashed line is plasma folate of 25 nmol/L associated with substantial reduction in NTD risk. ∗America refers to North, Central and South America.

Fig. 4 shows the mean folate levels for individual studies weighted by the number of people in each study by calendar year for the Americas (North, Central and South America) and Australia, Europe and China, as being representative of mandatory, voluntary or no effective folic acid fortification, respectively. The USA and Canada were among the first countries to introduce mandatory folic acid fortification in 1996 and 1998, respectively, followed by countries in Central and South America in the late 1990s and 2000s, and Australia in 2009. Data were available from 33 studies conducted in America and Australia involving 128,182 participants (Table 2).The mean plasma folate concentrations were 18.2 nmol/L prior to fortification and 33.9 nmol/L after fortification using any assay. Among a subset of 6 US studies, mean folate status that were assessed after introduction of mandatory fortification using a microbiological assay were 45.6 nmol/L.

The European Union, including the UK, introduced standards for voluntary fortification in 2006. Data from Europe (including the UK) were available on 34 studies involving 108,005 participants. The mean plasma folate concentrations in Europe increased from 15.2 to 21.4 nmol/L using any folate assay after the introduction of voluntary fortification. Among a subset of European studies in which plasma folate levels were assessed by microbiological assay, mean plasma folate levels increased from 13.4 to 24.0 nmol/L, respectively.

China introduced legislation to permit voluntary folic acid fortification in 2012, but this has not been fully implemented. Data were available on 20 studies conducted in China involving 46,665 participants showed that the mean plasma folate concentrations in Chinese adults increased modestly from 13.2 nmol/L prior to fortification and 20.1 nmol/L after fortification. Among a subset of Chinese studies involving plasma microbiological assay, the mean folate levels in China were only 12.3 nmol/L. Importantly, mean plasma folate levels >25 nmol/L associated with a substantial reduction in NTD risk were achieved in 100% of American and Australian population studies with mandatory fortification, 15% of European studies with voluntary fortification and 7% of Chinese studies with no effective folic acid fortification.

### Mean plasma folate levels, by assay and fortification types

**Fig. 5:**
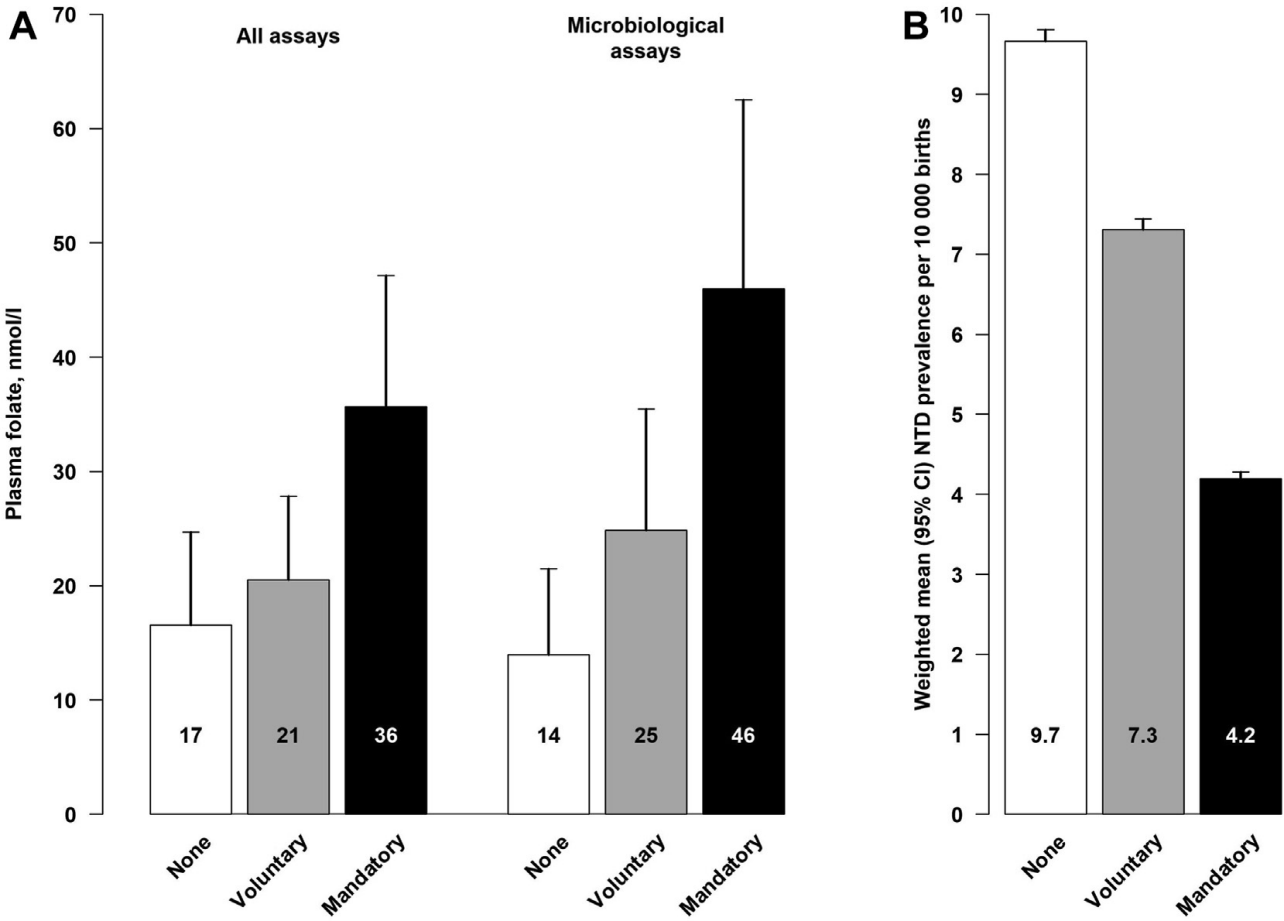
**Population mean (SD) plasma folate levels (A) and prevalence of neural tube defects (B), by folic acid fortification types**. (A) shows mean plasma folate levels for all assays and for the subset with microbiological assays. The results for populations with mandatory, voluntary and no fortification shown in black, grey and white colours, respectively. The error bars in panel A are standard deviations of the mean folate levels. (B) shows the weighted mean (95% CI) prevalence rates of NTDs for countries with mandatory, voluntary or no folic acid fortification, respectively.The error bars in panel B are 95% confidence intervals.

Fig. 5 (A) shows the mean (SD) plasma folate levels for any assay and the subset determined by microbiological assays. The results show substantial differences in mean plasma folate levels when assessed by any folate assay (35.6 vs 20.5 vs 16.5 nmol/L for populations with mandatory, voluntary or no folic acid fortification, respectively). Plasma folate levels measured using the more reliable microbiological assay had more extreme differences by fortification policies (46.0 vs 24.9 vs 14.0 nmol/L for populations with mandatory, voluntary fortification or no folic acid fortification, respectively).

### Differences in NTD prevalence by folic acid fortification types

The systematic review of NTD prevalence included 108 studies from 75 different countries (Table S3, Appendix p30–34) and these country-specific NTD prevalences were re-classified by calendar year and folic acid fortification types (26 mandatory, 33 voluntary and 14 with no fortification). Fig. 5 (B) shows that the mean (95% CI) prevalence of NTDs per 10,000 births were 4.19 (4.11–4.28), 7.61 (7.47–7.75) and 9.66 (9.52–9.81) in populations with mandatory, voluntary and no folic acid fortification, respectively. Thus, mandatory folic acid fortification was associated with about 50% lower prevalence of NTDs, but voluntary folic acid fortification was only associated with about 20% lower NTD prevalence compared with populations with no fortification.

### Trends in age-standardised stroke mortality rates by calendar year in USA, UK and China

**Fig. 6:**
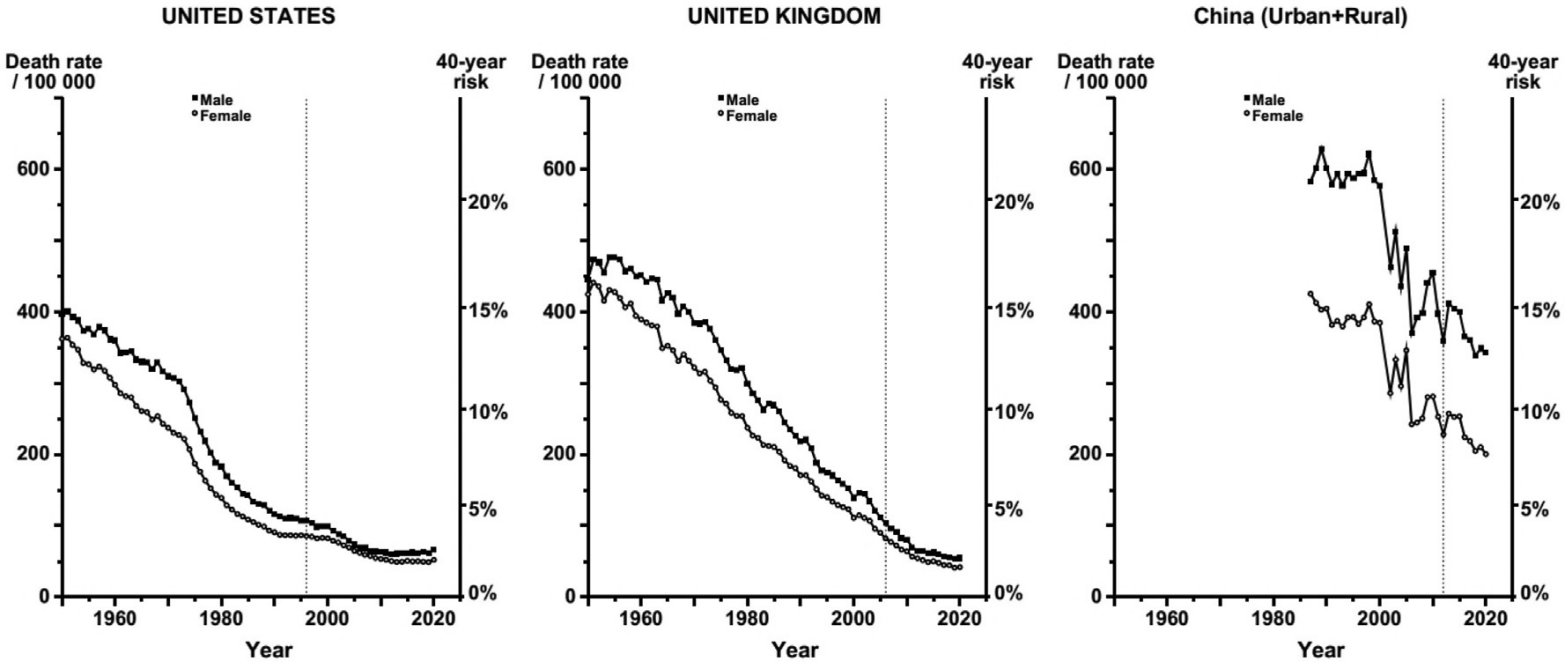
**Age-standardised stroke death rates at ages 40–79 years in United States, United Kingdom and China, by sex:** Data are shown for 1950–2020 in both the United States and United Kingdom and from 1990 to 2020 in China. The vertical dotted lines are the calendar years of introduction of mandatory folic acid fortification in United States and voluntary fortification in the United Kingdom and China, respectively.

Fig. 6 compares annual age-standardised mortality rates from stroke by sex and calendar year for USA, UK and China among adults aged 40–79 years. In USA, stroke death rates per 100,000 declined prior to the introduction of folic acid fortification from 117, 98, 63 and 67 in 1990, 2000, 2010, and 2020 in males and from 91, 83, 54 and 52 in females in the corresponding time periods. In the UK, stroke death rates per 100,000 also declined in 1990, 2000, 2010 and 2020 from 219, 139, 81 and 54 in males and from 171, 111, 64, to 42 in females, respectively. In China, stroke death rates per 100,000 in males also declined from 601, 577, 454, to 343 in 1990, 2000, 2010 and 2020 and from 404, 385, 281 and 200 in females, respectively. Overall, the age-standardised stroke mortality rates were higher in men than in women in all populations and stroke mortality rates remained 3 to 4-fold higher in China than in the USA, but were about 30–50% higher in the USA than in the UK.

## Discussion

There is substantial heterogeneity in population mean plasma folate levels and NTD prevalence rates between countries reflecting variable implementation of different folic acid fortification policies over the last three decades. By July 2023, 69 countries had implemented mandatory folic acid fortification, 47 had voluntary folic acid fortification, but 77 countries still did not implement any folic acid fortification. Populations with mandatory folic acid fortification had 3 to 4-fold higher mean plasma folate levels than those without any folic acid fortification. In contrast, voluntary folic acid fortification was associated with 2-fold higher mean plasma folate levels than those without any fortification. Mean plasma folate levels >25 nmol/L associated with a substantial reduction in NTD risk were achieved in 100% of American and Australian population studies with mandatory fortification, 15% of European studies with voluntary fortification and 7% of Chinese studies with no folic acid fortification, respectively.

Randomised trials demonstrated that folic acid is remarkably effective for prevention of NTD incidence.^2^ Observational studies have demonstrated reductions in the incidence of NTDs in individual countries after introduction of mandatory folic acid fortification. However, the magnitude of any benefits is likely to be underestimated by incomplete screening for NTDs or incomplete reporting of terminations of affected pregnancies with NTDs.^3^ The present analysis of a systematic review of prevalence of NTDs demonstrated that the mean (95% CI) NTD prevalence rates per 10,000 population were 4.19 (4.11–4.28), 7.61 (7.47–7.75) and 9.66 (9.52–9.81) in populations with mandatory, voluntary and no folic acid fortification, respectively.

While the present ecological study cannot make inferences on the causal relevance of associations of fortification with NTD, the findings suggest that mandatory folic acid fortification is associated with a greater reduction in NTD prevalence than voluntary folic acid fortification or no fortification. Compared with voluntary fortification, mandatory folic acid fortification is also associated with a more even distribution of plasma folate levels within populations, narrowing the gap between NTD rates in rich and poor groups. Some national nutrition surveys conducted in the USA reported that mean plasma folate levels before and after implementation of folic acid fortification in the USA reflects dietary folate, added synthetic folic acid by fortification and greater use of folic acid supplements.^46^ The present study demonstrated substantial differences in plasma folate levels before and after fortification, but could not distinguish the relative contributions of folic acid food fortification from use of folic acid supplements.

This review indicated a plateau in the implementation of mandatory fortification policies over the decade prior to July 2023. Prompted by concerns about the lack of efficacy of voluntary folic acid fortification, New Zealand recently legislated to implement mandatory folic acid fortification (July 19, 2023).^47^ Likewise, the Scientific Advisory Committee on Folate in the UK in 2017 advocated upgrading from voluntary fortification to mandatory folic acid fortification to be accompanied by imposition of an upper limit on the daily dose of folic acid supplements that can be consumed without a medical prescription.^5^ In September 2021, the UK government announced that mandatory folic acid fortification of non-wholemeal flour would be introduced, but this has not yet been implemented.^48^ Some experts have advocated that the proposed folic acid fortification to be introduced in the UK should include a broader range of staple foods, including all types of flour (whole meal, gluten-free and white flour) in addition to rice in order to maximise reductions in NTD rates, and provide protection for minority groups who consume rice, gluten-free or wholemeal flour rather than limiting fortification to white flour.^49^

Some reports have suggested that mandatory folic acid fortification could cost about $15 per disability-adjusted life year saved in low-income populations, due chiefly to prevention of complications from NTDs, but other reports have demonstrated that folic acid fortification is cost-saving.^50^, ^51^, ^52^ Whilst mandatory folic acid fortification is the most effective method of increasing population mean plasma folate levels, the present study clearly demonstrated that voluntary folic acid fortification is associated with high proportions with sub-optimal plasma folate levels. There was a wide variation in the implementation of voluntary folic acid fortification between countries, which may partly explain its association with lower mean population folate status compared to mandatory fortification in this study.^53^ However, even in high income countries with relatively extensive voluntary fortification, there is still evidence that it is ineffective in maintaining adequate plasma folate levels in individuals from lower socioeconomic groups.^53^

While the present report included an ecological analysis indicating no differential effects on stroke mortality in USA, UK or China that had different folic acid fortification policies, such ecological analyses cannot control for confounding due to other factors influencing stroke. A report on 156,000 adults from the China Kadooie Biobank involving genotype results for *MTHFR C677T* polymorphism and stroke (n = 2240) reported that individuals with TT genotypes for *MTHFR* were associated with 13% higher risks of total stroke (with a 2-fold stronger association with intra-cerebral haemorrhage than with ischaemic stroke) than the reference CC genotype, consistent with the results of the CSPPT trial in China.^13^ Thus, the genetic studies of *MTHFR* and stroke in China and CSPPT trial of folic acid for stroke prevention in China both demonstrated a protective effect of folic acid for stroke prevention.^13^, ^14^, ^15^ After stratification of all large trials of folic acid (>1000 participants) in a meta-analysis of trials, we showed that trials of folic acid conducted in China reduced the risk of stroke, but those in Western populations had no beneficial effects for stroke prevention. The discrepant results reflect major differences in population mean plasma folate levels between such trial populations (Figure S2, Appendix P16).^12^^,^^13^ The randomised evidence is more reliable than ecological studies, but trial results in China involved a single trial and warrant replication to provide more reliable evidence about the effects of folic acid for stroke prevention.^13^

Concerns about possible hazards associated with mandatory folic acid fortification have included promotion of colon or prostate cancer, or acceleration of cognitive decline in older people or unspecified hazards associated with circulating unmetabolised synthetic folic acid or masking of vitamin B_12_ deficiency.^54^ However, the B-Vitamin Treatment Trialists' collaboration demonstrated that supplementation with folic acid for 5 years had no adverse effects on cancers of the colon or prostate.^55^ Low plasma folate levels have also been implicated with accelerated cognitive decline, but randomised trials have also demonstrated no effects of folic acid on cognitive function.^56^ Likewise, there is no evidence that unmetabolised synthetic folic acid is hazardous for health. Moreover, there is no evidence that folic acid fortification could result in ‘masking’ of vitamin B_12_ deficiency.^54^

While the present study provides reliable evidence about the magnitude of differences in folate levels and NTD prevalence, it also had several important limitations. The use of an ecological study design to relate differences in population folate levels with NTD prevalence or stroke mortality was unable to assess the causal relevance of such associations. Importantly, ecological studies are inherently limited and cannot control for confounding by income or other factors that, could also influence disease outcomes. However, this review aimed to collect data from all 193 countries that were member states of WHO to minimise risk of confounding by income. Data on folate levels were obtained from 108 population studies, NTD prevalence from 75 countries, stroke mortality from 3 countries and, hence, the present study is not strictly a worldwide study. Moreover, studies were also restricted to English language publications, and, hence it is possible that it did not include all worldwide studies. To minimise the risk of bias due to differences in folate assay quality, we compared results of plasma folate levels measured by all assays with those measured using microbiological assays. It is possible that the distribution of plasma folate levels may have been skewed in some populations, but there were insufficient data with to conduct sensitivity analyses using median rather than mean folate levels to exclude such effects. Some experts have expressed reservations about use of plasma folate levels rather than red blood cell folate levels to relate with NTD risks in worldwide populations, but the lack of access to red-cell folate assays outside specialist research laboratories is a major obstacle to their wider use.^57^

The results demonstrated substantial heterogeneity in folic acid fortification policies worldwide with differences of 50–100% higher mean plasma folate levels and 25–50% lower NTD prevalence rates between populations with different folic acid fortification policies. The study also demonstrated that low plasma folate levels still persist in many low and middle-income counties. The World Health Assembly in Spring 2023 agreed a resolution to accelerate efforts to prevent micronutrient deficiencies and NTDs through effective folic acid fortification.^58^ Other studies highlighted that mandatory folic acid fortification is required for low and middle income populations to achieve their 2030 Sustainable Development Goal targets.^59^ China recently initiated a consultation on a government proposal to upgrade from voluntary to mandatory folic acid fortification.^60^ Likewise, experts in the UK have advocated that mandatory folic acid fortification should include all types of flour in addition to rice to maximise reduction of NTD risk.^48^ This updated review of all the available evidence on biochemical effects of folic acid fortification policies for prevention of NTDs provides strong support for China and other countries to implement mandatory folic acid fortification and for the UK and other European countries with voluntary fortification to switch to mandatory folic acid fortification for prevention of NTD. While the present report did not show any benefit of folic acid for stroke prevention, the results of the CSPPT and genetic studies provide strong support for another large trial of folic acid with sufficient power to assess effects of folic acid on stroke types (including intracerebral haemorrhage where the genetic studies suggested would have maximum benefit) to provide definitive evidence on the benefits of folic acid for prevention of stroke.

## Contributors

MQ conducted the literature search and data extraction. RC checked the results of the data extraction for all the included studies. JH and PS conducted the statistical analyses of fortification status and plasma folate levels. HP conducted analyses of stroke mortality data. DAB provided overall statistical supervision. ZC provided unpublished data on stroke mortality from China. MQ and RC wrote the first draft of the report and all other authors revised the report and approved the final draft for publication. MQ and RC accessed and verified the underlying data.

## Data sharing statement

All data extracted can be shared with bona fide researchers upon reasonable request.

## Declaration of interests

All authors declare that they have no competing interests.

## Appendix A Supplementary data

Supplementary Method

## References

[1] MRC Vitamin Study Research Group (1991). Prevention of neural tube defects: results of the medical research Council vitamin study. Lancet.

[2] Czeizel A.E., Dudás I. (1992). Prevention of the first occurrence of neural-tube defects by periconceptional vitamin supplementation. N Engl J Med.

[3] Berry R.J., Li Z., Erickson J.D. (1999). Prevention of neural-tube defects with folic acid in China. China-U.S. Collaborative project for neural tube defect prevention. N Engl J Med.

[4] U.S. Department of Health and Human Services (1992). Recommendations for the use of folic acid to reduce the number of cases of spina bifida and other neural tube defects. MMWR Recomm Rep.

[5] Scientific Advisory Committee on Nutrition (SACN) (2017). https://www.gov.uk/government/publications/folic-acid-updated-sacn-recommendations.

[6] Crider K.S., Bailey L.B., Berry R.J. (2011). Folic acid food fortification-its history, effect, concerns, and future directions. Nutrients.

[7] Stockley L., Lund V. (2008). Use of folic acid supplements, particularly by low-income and young women: a series of systematic reviews to inform public health policy in the UK. Public Health Nutr.

[8] Walsh E., Walton J., Hayes E., Hannon E.M., Flynn J.A. (2010). Contribution of fortified foods to nutrient intakes in Irish teenagers aged 13 to 17 years. Proc Nutr Soc.

[9] Hennessy Á., Walton J., Flynn A. (2013). The impact of voluntary food fortification on micronutrient intakes and status in European countries: a review. Proc Nutr Soc.

[10] Jacques P.F., Selhub J., Bostom A.G., Wilson P.W., Rosenberg I.H. (1999). The effect of folic acid fortification on plasma folate and total homocysteine concentrations. N Eng J Med.

[11] Lawrence M.A., Chai W., Kara R., Rosenberg I.H., Scott J., Tedstone A. (2009). Examination of selected national policies towards mandatory folic acid fortification. Nutr Rev.

[12] Clarke R., Halsey J., Lewington S. (2010). Effects of lowering homocysteine levels with B vitamins on cardiovascular disease, cancer, and cause-specific mortality: meta-analysis of 8 randomised trials involving 37 485 individuals. Arch Intern Med.

[13] Huo Y., Li J., Qin X. (2015). Efficacy of folic acid therapy in primary prevention of stroke among adults with hypertension in China: the CSPPT randomised clinical trial. JAMA.

[14] Holmes M.V., Bobak M., Sofat R. (2011). Effect modification by population dietary folate on the association between MTHFR genotype, homocysteine, and stroke risk: a meta-analysis of genetic studies and randomised trials. Lancet.

[15] Bennett D.A., Parish S., Millwood I.Y. (2023). MTHFR and risk of stroke and heart disease in a low-folate population: a prospective study of 156 000 Chinese adults. Int J Epidemiol.

[16] Crider K.S., Devine O., Hao L. (2014). Population and red blood cell folate concentrations for prevention of neural tube defects: a Bayseian model. BMJ.

[17] Daly L.E., Kirke P.N., Molloy A., Weir D.G., Scott J.M. (1995). Folate levels and neural tube defects: implications for prevention. JAMA.

[18] Wald N.J., Law M.R., Morris J.K. (2001). Wald DS, Quantifying the effect of folic acid. Lancet.

[19] Hoey L., McNulty H., Askin N. (2007). Effect of a voluntary food fortification policy on folate, related B vitamin status, and homocysteine in healthy adults. Am J Clin Nutr.

[20] Dickinson C.J. (1995). Does folic acid harm people with vitamin B12 deficiency?. QJM.

[21] Wien T.N., Pike E., Wisløff T., Staff A., Smeland S., Klemp M. (2012). Cancer risk with folic acid supplements: a systematic review and meta-analysis. BMJ Open.

[22] Chan A.L.F., Leung H.W.C., Wang S.F. (2011). Multivitamin supplement use and risk of breast cancer: a meta-analysis. Ann Pharmacother.

[23] CDC (2018). https://www.cdc.gov/ncbddd/folicacid/faqs/faqs-safety.html.

[24] UK Government (2021). https://www.gov.uk/government/consultations/adding-folic-acid-to-flour/outcome/proposal-to-add-folic-acid-to-flour-consultation-response.

[25] Shane B. (2011). Folate status assessment history: implications for measurement of biomarkers in NHANES. Am J Clin Nutr.

[26] Zaganjor I., Sekkarie A., Tsang B.L. (2016 11). Describing the prevalence of neural tube defects worldwide: a systematic literature review. PLoS One.

[27] Countries overview | World Health Organization. https://www.who.int/countries.

[28] Global Fortification Data Exchange | GFDx – providing actionable food fortification data all in one place. https://fortificationdata.org/.

[29] Lawrence M.A., Kripalani K., Preedy V.R., Srirajaskanthan R., Patel V.B. (2013). Handbook of food fortification and health: from concepts to public health applications volume 2.

[30] World Bank country and lending groups – world Bank data help desk. https://datahelpdesk.worldbank.org/knowledgebase/articles/906519-world-bank-country-and-lending-groups.

[31] Page M.J., Moher D., Bossuyt P.M. (2021). PRISMA 2020 explanation and elaboration: updated guidance and exemplars for reporting systematic reviews. BMJ.

[32] Ek J., Magnus E.M. (1981). Plasma and red blood cell folate during normal pregnancies. Acta Obstet Gynecol Scand.

[33] World Health Organization (2012). https://apps.who.int/iris/bitstream/handle/10665/162114/WHO_NMH_NHD_EPG_15.01.pdf?sequence=1&isAllowed=y.

[34] Young M.F., Guo J., Williams A. (2020). Interpretation of vitamin B-12 and folate concentrations in population-based surveys does not require adjustment for inflammation: biomarkers Reflecting Inflammation and Nutritional Determinants of Anemia (BRINDA) project. Am J Clin Nutr.

[35] Haidar J., Melaku U., Pobocik R.S. (2010). Folate deficiency in women of reproductive ages in nine administrative regions of Ethiopia; an emerging public health problem. South African J Clin Nutr.

[36] Vitamin and mineral nutrition information System (VMNIS). https://www.who.int/teams/nutrition-and-food-safety/databases/vitamin-and-mineral-nutrition-information-system.

[37] Ministry of Health (2012). https://www.binasss.sa.cr/opac-ms/media/digitales/Encuesta%20nacional%20de%20nutricin.%20Fascculo%202.%20Micronutrientes.pdf.

[38] (2014). https://www.ecuadorencifras.gob.ec/documentos/web-inec/Estadisticas_Sociales/ENSANUT/MSP_ENSANUT-ECU_06-10-2014.pdf.

[39] Castillo-Lancellotti C., Tur J., Margozzini P. (2013). Serum folate and vitamin b12 in elderly chileans. Results from the national health survey 2009-10. Ann Nutr Metab.

[40] (2012). Encuesta nacional de Salud y nutricion.

[41] Rohner F., Northrop-Clewes C., Tschannen A.B. (2014). Prevalence and public health relevance of micronutrient deficiencies and undernutrition in pre-school children and women of reproductive age in Côte d'Ivoire, West Africa. Public Health Nutr.

[42] Veritas Health Innovation Covidence systematic review software. Melbourne, Australia: Veritas health innovation. https://www.covidence.org/.

[43] Hozo S.P., Djulbegovic B., Hozo I. (2005). Estimating the mean and variance from the median, range, and the size of a sample. BMC Med Res Methodol.

[44] World Health Statistics 2022: monitoring health for the SDGs, sustainable development goals. https://www.who.int/publications-detail-redirect/9789240051157.

[45] Barendregt J.J., Doi S.A., Lee Y.Y., Norman R.E., Vos T. (2013). Meta-analysis of prevalence. J Epidemiol Community Health.

[46] Pfeiffer C.M., Sternberg M.R., Zhang M. (2019). Folate status in the US population 20 y after the introduction of folic acid fortification. Am J Clin Nutr.

[47] Government of New Zealand (2021). https://www.mpi.govt.nz/food-business/bakery-and-grain-based-products/folic-acid-fortification-of-bread/.

[48] Haggarty P. (2021). UK introduces folic acid fortification of flour to prevent neural tube defects. Lancet.

[49] Looi M. (2023). Folic acid: the case to rethink the UK's food fortification plans. BMJ.

[50] Roman P.S., Waitzman N.J., Scheffer R.M. (1995). Folic acid fortification of grain: an economic analysis. Am J Public Health.

[51] Rodrigues V.B., Silva E.N.D., Santos M.L.P. (2021). Cost-effectiveness of mandatory folic acid fortification of flours in prevention of neural tube defects: a systematic review. PLoS One.

[52] Hoddinott J. (2018). The investment case for folic acid fortification in developing countries. Ann N Y Acad Sci.

[53] Laird E.J., O'Halloran A.M., Carey D., O'Connor D., Kenny R.A., Molloy A.M. (2018). Voluntary fortification is ineffective to maintain the vitamin B12 and folate status of older Irish adults: evidence from the Irish Longitudinal Study on Ageing (TILDA). Br J Nutr.

[54] Hoffbrand V. (2023).

[55] Vollset S.E., Clarke R., Lewington S. (2013). Effects of folic acid on overall and site-specific cancer incidence during the randomised trials: meta-analyses of data on 50,000 individuals. Lancet.

[56] Clarke R., Bennett D., Parish S. (2014). On behalf of the B-Vitamin Treatment Trialists' Collaboration. Effects of homocysteine lowering with B vitamins on cognitive aging: meta-analysis of 11 trials with cognitive data on 22,000 individuals. Am J Clin Nutr.

[57] Pfeiffer C.M., Zhang M., Jabbar S. (2018). Framework for laboratory harmonization of folate measurements in low- and middle-income countries and regions. Ann N Y Acad Sci.

[58] New WHA resolution to accelerate efforts on food micronutrient fortification. https://www.who.int/news/item/29-05-2023-new-wha-resolution-to-accelerate-efforts-on-food-micronutrient-fortification.

[59] Kancherla V., Botto L.D., Rowe L.A. (2022). Preventing birth defects, saving lives, and promoting health equity: an urgent call to action for universal mandatory food fortification with folic acid. Lancet Global Health.

[60] Fortified future? China set for mass fortification of staple foods to combat deficiencies – consultation open. https://www.nutraingredients-asia.com/Article/2023/02/13/china-set-for-mass-fortification-of-staple-foods-to-combat-deficiencies-consultation-open.

