# Supplementary material for: Global heterogeneity in folic acid fortification policies and implications for prevention of neural tube defects and stroke: a systematic review
Source: eClinicalMedicine. 2023 Dec 12;67:102366. doi: 10.1016/j.eclinm.2023.102366 (PMC10758734; doi:10.1016/j.eclinm.2023.102366)
Supplement: Supplementary Method [file mmc1.docx]

**Supplementary material for**

**“Global heterogeneity in folic acid fortification policies and implications for prevention of neural tube defects and stroke: a systematic review”**

[Supplementary Methods 2](#_Toc151551287)

[Protocol for a meta-analysis to assess the effects of different folic acid fortification policies on plasma folate levels, prevalence of neural tube defects and trends in stroke mortality 4](#_Toc151551288)

[Figure S1. Fortification status by country and calendar year of initiation of fortification 15](file:///K:\robert%20sec\Shelley\Papers%20for%20submission\QUINN\eClinicalMedicine\Revision%20November%202023\Ready%20for%20circulation%20to%20co-authors-22-11-2023\Supplementary%20material-22-11-2023%20v3.docx#_Toc151551290)

[Figure S2:  Meta−analysis of randomised trials of folic acid and stroke incidence, by folic acid fortification types 16](#_Toc151551291)

[Table S1: Characteristics of folic acid fortification types by country and calendar year 17](#_Toc151551292)

[Table S2: Characteristics of individual studies included in a systematic review of plasma folate levels by folic acid fortification types 23](#_Toc151551293)

[Table S3: Characteristics of individual studies included in a systematic review of the prevalence of NTDs by folic acid fortification types 30](#_Toc151551294)

[References S1: References for individual population studies of plasma folate levels 35](#_Toc151551295)

[References S2: References for studies of NTD prevalence by country, calendar year and fortification types 42](#_Toc151551296)

[References S3: References for individual trials included in a meta-analysis of folic acid trials for prevention of stroke 48](#_Toc151551297)

[Search strategies 49](#_Toc151551298)

# Supplementary Methods

*Systematic review of the prevalence of NTDs by folic acid fortification types*

A previous systematic review published in 2016, searched for studies conducted between January 1990 and July 2014 on the prevalence of NTDs.^1^ Eligible studies included case-control and cross-sectional studies with prevalence of NTDs (defined as anencephaly or spina bifida or encephalocele) or number of NTDs per total births in the study population. Case reports and supplementation trials were ineligible, as were studies that only included anencephaly and or encephalocele or other chromosomal abnormalities or spina bifida occulta or studies involving fewer than 5000 births or studies that only used mortality data or only included pre-natal diagnoses. Data were extracted on the number of NTDs and number of births to estimate prevalence of NTDs. Data were also extracted on year, geographic location and study design. All studies were reclassified by folic acid fortification types by country and calendar year. Data were obtained on 108 studies in 75 different countries (appendix p32-36), and references for the individual studies are provided in References S2 (appendix p46-51). Data on NTD prevalence were obtained and the weighted mean and 95% CI for each country was estimated and calendar year-specific prevalence by folic acid fortification types. Studies with missing 95%CI were excluded. The weighted mean (95%CI) values of NTD prevalence rates were obtained by conducting a meta-analysis of country-specific prevalence and their 95%CI using the double arcsine transformation to stabilise variance by folic acid fortification categories using a previously described method.^2^

*Meta-analysis of the randomised trials of folic acid and stroke incidence*

A previous systematic review of randomized trials of folic acid for prevention of stroke was updated to include any additional trials of folic acid for stroke prevention that were reported prior to 2023 (appendix p18).^3^ Randomized trials were eligible if: (i) they involved a double-blind randomized comparison of B-vitamin supplements containing folic acid vs placebo for the prevention of vascular disease (irrespective of whether any other treatment was administered factorially); (ii) the relevant treatment arms differed only with respect to the intervention to lower homocysteine levels (ie, they were unconfounded); and (iii) the trial involved at least 1000 participants for a scheduled treatment duration of at least 1 year. Unpublished trials were sought through electronic searches and discussions with other experts in the field, but none was found. Stroke was defined as the first occurrence of ischaemic, haemorrhagic, or unclassified stroke. Comparisons were intention-to-treat, time-to-event analyses of first events of a particular type occurring during the scheduled treatment period among all patients allocated to folic acid vs all allocated to the control treatment. The log-rank observed minus expected (*o* − *e*) statistics and their variances (*v*) from each trial were summed to produce, respectively, a grand total observed minus expected statistic (*G*) and its variance (*V*). The 1-step estimate of the logarithm of the event rate ratio is then *G* /*V* with variance 1/ *V* (and 95% CI [*G* /*V* ] ± [1.96/ *V*^1/2^]). For n trials, the χ^2^ statistic for heterogeneity with *n* − 1 degrees of freedom (χ^2^_n − 1_) is *S* − (*G*^2^/ *V*), where S is the sum over all the trials of (*o* − *e*)^2^/ *v*. Data were obtained on 12 trials which were classified by whether they were conducted in populations with mandatory, voluntary or no folic acid fortification, respectively. The references for the 12 individual trials included in this meta-analysis of folic acid trials for prevention of stroke are provided in References S3 (appendix p52).

**Supplementary methods references**

1. Zaganjor I, Sekkarie A, Tsang BL, Williams J, Razzaghi H, Mulinare J, et al. Describing the prevalence of neural tube defects worldwide: a systematic literature review. PloS one. 2016 Apr 11;11(4):e0151586.

2. Barendregt JJ, Doi SA, Lee YY, Norman RE, Vos T. Meta-analysis of prevalence. J Epidemiol Community Health. 2013; 67: 974-978.

3. Clarke R, Halsey J, Lewington S, Lonn E, Armitage J, Manson JE, et al. Effects of Lowering Homocysteine Levels With B Vitamins on Cardiovascular Disease, Cancer, and Cause-Specific Mortality: Meta-analysis of 8 Randomized Trials Involving 37 485 Individuals. Archives of Internal Medicine. 2010;170(18):1622–31.

# Protocol for a meta-analysis to assess the effects of different folic acid fortification policies on plasma folate levels, prevalence of neural tube defects and trends in stroke mortality

Matthew Quinn, Jim Halsey, Paul Sherliker, Derrick Bennett, Robert Clarke,

Nuffield Department of Population Health, University of Oxford

**Introduction**

Neural tube defects are among the most common congenital conditions and account for lifelong disability for affected individuals. Observational studies have reported inverse associations of plasma folate levels with risk of neural tube defects (NTDs), and randomised trials demonstrated that folic acid supplementation reduces the risk of NTDs by over 70%.^1,2^ Hence, women are routinely advised to take peri-conceptual folic acid supplements to prevent NTDs, but since supplements need to be taken prior to, or in the first trimester of pregnancy to prevent NTDs, the efficacy of these recommendations is limited given the high rates of unplanned pregnancies.^3-6^

Voluntary folic acid fortification (the voluntary addition of folic acid by food manufacturers to ready-to-eat breakfast cereals and other products) has been implemented in the UK and other European countries. However, concerns about possible hazards of folic acid fortification have delayed the implementation of mandatory fortification, and the efficacy of voluntary fortification is uncertain.^7-12^ Voluntary folic acid fortification is constrained by concerns about excessive intakes in a minority of the population who frequently consume ready-to-eat breakfast cereals,^13^ fluctuations in the availability of such products in the market,^13,14^ and inadequate coverage of the population.^15^ In contrast, mandatory folic acid fortification (the compulsory addition of folic acid by food manufacturers to wheat flour, maize or rice) provides more uniform plasma folate levels.^11,16-19^ The mandatory fortification of wheat flour with folic acid is associated with 19-55% reductions in the prevalence of NTDs.^18^

Uncertainties about the possible effects of high plasma levels of unmetabolised folic acid, (due to excessive intake of high-dose folic acid supplements), masking of vitamin B12 deficiency anaemia, and possible increased risks of prostate or breast cancer,^7-11^ have delayed the implementation of mandatory fortification in some countries and prompted the implementation of upper tolerable limits of folic acid intake in other countries.^4^ Persistently high incidence rates of NTDs in Hispanic compared to Caucasian populations after the introduction of mandatory wheat flour fortification in the USA have highlighted the need to monitor fortification policies to ensure their efficacy and prompted the addition of folic acid fortification^5^ to corn or maize flour in 2016.^19-21^ Some countries with mandatory fortification have advocated upper limits of folic acid that can be added to reference foods, but such “nutrient level standards” vary considerably between countries resulting in inequalities in the implementation of folic acid fortification policies worldwide. Consequently, such heterogeneity in public health policies results in differences in population mean plasma folate levels in different countries worldwide.^22-24^

Folic acid supplements are also known to lower plasma homocysteine levels, with higher levels of homocysteine linked with higher risks of stroke.(8) However, it is unclear if folic acid fortification has had any beneficial (or harmful) effect on stroke (or other disease outcomes) in adult populations.^25-35^ It is also unclear whether differences in folic acid fortification policies could alter secular trends in age-standardised stroke mortality rates between countries.

The aims of the present study are to conduct a systematic review of the effects of different public health policies on implementation of folic acid fortification on plasma folate levels, NTD prevalence and stroke mortality. The objectives of the proposed study are: (i) to conduct a scoping review of the implementation of folic acid fortification policies worldwide by country, geographic region and population average incomes; and (ii) to assess differences in plasma folate levels by country, calendar year and type of folic acid fortification policies; (iii) assess differences in prevalence of NTDs, by country, calendar year and folic acid fortification; and (iv) to review the effects of folic acid fortification on trends in total stroke mortality in the United states, United Kingdom and China that have high quality data on plasma folate levels and on age-standardised stroke mortality rates.

**Methods *Folic Acid Fortification Policies***

A search strategy will identify legislation on the implementation of folic acid fortification policies was developed by three researchers. A web search using Google Scholar will be conducted by a single primary reviewer using the search terms “National Policy or Legislation”, “Folic Acid”, “Fortification” and “Country Name”. Countries will involve one of the 194 member states of the World Health Organisation and their respective regions identified in the WHO Statistics 2021.^36^ This search will be supplemented by a review of national government websites and a search for legislation on food standards using available resources in the English language.

The search strategy will screen the following data sources for evidence of legislation on implementation of folic acid fortification: (i) Global Fortification Data Exchange, a live database of international fortification policies; (ii) the “Food Fortification Initiative” and; (iii) the “Global Alliance for Improved Nutrition”.^37^ The Handbook for Food Fortification and Health is a resource involving a review of mandatory folic acid fortification policies published in 2013, and it includes a list of countries with policies on mandatory fortification.^38^ In all cases, when a fortification policy reference is identified for one of these sources, a secondary source will be sought to confirm the implementation of fortification. If a secondary source is not identified, then it will be assumed that such countries do not have a fortification policy in place.

**Data extraction**

Countries will be classified using the World Bank income categories as high income, upper middle income, lower middle income and low income countries.^39^ Publicly available data on national policies on folic acid fortification of wheat flour will be extracted from the Global Fortification Data Exchange and the Handbook for Food Fortification and Health, in addition to other online sources. All identified mandatory and voluntary folic acid fortification policies introduced by national governments prior to July 2023 will be included. Data will be extracted onto a Microsoft Excel spreadsheet with pre-defined data extraction categories. The literature review will include documentation of changes in folic acid fortification policies, data from several countries will be collected on multiple occasions to document changes in fortification policies. Inclusion of the nutrient level standards will be added when these are implemented to yield upper and lower limits for addition of folic acid to food products. Details of the data to be extracted are shown in Table 1.

| **Data extraction** | **Description** |
| --- | --- |
| Country | Country of fortification policy |
| Income status | Income status as per World Bank |
| Food vehicle | Wheat flour/maize/rice |
| Legislation status of folic acid fortification | Mandatory/voluntary/none |
| Nutrient level in standard (mg/kg) | Recommended level of fortification |
| Nutrient level standard | Upper and lower limits of folic acid per kg |
| Year of legislation | Year in which policy was implemented |
| Legislation source | Primary source of legislation |

**Table 1. Data extraction fields for fortification policies**

Several countries with voluntary fortification do not have specific standards for fortification, and the level of folic acid will be defined as a “significant amount” for a product and used as a proxy fortification standard in such countries. The nutrient level in standards is defined as the proportion of the recommended daily allowance or nutrient reference value of folic acid for a healthy adult. In the European Union and the European Economic Area for example, a product with 15% of the nutrient reference value for folic acid (200 μg/100g) is considered to contain a “significant amount” of folic acid.^40^ For the purposes of comparison, this would be calculated as being equivalent to 0.3 mg/kg of folic acid per reference substance.

**Mean plasma folate levels**

A scoping review of the literature will be conducted to collect data on mean serum or plasma folate levels in healthy adults aged 15 years or older and excluding pregnant women. Studies will include results of national surveys or control arms of randomised trials or case-control studies. Serum or plasma folate levels will be selected as indicators of measures of population intake of folic acid. Plasma or serum folate levels will used as such assays provide comparable folate levels between populations. However, serum and plasma folate levels are highly correlated with red blood cell folate levels (which reflect longer-term folate status).^41^ Pregnant women are to be excluded as plasma folate levels decline rapidly during pregnancy, and hence are unrepresentative of the wider population.^42^ Children and adolescents aged less than 15 years are also excluded.^42^ Adolescents aged greater than 15 years will be included as this is the lower limit for women of reproductive age, and the lower age limit included in many large national surveys of population folate status.^42,43^

**Search strategy**

National surveys of folate status will be sought using relevant keywords, Medline (Ovid), Embase (Ovid) and Global Health (Ovid) databases in calendar years up to July 2023. The search will be limited to studies conducted between 01 January 1990 and 06 July 2023. Conference abstracts and full-text articles will be included for this review. The search strategy will be guided by a librarian and validated by the inclusion of a list of relevant reports independently identified by known experts in the field.

**Study selection**

References will be downloaded into COVIDENCE, an online reference-managing software. After exclusion of duplicate studies, titles and abstracts will be screened for eligibility by a single reviewer using pre-specified inclusion and exclusion criteria (Table 2). Full-text reports which do not include serum or plasma folate levels will be excluded, as will duplicate reports from the same study. Ful Large ongoing population studies, including the NHANES study in the USA which collected data every 2 years since 1999,^44^ may generate a large number of publications eligible for inclusion based on the same individuals. In such studies the publications with the largest sample size will be selected for inclusion to minimise risk of death. Studies which included data from diverse populations will only be included only if the study involved the 1000 or more individuals. Studies that did not distinguish data by individual countries will be excluded, unless they can be grouped into a distinct small regions.

| **Inclusion criteria** | **Exclusion criteria** |
| --- | --- |
| National surveys, cross sectional studies, randomised controlled trial (control arm), case-control study (control arm) | Intervention arm of a trial |
| Presents data on plasma or serum folate samples taken from healthy, non-pregnant individuals aged 15 or over | Pregnant women included in population |
| Data collection ended post-1990 | Study sample not healthy or unrepresentative of population |
| Study sample size >1000* | Data not presented for a single WHO Country ^φ^ |
| English language publication^±^ |  |

**Table 2. Inclusion and exclusion criteria of Literature Review of population studies of folate status.**

**An exception will be made for studies with a population size between 850 -1000 for countries which were not otherwise represented by at least two or more sources of data*

^±^ *An exception will be made if data for the survey in question had already been extracted on to the WHO VMNIS Database.^45^*

Data extraction will be supplemented by the World Health Organisation Micronutrient database (extracted 06/07/2023), which collected data on population micronutrient levels from published reports and databases.^45^ Likewise, data extraction will be supplemented by previous published reports that included a plasma folate levels by country and calendar year or assessing the effects of *MTHFR* polymorphism and references of identified studies.^45^

*NTD prevalence rates*

Data on country-specific NTD prevalence by calendar year will be obtained from a previously reported country-specific systematic review of NTD prevalence by calendar year.^46^ All studies were reclassified by folic acid fortification types by country and calendar year. Studies with missing 95%CI were excluded. The weighted mean (95%CI) values of NTD prevalence rates were obtained by conducting a meta-analysis of country-specific prevalence and their 95%CI using the double arcsine transformation to stabilise variance by folic acid fortification categories using a previously described method.^47^

*Trends in stroke mortality rates*
Annual age standardised stroke mortality rates by sex and calendar year for the United States of America, United Kingdom and China among adults aged 40-79 years will be extracted from World Health Organisation mortality databases and United Nations population tables. Mortality rates will be calculated as the mean of the annual rates in seven component 5-year age groups, and standardised for age by averaging rates across all age groups.

**Data extraction**

Data will be extracted from all eligible studies by a single primary reviewer onto a Microsoft Excel spreadsheet and checked by a 2^nd^ reviewer. Data extraction fields will include pre-defined prior to the start of the review. The selection criteria for included studies and data extracted will be moderated by a 2^nd^ reviewer.

| **Extraction Field** | **Description** |
| --- | --- |
| Country | Country of data collection |
| Representativeness | National/Regional (if regional specify area) |
| Area Covered | Urban/Rural/Both Urban and Rural |
| Publication date | Year |
| Start and end date of data collection | Year |
| Type of study | General population surveys/Observational Studies/Case-Control Study (Control)/RCT (Control)/Systematic Review/Meta-Analyses of Observational Studies |
| Population size | Number of participants in study relevant to search question |
| Characteristics | Age range, gender |
| Sample type | Blood, serum ,plasma |
| Averages and distribution data | Arithmetic mean, geometric mean, median, Range, IQR, 95% CI, SD, SE |
| Units | ng/ml, nmol/l, mmol/l, mg/dl |
| Method of analysis | Microbiological Assay, Electro-chemiluminescence, Protein binding assay, Radioimmunoassay (RIA), High Performance Liquid Chromatography |

**Table 3. Data extraction fields for mean plasma folate levels**

Serum and plasma folate values will be extracted as reported in the source documents. Where no information on time period of data collection are available, an assumption will be made that the last year of data collection was the year of study publication. Where no lower age limit is specified in an adult population it will be assumed to be 18 years of age.

**Data synthesis and analysis**

Serum and plasma folate will be treated as being equivalent. All plasma folate values will be converted into nmol/L units and geometric means and median values will be converted into arithmetic means with standard deviation where possible. Overall, arithmetic means and standard deviations will be generated by country, in addition to the arithmetic mean for each country by decade (1990 – 1999, 2000 – 2009, 2010 – 2023) where these data are available. An overall arithmetic mean value will be estimated for each region, income group and fortification type. Population samples will be assumed to have a normal distribution. Sensitivity analyses will restricted to an analysis of folate levels measured using a microbiological assay.

**Statistical analyses**

We will pool the mean plasma folate levels using weighted average of study-specific levels by country band calendar year using inverse-variance weighted fixed-effects meta-analysis. Heterogeneity will be assessed using the I² statistic (I² >50% was considered significant heterogeneity). Contour enhanced funnel plots will be constructed to assess publication bias.

**References**

1. MRC VITAMIN STUDY RESEARCH GROUP. Prevention of neural tube defects: Results of the Medical Research Council Vitamin Study. The Lancet. 1991 Jul 20;338(8760):131–7.

2. Czeizel AE, Dudás I. Prevention of the First Occurrence of Neural-Tube Defects by Periconceptional Vitamin Supplementation. N Engl J Med. 1992 Dec 24;327(26):1832–5.

3. Peake JN, Copp AJ, Shawe J. Knowledge and periconceptional use of folic acid for the prevention of neural tube defects in ethnic communities in the United Kingdom: systematic review and meta-analysis. Birth Defects Res A Clin Mol Teratol. 2013 Jul;97(7):444–51.

4. Recommendations for the use of folic acid to reduce the number of cases of spina bifida and other neural tube defects. MMWR Recomm Rep. 1992 Sep 11;41(RR-14):1–7.

5. Berry RJ, Li Z, Erickson JD, Li S, Moore CA, Wang H, et al. Prevention of neural-tube defects with folic acid in China. China-U.S. Collaborative Project for Neural Tube Defect Prevention. N Engl J Med. 1999 Nov 11;341(20):1485–90.

6. Stockley L, Lund V. Use of folic acid supplements, particularly by low-income and young women: a series of systematic reviews to inform public health policy in the UK. Public Health Nutrition. 2008/08/01 ed. 2008;11(8):807–21.

7. Dickinson CJ. Does folic acid harm people with vitamin B12 deficiency? QJM. 1995 May;88(5):357–64.

8. Wien TN, Pike E, Wisløff T, Staff A, Smeland S, Klemp M. Cancer risk with folic acid supplements: a systematic review and meta-analysis. BMJ Open. 2012 Jan 1;2(1):e000653.

9. Chan ALF, Leung HWC, Wang S-F. Multivitamin supplement use and risk of breast cancer: a meta-analysis. Ann Pharmacother. 2011 Apr;45(4):476–84.

10. Kim Y-I. Folate and carcinogenesis: evidence, mechanisms, and implications. The Journal of Nutritional Biochemistry. 1999 Feb 1;10(2):66–88.

11. CDC. Folic Acid Safety, Interactions, and Effects on Other Outcomes [Internet]. Centers for Disease Control and Prevention. 2018 [cited 2021 Oct 1]. Available from: https://www.cdc.gov/ncbddd/folicacid/faqs/faqs-safety.html

12. National Center on Birth Defects and Developmental Disabilities, Centers for Disease Control and Prevention, Centers for Disease Control and Prevention. Folic Acid Fortification and Supplementation [Internet]. Centre for Disease Control and Prevention. 2017 [cited 2021 Sep 30]. Available from: https://www.cdc.gov/ncbddd/folicacid/faqs/faqs-fortification.html

13. Walsh E, Walton J, Hayes E, Hannon EM, Flynn JA. Contribution of fortified foods to nutrient intakes in Irish teenagers aged 13 to 17 years. Proceedings of the Nutrition Society. 2010/09/09 ed. 2010;69(OCE5):E384.

14. Hennessy A, Walton J, Flynn A. The impact of voluntary food fortification on micronutrient intakes and status in European countries: A review. Proceedings of the Nutrition Society. 2013;72(4):433–40.

15. Scientific Advisory Committee on Nutrition (SACN). Folic acid: updated SACN recommendations [Internet]. Gov.Uk. 2021 [cited 2021 Sep 30]. Available from: https://www.gov.uk/government/publications/folic-acid-updated-sacn-recommendations

16. Hoey L, McNulty H, Askin N, Dunne A, Ward M, Pentieva K, et al. Effect of a voluntary food fortification policy on folate, related B vitamin status, and homocysteine in healthy adults. The American journal of clinical nutrition. 2007;86(5):1405–13.

17. Government of New Zealand. Fortification of flour with the B vitamin folic acid [Internet]. 2021. Available from: https://www.mpi.govt.nz/food-business/bakery-and-grain-based-products/folic-acid-fortification-of-bread/

18. UK Government. Proposal to add folic acid to flour: consultation response [Internet]. Gov.Uk. 2021 [cited 2021 Sep 30]. Available from: https://www.gov.uk/government/consultations/adding-folic-acid-to-flour/outcome/proposal-to-add-folic-acid-to-flour-consultation-response

19. Crider KS, Bailey LB, Berry RJ. Folic acid food fortification-its history, effect, concerns, and future directions. Nutrients. 2011/03/15 ed. 2011 Mar;3(3):370–84.

20. Fleischman AR, Oinuma M. Fortification of corn masa flour with folic acid in the United States. Am J Public Health. 2011/06/16 ed. 2011 Aug;101(8):1360–4.

21. FDA. FDA approves folic acid fortification of corn masa flour [Internet]. 2016 [cited 2021 Sep 30]. Available from: https://www.fda.gov/news-events/press-announcements/fda-approves-folic-acid-fortification-corn-masa-flour

22. Graydon JS, Claudio K, Baker S, Kocherla M, Ferreira M, Roche-Lima A, et al. Ethnogeographic prevalence and implications of the 677C>T and 1298A>C MTHFR polymorphisms in US primary care populations. Biomark Med. 13(8):649–61.

23. Rogers LM, Cordero AM, Pfeiffer CM, Hausman DB, Tsang BL, De-Regil LM, et al. Global folate status in women of reproductive age: a systematic review with emphasis on methodological issues. Ann N Y Acad Sci. 2018/09/21 ed. 2018 Nov;1431(1):35–57.

24. World Health Organization. Serum and red blood cell folate concentrations for assessing folate status in populations [Internet]. WHO; 2012 p. 1–5. Report No.: WHO/NMH/NHD/EPG/12.1. Available from: https://www.who.int/vmnis/indicators/serum_RBC_folate.pdf

25. Clarke R, Halsey J, Lewington S, Lonn E, Armitage J, Manson JE, et al. Effects of Lowering Homocysteine Levels With B Vitamins on Cardiovascular Disease, Cancer, and Cause-Specific Mortality: Meta-analysis of 8 Randomized Trials Involving 37 485 Individuals. Archives of Internal Medicine. 2010 Oct 11;170(18):1622–31.

26. Walsh E, Walton J, Hayes E, Hannon EM, Flynn JA. Contribution of fortified foods to nutrient intakes in Irish teenagers aged 13 to 17 years. Proceedings of the Nutrition Society. 2010/09/09 ed. 2010;69(OCE5):E384.

27. Hennessy Á, Walton J, Flynn A. The impact of voluntary food fortification on micronutrient intakes and status in European countries: a review. Proceedings of the Nutrition Society. 2013/09/11 ed. 2013;72(4):433–40.

28. Scientific Advisory Committee on Nutrition (SACN). Folic acid: updated SACN recommendations [Internet]. Gov.Uk. 2017 [cited 2021 Sep 30]. Available from: https://www.gov.uk/government/publications/folic-acid-updated-sacn-recommendations.

29. Hoey L, McNulty H, Askin N, Dunne A, Ward M, Pentieva K, et al. Effect of a voluntary food fortification policy on folate, related B vitamin status, and homocysteine in healthy adults. The American journal of clinical nutrition. 2007;86(5):1405–13.

30. Crider KS, Bailey LB, Berry RJ. Folic acid food fortification-its history, effect, concerns, and future directions. Nutrients. 2011/03/15 ed. 2011 Mar;3(3):370–84.

31. Dickinson CJ. Does folic acid harm people with vitamin B12 deficiency? QJM. 1995 May;88(5):357–64.

32. Wien TN, Pike E, Wisløff T, Staff A, Smeland S, Klemp M. Cancer risk with folic acid supplements: a systematic review and meta-analysis. BMJ Open. 2012 Jan 1;2(1):e000653.

33. Chan ALF, Leung HWC, Wang SF. Multivitamin supplement use and risk of breast cancer: a meta-analysis. Ann Pharmacother. 2011 Apr;45(4):476–84.

34. CDC. Folic Acid Safety, Interactions, and Effects on Other Outcomes [Internet]. Centers for Disease Control and Prevention. 2018 [cited 2021 Oct 1]. Available from: https://www.cdc.gov/ncbddd/folicacid/faqs/faqs-safety.html

35. Huo Y, Li J, Qin X, Huang Y, Wang X, Gottesman RF, et al. Efficacy of Folic Acid Therapy in Primary Prevention of Stroke Among Adults With Hypertension in China: The CSPPT Randomized Clinical Trial. JAMA. 2015 Apr 7;313(13):1325–35.

36. World Health Organization. World health statistics 2021: monitoring health for the SDGs, sustainable development goals [Internet]. Geneva: World Health Organization; 2021 [cited 2021 Sep 23]. Available from: https://apps.who.int/iris/handle/10665/34270

37. Global Fortification Data Exchange | GFDx – Providing actionable food fortification data all in one place. [Internet]. [cited 2021 Oct 1]. Available from: https://fortificationdata.org.

38. Lawrence MA, Kripalani K. Profiling National Mandatory Folic Acid Fortification Policy Around the World. In: Preedy VR, Srirajaskanthan R, Patel VB, editors. Handbook of Food Fortification and Health: From Concepts to Public Health Applications Volume 2 [Internet]. New York, NY: Springer; 2013 [cited 2021 Aug

39. World Bank Country and Lending Groups – World Bank Data Help Desk [Internet]. [cited 2021 Oct 1]. Available from: https://datahelpdesk.worldbank.org/knowledgebase/articles/906519-world-bank-country-and-lending-groups

40. EUR-Lex - 32011R1169 - EN - EUR-Lex [Internet]. [cited 2021 Oct 1]. Available from: https://eur-lex.europa.eu/legal-content/EN/ALL/?uri=CELEX%3A32011R1169.

41. Galloway M, Rushworth L. Red cell or serum folate? Results from the National Pathology Alliance benchmarking review. J Clin Pathol. 2003 Dec;56(12):924–6.

42. Jaffe JP, Schilling RF. Erythrocyte folate levels: a clinical study. Am J Hematol. 1991; 32: 116–21.

43. Ek J, Magnus EM. Plasma and Red Blood Cell Folate During Normal Pregnancies. Acta Obstetricia et Gynecologica Scandinavica. 1981;60(3):247–51.

44. National Health and Nutrition Examination Survey | Healthy People 2020 [Internet]. [cited 2021 Oct 1]. Available from: https://www.healthypeople.gov/2020/data-source/national-health-and-nutrition-examination-survey.

45. Vitamin and Mineral Nutrition Information System (VMNIS) [Internet]. [cited 2021 Oct 1]. Available from: https://www.who.int/teams/nutrition-and-food-safety/databases/vitamin-and-mineral-nutrition-information-system.

46. Zaganjor I, Sekkarie A, Tsang BL, Williams J, Razzaghi H, Mulinare J, et al. Describing the prevalence of neural tube defects worldwide: a systematic literature review. PloS one. 2016 Apr 11;11(4):e0151586.

47. Barendregt JJ, Doi SA, Lee YY, Norman RE, Vos T. Meta-analysis of prevalence. J Epidemiol Community Health. 2013 Nov 1;67(11):974-8.

#
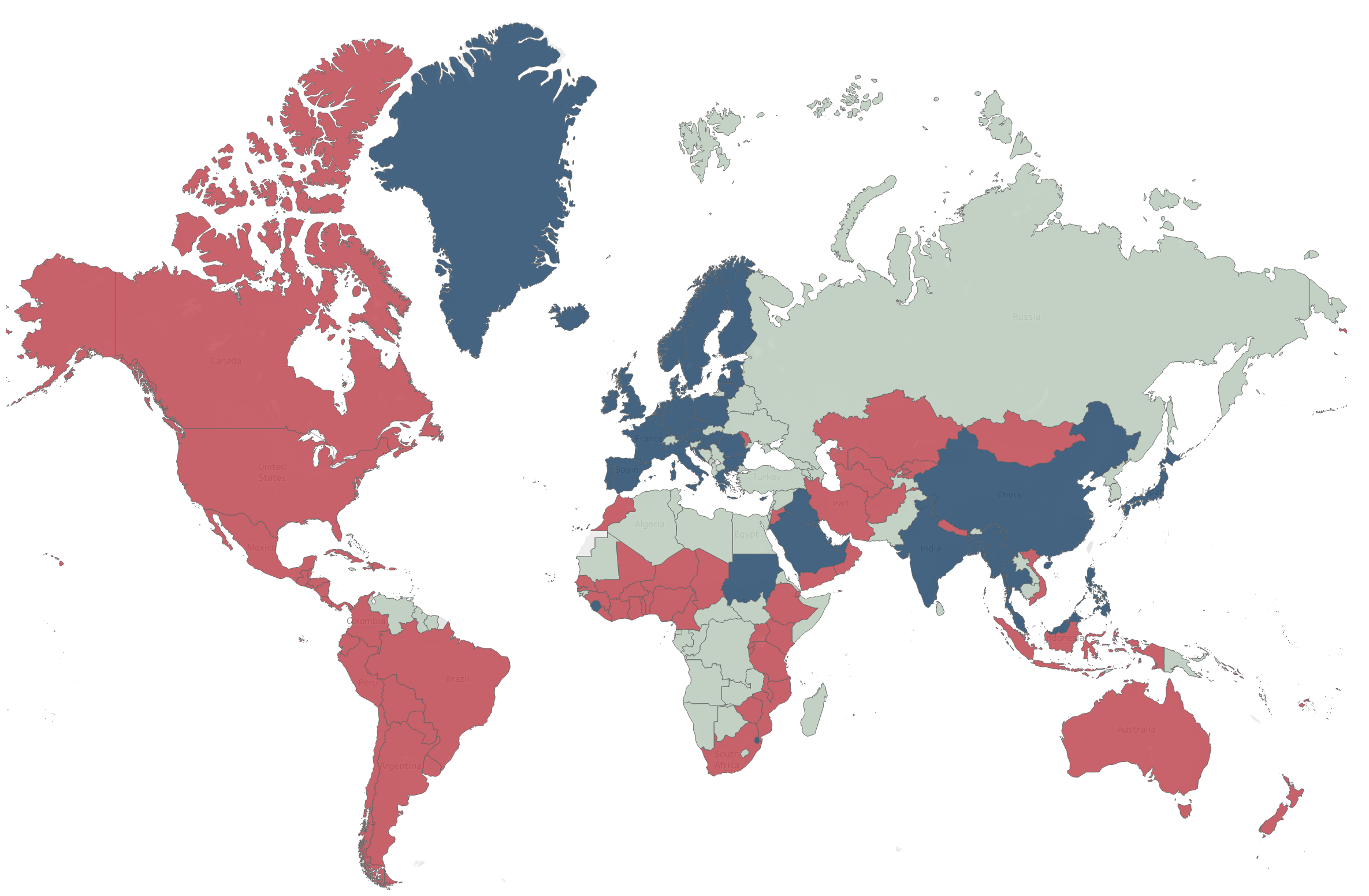

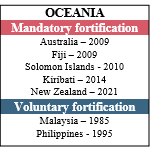

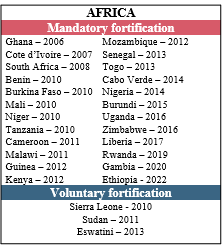

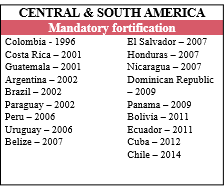

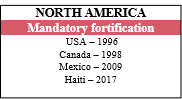

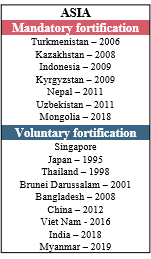

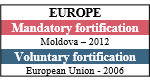

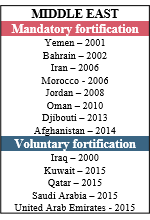


# Figure S1. Fortification status by country and calendar year of initiation of fortification

Mandatory fortification

No fortification

Voluntary fortification

Mandatory fortification

# Figure S2:  Meta−analysis of randomised trials of folic acid and stroke incidence, by folic acid fortification types


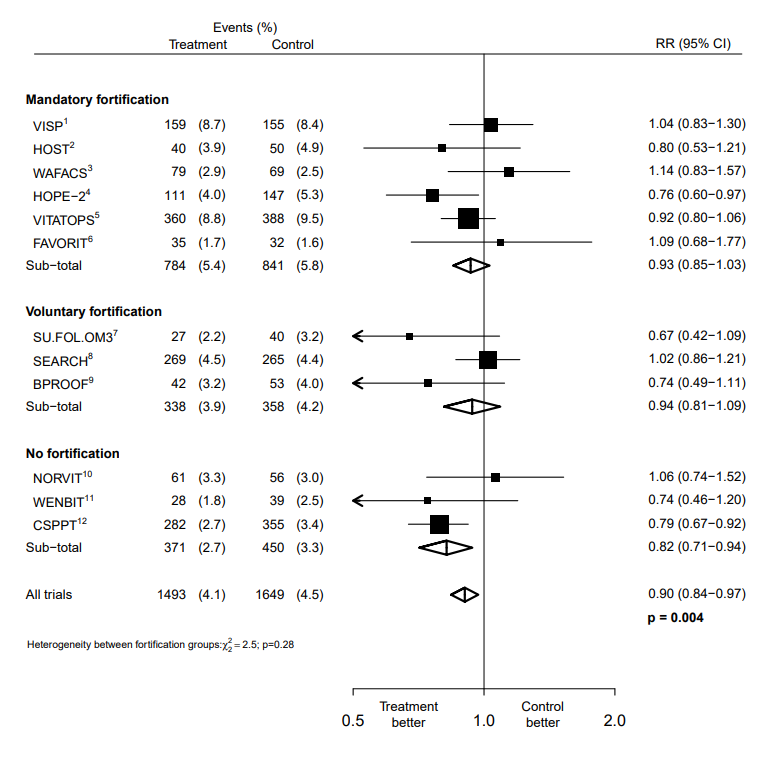


# Table S1: Characteristics of folic acid fortification types by country and calendar year

| **Country** | **Food Vehicle** | **Fortification status** | **Nutrient level in standard (mg/kg)** | **Year of Legislation** |
| --- | --- | --- | --- | --- |
| Afghanistan | Wheat Flour | Mandatory fortification | 1 | 2014 |
| Albania | - | None | - | - |
| Algeria | - | None | - | - |
| Andorra | - | none | - | - |
| Angola | - | None | - | - |
| Antigua and Barbuda | Wheat Flour | None | - | - |
| Argentina | Wheat Flour | Mandatory fortification | 2.2 | 2002 |
| Armenia | - | None | - | - |
| Australia | Wheat Flour | Mandatory fortification | 2.5 | 2009 |
| Austria | - | Voluntary fortification | 0.3 | 2006 |
| Azerbaijan | - | None | - | - |
| Bahamas | Wheat Flour | None | - | - |
| Bahrain | Wheat flour | Mandatory fortification | 1.5 | 2002 |
| Bangladesh | Wheat flour | Voluntary fortification | 2 | 2008 |
| Bangladesh | Rice | Voluntary fortification | 1.7 | 2015 |
| Barbados | Wheat Flour | None | - | - |
| Belarus | - | None | - | - |
| Belgium | - | Voluntary fortification | 0.3 | 2006 |
| Belize | Wheat Flour | Mandatory fortification | 1.8 | 2007 |
| Benin | Wheat flour | Mandatory fortification | 2.5 | 2010 |
| Bhutan | - | None | - | - |
| Bolivia | Wheat Flour | Mandatory fortification | 1.5 | 2011 |
| Bosnia and Herzegovina | - | None | - | - |
| Botswana | - | None | - | - |
| Brazil | Maize | Mandatory fortification | 1.8 | 2002 |
| Brazil | Wheat Flour | Mandatory fortification | 1.8 | 2002 |
| Brunei Darussalam | - | Voluntary Fortification | - | 2001 |
| Bulgaria | - | Voluntary fortification | 0.3 | 2006 |
| Burkina Faso | Wheat flour | Mandatory fortification | 2.5 | 2010 |
| Burundi | Wheat flour | Mandatory fortification | 2.3 | 2015 |
| Burundi | Maize flour | Mandatory fortification | 1.2 | 2015 |
| Cabo Verde | Wheat flour | Mandatory fortification | 2.6 | 2014 |
| Cambodia | - | None | - | - |
| Cameroon | Wheat flour | Mandatory fortification | 5 | 2011 |
| Canada | Wheat flour, Enriched Uncooked Pasta | Mandatory fortification | 1.5 | 1998 |
| Central African Republic | - | None | - | - |
| Chad | Wheat flour | Mandatory fortification | 1.3 | 2021 |
| Chile | Wheat Flour | Mandatory fortification | 2.2 | 2000 |
| Chile | Wheat flour | Mandatory fortification | 1.8 | 2014 |
| China | Wheat flour, Rice | Voluntary fortification | 2 | 2012 |
| Colombia | Wheat flour | Mandatory fortification | 1.54 | 1996 |
| Comoros | - | None | - | - |
| Congo | - | None | - | - |
| Cook Islands | - | None | - | - |
| Costa Rica | Maize flour | Mandatory fortification | 1.3 | 1999 |
| Costa Rica | Wheat Flour | Mandatory fortification | 1.5 | 1997 |
| Costa Rica | Wheat flour | Mandatory fortification | 1.8 | 2001 |
| Costa Rica | Rice | Mandatory fortification | 1.8 | 2002 |
| Côte d'Ivoire | Wheat flour | Mandatory fortification | 1.5 | 2007 |
| Croatia | - | Voluntary fortification | 0.3 | 2006 |
| Cuba | Wheat flour | Mandatory fortification | 1.85 | 2012 |
| Cyprus | - | Voluntary fortification | 0.3 | 2006 |
| Czechia | - | Voluntary fortification | 0.3 | 2006 |
| Democratic People's Republic of Korea | - | None | - | - |
| Democratic Republic of the Congo | - | None | - | - |
| Denmark | - | Voluntary Fortification | 0.3 | 2006 |
| Djibouti | Wheat flour | Mandatory fortification | 1.3 | 2013 |
| Dominica | Wheat Flour | None | - | - |
| Dominican Republic | Maize flour | Voluntary fortification | 1.8 | 2009 |
| Dominican Republic | Wheat flour | Mandatory fortification | 1.8 | 2009 |
| Ecuador | Wheat flour | Mandatory fortification | 1.7 | 2011 |
| Egypt | - | None | - | 2014 |
| Egypt | Wheat Flour | Voluntary fortification | 1.5 | 2008 |
| El Salvador | Wheat flour | Mandatory fortification | 1.8 | 2007 |
| El Salvador | Maize flour | Mandatory fortification | 1 | 2008 |
| Equatorial Guinea | - | None | - | - |
| Eritrea | - | None | - | - |
| Estonia | - | Voluntary Fortification | 0.3 | 2006 |
| Eswatini | Wheat flour | Voluntary fortification | 1.5 | 2013 |
| Ethiopia | Wheat flour | Voluntary fortification | 2 | 2017 |
| Ethiopia | Wheat flour | Mandatory fortification | 2 | 2022 |
| Fiji | Wheat flour | Mandatory fortification | 2 | 2009 |
| Finland | - | Voluntary Fortification | 0.3 | 2006 |
| France | - | Voluntary Fortification | 0.3 | 2006 |
| Gabon | - | None | - | - |
| Gambia | Wheat flour | Mandatory fortification | 2 | 2020 |
| Georgia | - | None | - | - |
| Germany | - | Voluntary Fortification | 0.3 | 2006 |
| Ghana | Wheat flour | Mandatory fortification | 2.08 | 2006 |
| Greece | - | Voluntary fortification | 0.3 | 2006 |
| Grenada | - | None | - | - |
| Guatemala | Maize flour | Mandatory fortification | 1.35 | 2016 |
| Guatemala | Wheat flour | Mandatory fortification | 0.4 | 2001 |
| Guinea | Wheat flour | Mandatory fortification | 1.35 | 2006 |
| Guinea-Bissau | - | None | - | - |
| Guyana | Wheat Flour | None | - | - |
| Haiti | Wheat Flour | Mandatory fortification | - | 2017 |
| Honduras | Wheat flour | Mandatory fortification | 1.8 | 2007 |
| Hungary | - | Voluntary fortification | 0.3 | 2006 |
| Iceland | - | Voluntary fortification | 0.3 | 2006 |
| India | Wheat flour, Rice | Voluntary fortification | 0.1 | 2018 |
| Indonesia | Wheat Flour | Mandatory fortification | - | 2001 |
| Indonesia | Wheat flour | Mandatory fortification | 2 | 2009 |
| Iran (Islamic Republic of) | Wheat Flour | Mandatory fortification | 1.5 | 2006 |
| Iraq | Wheat flour | Voluntary fortification | 2.1 | 2000 |
| Ireland | - | Voluntary fortification | 0.3 | 2006 |
| Israel | - | None | - | - |
| Italy | - | Voluntary fortification | 0.3 | 2006 |
| Jamaica | Wheat Flour | None | - | - |
| Japan | - | Voluntary fortification | 0.6 | [1995](https://www.mhlw.go.jp/stf/seisakunitsuite/bunya/kenkou_iryou/shokuhin/syokuten/index_00012.html) |
| Jordan | Wheat Flour | Mandatory fortification | 1.3 | 2002 |
| Jordan | Wheat flour | Mandatory fortification | 1.52 | 2008 |
| Kazakhstan | Wheat flour | Mandatory fortification | 1.4 | 2008 |
| Kenya | Wheat flour | Mandatory fortification | 1.5 | 2012 |
| Kenya | Maize flour | Mandatory fortification | 1.5 | 2012 |
| Kiribati | Wheat flour | Mandatory fortification | 2 | 2014 |
| Kuwait | Wheat Flour | Mandatory fortification | 1.5 | 2001 |
| Kuwait | Wheat flour | Voluntary fortification | 1.75 | 2015 |
| Kyrgyzstan | Wheat Flour | Mandatory fortification | 1 | 2009 |
| Lao People's Democratic Republic | - | None | - | - |
| Latvia | - | Voluntary fortification | 0.3 | 2006 |
| Lebanon | - | None | - | - |
| Lesotho | - | None | - | - |
| Liberia | Wheat flour | Mandatory fortification | 2.6 | 2017 |
| Libya | - | None | - | - |
| Lithuania | - | Voluntary Fortification | 0.3 | 2006 |
| Luxembourg | - | Voluntary Fortification | 0.3 | 2006 |
| Madagascar | - | None | - | - |
| Malawi | Wheat flour | Mandatory fortification | 2 | 2011 |
| Malawi | Maize flour | Mandatory fortification | 1 | 2011 |
| Malaysia | Meat, Vegetables, Yeast | Voluntary fortification | 1.92 | 1985 |
| Maldives | - | None | - | - |
| Mali | Wheat flour | Mandatory fortification | 2.5 | 2010 |
| Malta | - | Voluntary Fortification | 0.3 | 2006 |
| Marshall Islands | - | None | - | - |
| Mauritania | - | None | - | - |
| Mauritius | - | None | - | - |
| Mexico | Wheat Flour, Maize Flour | Voluntary fortification | 2 | 2001 |
| Mexico | Wheat flour, Maize Flour | Mandatory fortification | 2 | 2009 |
| Micronesia (Federated States of) | - | None | - | - |
| Monaco | - | None | - | - |
| Mongolia | Wheat flour | Mandatory fortification | 1.3 | 2018 |
| Montenegro | - | None | - | - |
| Morocco | Wheat flour | Mandatory fortification | 1.53 | 2006 |
| Mozambique | Wheat flour | Mandatory fortification | 2 | 2012 |
| Mozambique | Maize flour | Mandatory fortification | 2 | 2012 |
| Myanmar | Rice | Voluntary fortification | 1.3 | 2019 |
| Namibia | - | None | - | - |
| Nauru | - | None | - | - |
| Nepal | Wheat flour | Mandatory fortification | 1.5 | 2011 |
| Netherlands |  | Voluntary fortification | 0.3 | 2006 |
| New Zealand | Wheat Flour | Voluntary fortification | 2 | 2012 |
| Nicaragua | Rice | Mandatory fortification | 1 | 2014 |
| Nicaragua | Wheat flour | Mandatory fortification | 1.8 | 2007 |
| Niger | Wheat flour | Mandatory fortification | 2.5 | 2010 |
| Nigeria | Wheat flour | Mandatory fortification | 2.6 | 2014 |
| Nigeria | Maize flour | Mandatory fortification | 2.6 | 2014 |
| New Zealand | Wheat Flour | Mandatory fortification | 2 | 2021 |
| Niue | - |  |  |  |
| North Macedonia | - | None | - | - |
| Norway | - | Voluntary fortification | 0.3 | 2006 |
| Occupied Palestinian Territory | Wheat Flour | Mandatory fortification | 1.5 | - |
| Oman | Wheat Flour | Mandatory fortification | 0.5 | 1996 |
| Oman | Wheat flour | Mandatory fortification | 1.5 | 2010 |
| Pakistan | - | None | - | - |
| Palau | - | None | - | - |
| Panama | Rice | Mandatory fortification | 1 | 2009 |
| Panama | Wheat flour | Mandatory fortification | 1.8 | 2003 |
| Papua New Guinea | - | None | - | - |
| Paraguay | Wheat flour | Mandatory fortification | 3 | 2002 |
| Peru | Rice | Voluntary fortification | 1.2 | 2018 |
| Peru | Wheat flour | Mandatory fortification | 1.2 | 2006 |
| Philippines | - | Voluntary fortification | 1/3 RENI of target consumer/total number of servings consumed in day |  |
| Poland | - | Voluntary Fortification | 0.3 | 2006 |
| Portugal | - | Voluntary Fortification | 0.3 | 2006 |
| Qatar | Wheat flour | Voluntary fortification | 1.75 | 2015 |
| Republic of Korea | - | None | - | - |
| Republic of Moldova | Wheat flour | Mandatory fortification | 1.4 | 2012 |
| Romania | - | Voluntary fortification | 0.3 | 2006 |
| Russian Federation | - | None | - | - |
| Rwanda | Wheat flour | Mandatory fortification | 2.15 | 2019 |
| Rwanda | Maize flour | Mandatory fortification | 1.2 | 2019 |
| Saint Kitts and Nevis | - | None | - | - |
| Saint Lucia | - | None | - | - |
| Saint Vincent and the Grenadines | - | None | - | - |
| Samoa | Wheat flour | Mandatory fortification | 2.0 | 2017 |
| San Marino | - | None | - | - |
| Sao Tome and Principe | - | None | - | - |
| Saudi Arabia | Wheat Flour | Mandatory fortification |  | 1990 |
| Saudi Arabia | Wheat flour | Voluntary fortification | 1.75 | 2015 |
| Senegal | Wheat flour | Mandatory fortification | 2.5 | 2013 |
| Serbia | - | None | - | - |
| Seychelles | - | None | - | - |
| Sierra Leone | Wheat flour | Voluntary fortification | 2.08 | 2010 |
| Singapore | - | Voluntary fortification | - | - |
| Slovakia | - | none | 0.3 | 2006 |
| Slovenia | - | None | 0.3 | 2006 |
| Solomon Islands | Rice | Mandatory fortification | 1.1 | 2018 |
| Solomon Islands | Wheat flour | Mandatory fortification | 2 | 2010 |
| Somalia | - | None | - | - |
| South Africa | Wheat flour | Mandatory fortification | 1.4286 | 2008 |
| South Africa | Maize flour | Mandatory fortification | 2 | 2008 |
| South Sudan | - | None | - | - |
| Spain | - | Voluntary fortification | 0.3 | 2006 |
| Sri Lanka | - | None | - | - |
| Sudan | Wheat flour | Voluntary fortification | 1.3 | 2011 |
| Suriname | - | None | - | - |
| Sweden | - | Voluntary fortification | 0.3 | 2006 |
| Switzerland | - | None | - | - |
| Syrian Arab Republic | - | None | - | - |
| Tajikistan | - | None | - | - |
| Thailand | - | Voluntary fortification | - | 1998 |
| Timor-Leste | - | None | - | - |
| Togo | Wheat flour | Mandatory fortification | 2.6 | 2013 |
| Tonga | - | None | - | - |
| Trinidad and Tobago | - | None | - | - |
| Tunisia | - | None | - | - |
| Turkey | - | None | - | - |
| Turkmenistan | Wheat flour | Mandatory fortification | 1.5 | 2006 |
| Tuvalu | - | None | - | - |
| Uganda | Wheat flour | Mandatory fortification | 2.3 | 2016 |
| Uganda | Maize flour | Mandatory fortification | 1 | 2011 |
| Uganda | Maize flour | Voluntary fortification | 1 | 2006 |
| Ukraine | - | None | - | - |
| United Arab Emirates | Wheat flour | Voluntary fortification | 1.75 | 2015 |
| United Kingdom | - | Voluntary fortification | 0.3 | 2006 |
| United Republic of Tanzania | Wheat flour | Mandatory fortification | 3 | 2010 |
| United Republic of Tanzania | Maize flour | Mandatory fortification | 1.5 | 2011 |
| United States of America | Rice | Mandatory fortification | 1.87 | 1996 |
| United States of America | Wheat Flour | Mandatory fortification | 1.54 | 1996 |
| United States of America | Wheat flour | Mandatory fortification | 1.54 | 2017 |
| United States of America | Rice | Mandatory fortification | 2.31 | 2017 |
| United States of America | Maize flour | Mandatory fortification | 1.87 | 2017 |
| Uruguay | Wheat Flour | Mandatory fortification | 2.4 | 2006 |
| Uzbekistan | Wheat flour | Mandatory fortification | 1 | 2011 |
| Vanuatu | - | None | - | - |
| Venezuela (Bolivarian Republic of) | - | None | - | - |
| Viet Nam | Wheat flour | Mandatory fortification | 5.1 | 2016 |
| Yemen | Wheat Flour | Mandatory fortification | 1.5 | 2001 |
| Zambia | - | None | - | - |
| Zimbabwe | Wheat flour | Mandatory fortification | 2 | 2016 |
| Zimbabwe | Maize flour | Mandatory fortification | 1.3 | 2016 |

# Table S2: Characteristics of individual studies included in a systematic review of plasma folate levels by folic acid fortification types

| **Study identifier** | **Country** | **End Date - Data Collection** | **Fortification status** | **Fortification standard (mg/kg)** | **Type of Study** | **Population Size** | **Age Range (Lower)** | **Age Range (Higher)** | **Gender** | **Sample Type** | **Assay type** | **Mean (nmol/L)** | **SD (nmol/L)** |  |  |  |  |  |  |
| --- | --- | --- | --- | --- | --- | --- | --- | --- | --- | --- | --- | --- | --- | --- | --- | --- | --- | --- | --- |
| 1013 | Australia | 1996 | None | - | Prospective Cohort Study | 558 | 27 | 77 | Male | Blood Serum | Immunoassay | 15.4 | 7.25 |  |  |  |  |  |  |
| 1013 | Australia | 1996 | None | - | Prospective Cohort Study | 553 | 27 | 77 | Female | Blood Serum | Immunoassay | 17.7 | 12.01 |  |  |  |  |  |  |
| 1081 | Australia | 1999 | None | - | Cross Sectional Survey | 1434 | 49 | - | Both | Blood Serum | Competitive Binding Assay | 19.1 | 9.00 |  |  |  |  |  |  |
| 1081 | Australia | 1999 | None | - | Cross Sectional Survey | 234 | 49 | - | Both | Blood Serum | Competitive Binding Assay | 14.4 | 8.10 |  |  |  |  |  |  |
| 1081 | Australia | 1999 | None | - | Cross Sectional Survey | 104 | 40 | - | Both | Blood Serum | Competitive Binding Assay | 13.8 | 8.30 |  |  |  |  |  |  |
| 1209 | Australia | 2006 | None | - | National Survey | 996 | 25 | 34 | Female | Blood Serum | Chemiluminsecent assay | 27.1 | 12.01 |  |  |  |  |  |  |
| 2016 | Australia | 2012 | Mandatory | - | National Survey | 9450 | 18 | - | Both | Blood Serum | Protein Binding Assay | 33.6 | NA |  |  |  |  |  |  |
| 1198 | Azerbaijan | 2013 | None | - | National Survey | 2582 | 15 | 49 | Female | Blood Plasma | Microbial Assay | 11.7 | 0.15 |  |  |  |  |  |  |
| 1170 | Bangladesh | 2009 | Voluntary | 2 | Cross Sectional Study | 275 | 20 | 65 | Male | Blood Plasma | Radioprotein Binding Assay | 17.1 | 15.70 |  |  |  |  |  |  |
| 1170 | Bangladesh | 2009 | Voluntary | 2 | Cross Sectional Study | 252 | 20 | 65 | Female | Blood Plasma | Radioprotein Binding Assay | 16.5 | 12.50 |  |  |  |  |  |  |
| 1170 | Bangladesh | 2008 | Voluntary | 2 | Cross Sectional Study | 167 | 20 | 65 | Male | Blood Plasma | Radioprotein Binding Assay | 11.5 | 6.69 |  |  |  |  |  |  |
| 1170 | Bangladesh | 2008 | Voluntary | 2 | Cross Sectional Study | 175 | 20 | 65 | Female | Blood Plasma | Radioprotein Binding Assay | 14.4 | 7.66 |  |  |  |  |  |  |
| 2002 | Belize | 2011 | Mandatory | 1.8 | National Survey | 937 | 15 | 49 | Female | Blood serum | Microbial Assay | 52.2 | 82.11 |  |  |  |  |  |  |
| 1018 | Canada | 1998 | None | - | Cross Sectional Survey | 3257 | 18 | - | Both | Blood Serum | Competitive protein binding | 18.5 | 0.20 |  |  |  |  |  |  |
| 1018 | Canada | 1998 | None | - | Cross Sectional Survey | 1456 | 18 | - | Both | Blood Serum | Competitive protein binding | 27.2 | 0.36 |  |  |  |  |  |  |
| 1018 | Canada | 1998 | None | - | Cross Sectional Survey | 3257 | 18 | - | Both | Blood Serum | Competitive protein binding | 27.1 | 0.18 |  |  |  |  |  |  |
| 1029 | Canada | 1997 | None | - | Retrospective cross-sectional study | 4572 | 65 | - | Female | Bood Serum | Competitive protein binding | 18.9 | 15.01 |  |  |  |  |  |  |
| 1029 | Canada | 2000 | Mandatory | 1.5 | Retrospective cross-sectional study | 11092 | 65 | - | Female | Bood Serum | Competitive protein binding | 28.2 | 16.91 |  |  |  |  |  |  |
| 1057 | Chile | 2010 | Mandatory | 2.2 | National Survey | 1103 | 65 | - | Both | Blood Plasma | Immunoassay | 49.2 | 21.53 |  |  |  |  |  |  |
| 2003 | Chile | 2010 | Mandatory | 2.2 | National Survey | 823 | 65 | - | Both | Blood Serum | Immunoassay | 48.0 | 21.98 |  |  |  |  |  |  |
| 1009 | China | 2010 | None | - | Prospective Cohort Study | 1605 | 18 | - | Both | Blood Serum | T23 | 21.7 | NA |  |  |  |  |  |  |
| 1030 | China | 2001 | None | - | Cross Sectional Survey | 1202 | 35 | 64 | Both | Blood Serum | microbial assay | 15.9 | 7.73 |  |  |  |  |  |  |
| 3009 | China | 2021 | None | - | Case-Control Study | 1182 | 30 | 75 | Both | Blood serum | HPLC | 19.2 | 10.92 |  |  |  |  |  |  |
| 1077 | China | 2007 | None | - | Randomised controlled trial | 339 | 24 | 42 | Female | Bood Serum | Microbial Assay | 7.3 | 4.54 |  |  |  |  |  |  |
| 1062 | China | 2008 | None | - | Cross Sectional Survey | 2645 | 20 | - | Both | Blood Serum | Microbial Assay | 10.9 | 5.59 |  |  |  |  |  |  |
| 1030 | China | 2001 | None | - | Cross Sectional Survey | 1220 | 35 | 64 | Both | Blood Serum | Microbial Assay | 7.2 | 4.42 |  |  |  |  |  |  |
| 1112 | China | 2006 | None | - | Cross-Sectional Nationwide Survey | 2932 | 18 | 30 | Female | Blood plasma | Not specified | 16.1 | 8.29 |  |  |  |  |  |  |
| 1128 | China | 2012 | None | - | Cross Sectional Survey | 1522 | 19 | 41 | Female | Blood Serum | Not specified | 9.7 | 5.46 |  |  |  |  |  |  |
| 1193 | China | 2004 | None | - | Cross Sectional Survey | 1671 | - | - | Female | Blood Plasma | Not specified | 12.2 | 14.00 |  |  |  |  |  |  |
| 3019 | China | 2006 | None |  | Cross-sectional survey | 3464 | 18 | 45 | Female | Blood plasma | Microbial assay | 14.9 | 9.91 |  |  |  |  |  |  |
| 1097 | China | 2005 | None | - | Randomised controlled trial | 565 | 18 |  | Female | Blood Plasma | Microbial Assay | 7.5 | 5.45 |  |  |  |  |  |  |
| 1077 | China | 2007 | None | - | Randomised controlled trial | 167 | 24 | 42 | Female | Bood Serum | Microbial Assay | 9.3 | NA |  |  |  |  |  |  |
| 1077 | China | 2007 | None | - | Randomised controlled trial | 157 | 24 | 42 | Female | Bood Serum | Microbial Assay | 14.5 | 9.71 |  |  |  |  |  |  |
| 1195 | China | 2012 | None | - | Cross Sectional Survey | 2084 | 25 | - | Female | Blood Plasma | Microbial Assay | 8.5 | 4.74 |  |  |  |  |  |  |
| 1097 | China | 2005 | None | - | Randomised controlled trial | 1108 | 18 |  | Female | Blood Plasma | Microbial Assay | 11.0 | 5.93 |  |  |  |  |  |  |
| 1077 | China | 2007 | None | - | Randomised controlled trial | 338 | 24 | 42 | Female | Bood Serum | Microbial Assay | 11.2 | 6.23 |  |  |  |  |  |  |
| 3017 | China | 2013 | Voluntary | 2 | Cross sectional survey | 11007 | 35 |  | Both | Blood serum | automatic electric lumnescence analyzer | 10.5 | 4.60 |  |  |  |  |  |  |
| 3021 | China | 2019 | Voluntary | 2 | Cross-sectional survey | 4008 | 60 |  | Both | Blood serum | Immunoassay | 17.0 | 6.38 |  |  |  |  |  |  |
| 1059 | China | 2012 | Voluntary | 2 | Cross Sectional Survey | 1845 | 55 | - | Both | Blood Serum | Radioassay kit | 9.5 | 4.95 |  |  |  |  |  |  |
| 1088 | China | 2014 | Voluntary | 2 | Prospective Cohort Study | 1503 | 19 | 65 | Female | Blood Serum | Chemiluminescence Assay | 11.1 | 6.35 |  |  |  |  |  |  |
| 2004 | Costa Rica | 2009 | Mandatory | 1.5 | National Survey | 287 | 65 |  | Both | Blood Serum | Electro-chemiluminescence | 31.5 | 0.68 |  |  |  |  |  |  |
| 2004 | Costa Rica | 2009 | Mandatory | 1.5 | National Survey | 809 | 15 | 44 | Female | Blood Serum | Electro-chemiluminescence | 31.0 | 0.45 |  |  |  |  |  |  |
| 2013 | Côte d'Ivoire | 2007 | Mandatory | 1.5 | National Survey | 853 | 15 | 49 | Female | Blood Plasma | Microbial Assay | 49.9 | 419.83 |  |  |  |  |  |  |
| 1005 | Denmark | 2001 | None | - | Nationwide Survey | 1752 | 30 | 60 | Both | Blood Serum | Chemiluminescent immunoassay | 9.0 | 4.63 |  |  |  |  |  |  |
| 1005 | Denmark | 2008 | Voluntary | 0.3 | Nationwide Survey | 3176 | 18 | 69 | Both | Blood Serum | Chemiluminescent immunoassay | 19.6 | 11.83 |  |  |  |  |  |  |
| 1068 | Eastern Europe | 2005 | None | - | Cross Sectional Study | 4166 | 45 | 69 | Both | Blood Serum | Not specified | 19.7 | 9.06 |  |  |  |  |  |  |
| 2005 | Ecuador | 2012 | Mandatory | 1.7 | National Survey | 439 | 50 | 59 | Female | Blood Serum | Electro-chemiluminescence | 43.5 | 19.71 |  |  |  |  |  |  |
| 2005 | Ecuador | 2012 | Mandatory | 1.7 | National Survey | 1123 | 20 | 29 | Male | Blood Serum | Electro-chemiluminescence | 30.6 | 16.32 |  |  |  |  |  |  |
| 2005 | Ecuador | 2012 | Mandatory | 1.7 | National Survey | 1819 | 40 | 49 | Female | Blood Serum | Electro-chemiluminescence | 40.1 | 19.49 |  |  |  |  |  |  |
| 2005 | Ecuador | 2012 | Mandatory | 1.7 | National Survey | 2531 | 20 | 29 | Female | Blood Serum | Electro-chemiluminescence | 34.4 | 19.03 |  |  |  |  |  |  |
| 2005 | Ecuador | 2012 | Mandatory | 1.7 | National Survey | 2608 | 30 | 39 | Female | Blood Serum | Electro-chemiluminescence | 37.2 | 20.39 |  |  |  |  |  |  |
| 2005 | Ecuador | 2012 | Mandatory | 1.7 | National Survey | 310 | 50 | 59 | Male | Blood Serum | Electro-chemiluminescence | 36.7 | 24.47 |  |  |  |  |  |  |
| 2005 | Ecuador | 2012 | Mandatory | 1.7 | National Survey | 1115 | 30 | 39 | Male | Blood Serum | Electro-chemiluminescence | 31.0 | 15.86 |  |  |  |  |  |  |
| 2005 | Ecuador | 2012 | Mandatory | 1.7 | National Survey | 819 | 40 | 49 | Male | Blood Serum | Electro-chemiluminescence | 34.7 | 16.54 |  |  |  |  |  |  |
| 1216 | Ethiopia | 2005 | None | - | National Survey | 970 | 15 | 49 | Female | Blood Plasma | Enzyme-linked imunosorbent assays | 12.6 | 8.70 |  |  |  |  |  |  |
| 3002 | Ethiopia | 2015 | Voluntary | 2 | National survey | 1647 | 15 |  | Female | Blood serum | Microbial Assay | 11.3 | 8.40 |  |  |  |  |  |  |
| 2006 | Fiji | 2004 | None | - | National Survey | 738 | 15 | 44 | Female | Blood Serum | Protein Binding Assay | 18.0 | 0.26 |  |  |  |  |  |  |
| 2006 | Fiji | 2010 | Mandatory | 2 | National Survey | 869 | 15 | 45 | Female | Blood Serum | Electro-chemiluminescence | 26.6 | 6.45 |  |  |  |  |  |  |
| 1023 | France | 1993 | None | - | Prospective Cohort Study | 1241 | 61 | 73 | Both | Blood Plasma | Immunocompetition | 8.4 | 4.50 |  |  |  |  |  |  |
| 1076 | France | 2001 | None | - | Nationwide Survey | 2933 | 65 | - | Both | Blood Serum | Immunoassay | 14.2 | 8.40 |  |  |  |  |  |  |
| 1076 | France | 2001 | None | - | Nationwide Survey | 981 | 65 | - | Both | Blood Serum | Immunoassay | 16.4 | 10.89 |  |  |  |  |  |  |
| 2010 | France | 2007 | Voluntary | 0.3 | National Survey | 784 | 18 | 74 | Male | Blood Plasma | Protein Binding Assay | 19.3 | 12.27 |  |  |  |  |  |  |
| 2010 | France | 2007 | Voluntary | 0.3 | National Survey | 1318 | 18 | 74 | Female | Blood Plasma | Protein Binding Assay | 15.2 | 7.10 |  |  |  |  |  |  |
| 2014 | Germany | 1999 | None | - | National Survey | 959 | 18 | 40 | Female | Blood Serum | Protein binding assay | 17.2 | NA |  |  |  |  |  |  |
| 1082 | Germany | 2006 | None | - | Retrospective cross-sectional study | 1743 | 18 | - | Both | Blood Serum | Immunoassay | 14.9 | 8.53 |  |  |  |  |  |  |
| 1194 | Guatemala | 2008 | Mandatory | 0.4 | Nationwide Survey | 1448 | 15 | 49 | Female | Blood Serum | Microbial Assay | 37.0 | 26.66 |  |  |  |  |  |  |
| 1086 | India | 2017 | None | - | Cross Sectional Study | 399 | 30 | 65 | Both | - | chemiluminescence | 18.5 | 9.50 |  |  |  |  |  |  |
| 1086 | India | 2017 | None | - | Cross Sectional Study | 975 | 30 | 65 | Both | - | chemiluminescence | 8.4 | 4.60 |  |  |  |  |  |  |
| 1220 | India | 2019 | Voluntary | 0.1 | Cross Sectional Study | 977 | 15 | 40 | Female | Blood Serum | chemiluminescence | 7.7 | 3.61 |  |  |  |  |  |  |
| 1042 | Iran (Islamic Republic of) | 2004 | None | - | Cross Sectional Study | 1214 | 25 | 64 | Both | Blood Serum | Radioassay | 4.5 | 2.46 |  |  |  |  |  |  |
| 1221 | Iran (Islamic Republic of) | 2005 | None | - | Cross Sectional Study | 984 | 20 | - | Both | Blood Serum | Radioimmunoassay | 10.4 | 5.44 |  |  |  |  |  |  |
| 1085 | Ireland | 2004 | None | - | Cross Sectional Survey | 1701 | 18 | 28 | Both | Blood Serum | Microbial Assay | 34.4 | 0.97 |  |  |  |  |  |  |
| 1184 | Ireland | 2012 | Voluntary | 0.3 | Prospective Cohort Study | 1205 | 60 | - | Both | Blood Serum | Microbial Assay | 33.3 | 18.00 |  |  |  |  |  |  |
| 1168 | Ireland | 2010 | Voluntary | 0.3 | Cross Sectional Study | 1115 | 18 | 50 | Both | Blood Serum | Microbial Assay | 25.5 | 16.38 |  |  |  |  |  |  |
| 1208 | Ireland | 2007 | Voluntary | 0.3 | National Survey | 1191 | 45 | - | Both | Blood Serum | Competitive Binding Assay | 18.8 | 10.65 |  |  |  |  |  |  |
| 1007 | Ireland | 2011 | Voluntary | 0.3 | National Survey | 5128 | 50 | 98 | Both | Blood Plasma | Microbial Assay | 24.6 | 26.20 |  |  |  |  |  |  |
| 1007 | Ireland | 2011 | Voluntary | 0.3 | National Survey | 162 | 50 | 98 | Both | Blood Plasma | Microbial Assay | 44.7 | 68.39 |  |  |  |  |  |  |
| 1184 | Ireland | 2012 | Voluntary | 0.3 | Prospective Cohort Study | 1303 | 60 | - | Both | Blood Serum | Microbial Assay | 31.2 | 0.94 |  |  |  |  |  |  |
| 1184 | Ireland | 2012 | Voluntary | 0.3 | Prospective Cohort Study | 1169 | 60 | - | Both | Blood Serum | Microbial Assay | 31.6 | 0.99 |  |  |  |  |  |  |
| 1104 | Israel | 2011 | None | - | Cross Sectional Survey | 4336 | 20 | 80 | Female | Blood Serum | Immunoassay | 22.4 | 10.30 |  |  |  |  |  |  |
| 1104 | Israel | 2011 | None | - | Cross Sectional Survey | 9214 | 20 | 80 | Male | Blood Serum | Immunoassay | 19.2 | 8.60 |  |  |  |  |  |  |
| 1036 | Italy | 1998 | None | - | Cross Sectional Study | 451 | 20 | 102 | Both | Blood Serum | Radioimmunoassay | 5.4 | 0.23 |  |  |  |  |  |  |
| 1036 | Italy | 1998 | None | - | Cross Sectional Study | 439 | 20 | 102 | Both | Blood Serum | Radioimmunoassay | 6.6 | 0.23 |  |  |  |  |  |  |
| 1036 | Italy | 1998 | None | - | Cross Sectional Study | 430 | 20 | 102 | Both | Blood Serum | Radioimmunoassay | 7.5 | 0.29 |  |  |  |  |  |  |
| 1105 | Japan | 2014 | Voluntary | 0.6 | Prospective Cohort Study | 266 | 18 | - | Male | Blood Plasma | Immunoassay | 18.6 | 4.53 |  |  |  |  |  |  |
| 1105 | Japan | 2014 | Voluntary | 0.6 | Prospective Cohort Study | 227 | 18 | - | Male | Blood Plasma | Immunoassay | 21.5 | 6.57 |  |  |  |  |  |  |
| 1105 | Japan | 2014 | Voluntary | 0.6 | Prospective Cohort Study | 266 | 18 | - | Female | Blood Plasma | Immunoassay | 21.1 | 6.80 |  |  |  |  |  |  |
| 1105 | Japan | 2014 | Voluntary | 0.6 | Prospective Cohort Study | 278 | 18 | - | Male | Blood Plasma | Immunoassay | 17.0 | 2.95 |  |  |  |  |  |  |
| 1105 | Japan | 2014 | Voluntary | 0.6 | Prospective Cohort Study | 280 | 18 | - | Female | Blood Plasma | Immunoassay | 19.5 | 4.53 |  |  |  |  |  |  |
| 1105 | Japan | 2014 | Voluntary | 0.6 | Prospective Cohort Study | 223 | 18 | - | Female | Blood Plasma | Immunoassay | 25.6 | 9.97 |  |  |  |  |  |  |
| 1105 | Japan | 2014 | Voluntary | 0.6 | Prospective Cohort Study | 275 | 18 | - | Male | Blood Plasma | Immunoassay | 20.2 | 4.76 |  |  |  |  |  |  |
| 1105 | Japan | 2014 | Voluntary | 0.6 | Prospective Cohort Study | 264 | 18 | - | Female | Blood Plasma | Immunoassay | 22.0 | 4.99 |  |  |  |  |  |  |
| 2012 | Mexico | 2012 | Mandatory | 2 | National Survey | 4029 | 20 | 49 | Female | Blood Serum | Protein Binding Assay | 26.3 | 0.29 |  |  |  |  |  |  |
| 1024 | Netherlands | 1996 | None | - | Prospective Cohort Study | 2051 | 20 | 65 |  | Blood Plasma | Microbial Assay | 12.4 | 4.30 |  |  |  |  |  |  |
| 1022 | Netherlands | 1991 | None | - | Case-Cohort Design | 630 | 20 | 59 | Both | Blood Plasma | Radioassay | 6.3 | NA |  |  |  |  |  |  |
| 1157 | Netherlands | 2001 | None | - | Cross Sectional Study | 1278 | 50 | 70 | Both | Blood Serum | Immunoassay | 12.8 | 4.40 |  |  |  |  |  |  |
| 1045 | Netherlands | 1996 | None | - | Prospective Cohort Study | 1904 | 60 | 90 | Both | Blood Plasma | Microbial Assay | 14.2 | 8.60 |  |  |  |  |  |  |
| 2011 | New Zealand | 2009 | None | - | National Survey | 3277 | 15 |  | Both | Blood Serum | Microbial Assay | 29.0 | 0.56 |  |  |  |  |  |  |
| 2001 | Norway | 2001 | None | - | Prospective Cohort Study | 10601 | 50 | 64 | Both | Blood serum | Microbial Assay | 17.1 | 11.84 |  |  |  |  |  |  |
| 1032 | Norway | 1993 | None | - | Prospective Cohort Study | 1848 | 41 | 67 | Female | Blood Plasma | Microbial Assay | 7.8 | 4.30 |  |  |  |  |  |  |
| 1203 | Norway | 2004 | None | - | Case-Control Study | 3000 | 25 | - | Male | Blood Serum | Novel Serum Folate Assay | 13.7 | 4.48 |  |  |  |  |  |  |
| 1028 | Norway | 1999 | None | - | Prospective Cohort Study | 2949 | 46 | 74 | Both | Blood plasma | Microbial Assay | 8.2 | NA |  |  |  |  |  |  |
| 1028 | Norway | 1999 | None | - | Prospective Cohort Study | 3508 | 46 | 74 | Both | Blood Plasma | Microbial Assay | 8.5 | NA |  |  |  |  |  |  |
| 1028 | Norway | 1999 | None | - | Prospective Cohort Study | 591 | 46 | 74 | Both | Blood plasma | Microbial Assay | 7.5 | NA |  |  |  |  |  |  |
| 1032 | Norway | 1993 | None | - | Prospective Cohort Study | 2047 | 41 | 67 | Female | Blood Plasma | Microbial Assay | 6.9 | 3.17 |  |  |  |  |  |  |
| 1032 | Norway | 1993 | None | - | Prospective Cohort Study | 1449 | 41 | 67 | Male | Blood Plasma | Microbial Assay | 6.8 | 3.43 |  |  |  |  |  |  |
| 1032 | Norway | 1993 | None | - | Prospective Cohort Study | 1650 | 41 | 67 | Male | Blood Plasma | Microbial Assay | 6.7 | 2.84 |  |  |  |  |  |  |
| 1222 | Pakistan | - | None | - | Cross Sectional Study | 218 | 18 | 60 | Both | Blood Serum | Radioassay | 15.4 | 9.97 |  |  |  |  |  |  |
| 1222 | Pakistan | - | None | - | Cross Sectional Study | 218 | 18 | 60 | Both | Blood Serum | Radioassay | 15.6 | 10.88 |  |  |  |  |  |  |
| 1222 | Pakistan | - | None | - | Cross Sectional Study | 218 | 18 | 60 | Both | Blood Serum | Radioassay | 14.3 | 9.97 |  |  |  |  |  |  |
| 1222 | Pakistan | - | None | - | Cross Sectional Study | 218 | 18 | 60 | Both | Blood Serum | Radioassay | 14.5 | 9.74 |  |  |  |  |  |  |
| 2009 | Philippines | 2008 | Voluntary | 0.3 | National Survey | 615 | 30 | 39 | Female | Blood Serum | Protein Binding Assay | 10.8 | 11.53 |  |  |  |  |  |  |
| 2009 | Philippines | 2008 | Voluntary | 0.3 | National Survey | 382 | 40 | 45 | Female | Blood Serum | Protein Binding Assay | 9.7 | 7.20 |  |  |  |  |  |  |
| 2009 | Philippines | 2008 | Voluntary | 0.3 | National Survey | 585 | 20 | 29 | Female | Blood Serum | Protein Binding Assay | 10.4 | 10.60 |  |  |  |  |  |  |
| 1019 | Saudi Arabia | 2002 | None | - | Cross Sectional Survey | 784 | 20 | 69 | Female | Blood Serum | Immunoassay | 14.4 | 5.26 |  |  |  |  |  |  |
| 1019 | Saudi Arabia | 2002 | None | - | Cross Sectional Survey | 642 | 20 | 69 | Male | Blood Serum | Immunoassay | 12.3 | 4.84 |  |  |  |  |  |  |
| 1178 | Senegal | 2010 | None | - | Cross Sectional Survey | 1012 | 15 | 49 | Female | Blood Plasma | Microbial Assay | 10.6 | 7.77 |  |  |  |  |  |  |
| 1197 | Senegal | 2010 | None | - | Cross Sectional Study | 656 | 15 | 49 | Female | Blood Plasma | Microbial Assay | 10.9 | 7.38 |  |  |  |  |  |  |
| 2007 | South Africa | 2005 | None | - | National Survey | 1676 | 16 | 35 | Female | Blood Serum | Protein Binding Assay | 61.9 | 1.50 |  |  |  |  |  |  |
| 3011 | South Korea | 2018 | None |  | National survey | 6394 | 19 | 80 | Both | Blood serum | Immunoassay | 16.7 | 8.11 |  |  |  |  |  |  |
| 1091 | Sweden | 2009 | None | - | Prospective Cohort Study | 1190 | 50 | 70 | Both | Blood Plasma | Microbiological Assay | 17.8 | 13.40 |  |  |  |  |  |  |
| 1003 | Sweden | 2011 | Voluntary | 0.3 | Nationwide Survey | 141 | 18 | 80 | Both | Blood Plasma | Not specified | 7.2 | 4.18 |  |  |  |  |  |  |
| 1003 | Sweden | 2011 | Voluntary | 0.3 | Nationwide Survey | 136 | 18 | 80 | Both | Blood Plasma | Review Riksmaten 2010–2011 | 15.6 | 9.50 |  |  |  |  |  |  |
| 1139 | Taiwan | 1996 | None | - | Nationwide Survey | 85 | 18 | - | Both | Blood Plasma | Microbiological Assay | 25.2 | 10.88 |  |  |  |  |  |  |
| 1037 | Taiwan | 2000 | None | - | Nationwide Survey | 725 | 65 | 90 | Male | Blood Plasma | Immunoassay | 22.9 | 37.70 |  |  |  |  |  |  |
| 1037 | Taiwan | 2000 | None | - | Nationwide Survey | 705 | 65 | 90 | Female | Blood Plasma | Immunoassay | 29.5 | 42.48 |  |  |  |  |  |  |
| 1190 | Taiwan | 2006 | None | - | Nationwide Survey | 825 | 19 | - | Both | Blood Plasma | Immunoassay | 24.7 | 39.05 |  |  |  |  |  |  |
| 3026 | Taiwan | 2020 | None |  | Cross Sectional Survey | 589 | 20 | 49 | Female | Blood serum | chemiluminescent microparticle immunoassay | | | | | | | 23.6 | 17.13 |
| 1139 | Taiwan | 1996 | None | - | Nationwide Survey | 89 | 18 | - | Both | Blood Plasma | Microbiological | 19.0 | 14.28 |  |  |  |  |  |  |
| 1139 | Taiwan | 1996 | None | - | Nationwide Survey | 212 | 18 | - | Both | Blood Plasma | Microbiological | 29.5 | 12.46 |  |  |  |  |  |  |
| 3026 | Taiwan | 2020 | None |  | Cross Sectional Survey | 866 | 20 | 49 | Male | Blood serum | Immunoassay | 17.4 | 17.13 |  |  |  |  |  |  |
| 3014 | Taiwan | 2016 | None |  | Cross sectional study | 1418 | 40 | - | Both | Blood plasma | Immunoassay | 22.2 | 27.55 |  |  |  |  |  |  |
| 1139 | Taiwan | 1996 | None | - | Nationwide Survey | 217 | 18 | - | Both | Blood Plasma | Microbiological | 26.1 | 11.10 |  |  |  |  |  |  |
| 1139 | Taiwan | 1996 | None | - | Nationwide Survey | 161 | 18 | - | Both | Blood Plasma | Microbiological | 18.1 | 7.93 |  |  |  |  |  |  |
| 1139 | Taiwan | 1996 | None | - | Nationwide Survey | 209 | 18 | - | Both | Blood Plasma | Microbiological | 19.7 | 11.10 |  |  |  |  |  |  |
| 2008 | Tajikistan | 2016 | None | - | National Survey | 1903 | 15 | 49 | Female | Blood Serum | Protein Binding Assay | 26.2 | 40.33 |  |  |  |  |  |  |
| 1089 | Tanzania | 2016 | Mandatory | 3 | Cross Sectional Study | 1194 | 18 | 40 | Female | Blood Serum | chemi-luminescence | 30.8 | 13.57 |  |  |  |  |  |  |
| 1001 | Turkey | 2019 | None | - | Cross-Sectional Population Based Study | 9719 | 20 | - | Both | Blood Serum | Not specified | 16.5 | 7.48 |  |  |  |  |  |  |
| 1053 | United Kingdom | 2004 | None | - | Cross-Sectional Study | 939 | 65 | - | Both | Blood Serum | Microbiological Assay | 31.1 | 26.20 |  |  |  |  |  |  |
| 1031 | United Kingdom | 1999 | None | - | Cross Sectional Study | 1006 | 65 | - | Both | Blood Serum | Radioassay | 10.3 | 9.52 |  |  |  |  |  |  |
| 1050 | United Kingdom | 2003 | None | - | Prospective Cohort Study | 472 | 65 | - | Both | Blood Serum | Immunoassay | 12.8 | 11.42 |  |  |  |  |  |  |
| 1053 | United Kingdom | 1999 | None | - | Cross Sectional Study | 1464 | 65 | - | Both | Blood Serum | Competitive Protein Binding | 15.8 | 14.70 |  |  |  |  |  |  |
| 1031 | United Kingdom | 1999 | None | - | Cross Sectional Study | 956 | 65 | - | Both | Blood Serum | Radioassay | 15.0 | 10.38 |  |  |  |  |  |  |
| 1212 | United Kingdom | 2009 | None | - | Case-Control Study | 1507 | 50 | 69 | Male | Blood Plasma | Microbial Assay | 24.7 | 24.46 |  |  |  |  |  |  |
| 1109 | United Kingdom | 2012 | Voluntary | 0.3 | Prospective Cohort Study | 2121 | 60 | 85 | Both | Blood Serum | chemi-luminescence | 15.4 | 10.30 |  |  |  |  |  |  |
| 1160 | United Kingdom | 2008 | Voluntary | 0.3 | Prospective Cohort Study | 461 | 65 | 96 | Male | Blood Serum | Microbial | 15.8 | 14.01 |  |  |  |  |  |  |
| 1160 | United Kingdom | 2008 | Voluntary | 0.3 | Prospective Cohort Study | 442 | 65 | 99 | Female | Blood Serum | Microbial | 16.6 | 10.60 |  |  |  |  |  |  |
| 1026 | United States of America | 1995 | None | - | Cross Sectional Survey | 103 | 65 | - | Male | Blood Serum | Radioassay | 43.5 | 29.23 |  |  |  |  |  |  |
| 1026 | United States of America | 1995 | None | - | Cross Sectional Survey | 109 | 65 | - | Male | Blood Serum | Radioassay | 41.5 | 38.90 |  |  |  |  |  |  |
| 1026 | United States of America | 1995 | None | - | Cross Sectional Survey | 101 | 65 | - | Male | Blood Serum | Radioassay | 49.8 | 53.23 |  |  |  |  |  |  |
| 1026 | United States of America | 1995 | None | - | Cross Sectional Survey | 93 | 65 | - | Female | Blood Serum | Radioassay | 33.1 | 16.09 |  |  |  |  |  |  |
| 1026 | United States of America | 1995 | None | - | Cross Sectional Survey | 97 | 65 | - | Female | Blood Serum | Radioassay | 32.4 | 11.90 |  |  |  |  |  |  |
| 1026 | United States of America | 1995 | None | - | Cross Sectional Survey | 90 | 65 | - | Female | Blood Serum | Radioassay | 44.0 | 53.93 |  |  |  |  |  |  |
| 1026 | United States of America | 1995 | None | - | Cross Sectional Survey | 91 | 65 | - | Female | Blood Serum | Radioassay | 37.1 | 19.33 |  |  |  |  |  |  |
| 1127 | United States of America | 1998 | None | - | Prospective Cohort Study | 1423 | 50 | - | Female | Blood Plasma | Radioimmunoassay | 39.9 | 542.70 |  |  |  |  |  |  |
| 1056 | United States of America | 1993 | None | - | Prospective Cohort Study | 1056 | 55 | 97 | Male | Blood Plasma | Flourescence Detection | 56.3 | 68.20 |  |  |  |  |  |  |
| 1006 | United States of America | 1990 | None | - | Nationwide Survey | 1874 | 30 | - | Female | Blood Plasma | Radioimmunoassay | 30.9 | 33.24 |  |  |  |  |  |  |
| 1017 | United States of America | 1990 | None | - | Cross Sectional Survey | 419 | 68 | 97 | Male | Blood Plasma | Microbiological Assay | 47.1 | 24.53 |  |  |  |  |  |  |
| 1017 | United States of America | 1990 | None | - | Cross Sectional Survey | 657 | 68 | 97 | Female | Blood Plasma | Microbiological Assay | 74.8 | 77.90 |  |  |  |  |  |  |
| 2017 | United States of America | 1994 | None | - | National Survey | 1451 | 20 | 59 | Male | blood serum | Radioimmunoassay | 37.7 | 35.77 |  |  |  |  |  |  |
| 2017 | United States of America | 1994 | None | - | National Survey | 476 | 60 |  | Male | blood serum | Radioimmunoassay | 32.4 | 14.96 |  |  |  |  |  |  |
| 2017 | United States of America | 1994 | None | - | National Survey | 1856 | 20 | 59 | Female | blood serum | Radioimmunoassay | 53.0 | 50.00 |  |  |  |  |  |  |
| 2017 | United States of America | 1994 | None | - | National Survey | 489 | 60 |  | Female | blood serum | Radioimmunoassay | 30.8 | 167.41 |  |  |  |  |  |  |
| 2017 | United States of America | 1994 | None | - | National Survey | 1639 | 20 | 59 | Male | Blood serum | Radioimmunoassay | 67.3 | 36.03 |  |  |  |  |  |  |
| 2017 | United States of America | 1994 | None | - | National Survey | 520 | 60 |  | Male | Blood serum | Radioimmunoassay | 11.3 | 6.72 |  |  |  |  |  |  |
| 2017 | United States of America | 1994 | None | - | National Survey | 1680 | 20 | 59 | Female | Blood serum | Radioimmunoassay | 20.2 | NA |  |  |  |  |  |  |
| 2017 | United States of America | 1994 | None | - | National Survey | 475 | 60 |  | Female | Blood serum | Radioimmunoassay | 14.3 | 10.50 |  |  |  |  |  |  |
| 2017 | United States of America | 1994 | None | - | National Survey | 1648 | 20 | 59 | Male | blood serum | Radioimmunoassay | 9.7 | 6.20 |  |  |  |  |  |  |
| 2017 | United States of America | 1994 | None | - | National Survey | 1411 | 60 |  | male | blood serum | Radioimmunoassay | 8.5 | 10.30 |  |  |  |  |  |  |
| 2017 | United States of America | 1994 | None | - | National Survey | 1972 | 20 | 59 | female | blood serum | Radioimmunoassay | 19.6 | 13.60 |  |  |  |  |  |  |
| 2017 | United States of America | 1994 | None | - | National Survey | 1531 | 60 |  | Female | blood serum | Radioimmunoassay | 17.4 | 15.60 |  |  |  |  |  |  |
| 1026 | United States of America | 1995 | None | - | Cross Sectional Survey | 107 | 65 | - | Male | Blood Serum | Radioimmunoassay | 12.4 | 11.70 |  |  |  |  |  |  |
| 1129 | United States of America | 2010 | Mandatory | 1.54 | Nationwide Survey | 3293 | 40 | 85 | Male | Blood Serum | Microbiological Assay | 9.1 | 9.10 |  |  |  |  |  |  |
| 1113 | United States of America | 2009 | Mandatory | 1.54 | Cross Sectional Survey | 467 | 45 | 75 | Both | Blood Plasma | chemiluminescent assay | 46.9 | 0.92 |  |  |  |  |  |  |
| 1094 | United States of America | 2010 | Mandatory | 1.54 | Nationwide Survey | 1728 | 20 | 49 | Both | Blood Serum | Microbiological Assay | 24.9 | 15.86 |  |  |  |  |  |  |
| 1142 | United States of America | 2003 | Mandatory | 1.54 | Cross Sectional Study | 734 | 30 | 64 | Male | Blood Plasma | Immunoassay | 24.5 | 19.94 |  |  |  |  |  |  |
| 1200 | United States of America | 2018 | Mandatory | 1.54 | Case-Control Study | 3243 | 60 | - | Both | - | - | 13.4 | 17.00 |  |  |  |  |  |  |
| 1002 | United States of America | 2016 | Mandatory | 1.54 | Cross-Sectional Nationwide Survey | 11632 | 20 | 79 | Both | Blood Serum | Not specified | 15.6 | 16.09 |  |  |  |  |  |  |
| 1041 | United States of America | 2000 | Mandatory | 1.54 | Nationwide Survey | 166 | 19 | - | Both | Blood Serum | - | 10.9 | 19.05 |  |  |  |  |  |  |
| 1041 | United States of America | 2000 | Mandatory | 1.54 | Nationwide Survey | 185 | 19 | - | Both | Blood Serum | - | 14.3 | 19.64 |  |  |  |  |  |  |
| 1094 | United States of America | 2010 | Mandatory | 1.54 | Nationwide Survey | 1015 | 20 | 49 | Both | Blood Serum | Microbiological Assay | 12.2 | 17.23 |  |  |  |  |  |  |
| 1041 | United States of America | 2000 | Mandatory | 1.54 | Nationwide Survey | 913 | 19 | - | Both | Blood Serum | - | 18.1 | 17.69 |  |  |  |  |  |  |
| 1041 | United States of America | 2000 | Mandatory | 1.54 | Nationwide Survey | 235 | 19 | - | Both | Blood Serum | - | 27.9 | 24.51 |  |  |  |  |  |  |
| 1113 | United States of America | 2009 | Mandatory | 1.54 | Cross Sectional Survey | 468 | 45 | 75 | Both | Blood Plasma | chemiluminescent assay | 12.3 | 20.24 |  |  |  |  |  |  |
| 1041 | United States of America | 2000 | Mandatory | 1.54 | Nationwide Survey | 757 | 19 | - | Both | Blood Serum | - | 16.1 | 18.24 |  |  |  |  |  |  |
| 1142 | United States of America | 2003 | Mandatory | 1.54 | Cross Sectional Study | 947 | 30 | 64 | Female | Blood Plasma | Immunoassay | 14.0 | 28.69 |  |  |  |  |  |  |
| 1113 | United States of America | 2009 | Mandatory | 1.54 | Cross Sectional Survey | 473 | 45 | 75 | Both | Blood Plasma | chemiluminescent assay | 18.2 | 26.15 |  |  |  |  |  |  |
| 1004 | United States of America | 2006 | Mandatory | 1.54 | Nationwide Survey | 15162 | 18 | - | Both | - | Radioimmunoassay | 7.7 | 24.36 |  |  |  |  |  |  |
| 1006 | United States of America | 2000 | Mandatory | 1.54 | Nationwide Survey | 367 | 30 | - | Female | Blood Plasma | Microbiological Assay | 13.9 | 33.81 |  |  |  |  |  |  |
| 1122 | United States of America | 2000 | Mandatory | 1.54 | Prospective Cohort Study | 371 | 50 | 59 | Male | Blood Plasma | Microbiological Assay | 17.4 | 31.09 |  |  |  |  |  |  |
| 1020 | United States of America | 2001 | Mandatory | 1.54 | Cross Sectional Survey | 1059 | 18 | 45 | Female | Blood Serum | Radioimmunoassay | 27.3 | 43.04 |  |  |  |  |  |  |
| 1041 | United States of America | 2000 | Mandatory | 1.54 | Nationwide Survey | 939 | 19 | - | Both | Blood Serum | - | 28.6 | NA |  |  |  |  |  |  |
| 1033 | United States of America | 1999 | Mandatory | 1.54 | Cross Sectional Survey | 285 | 60 | - | Both | Blood Serum | Radioimmunoassay | 15.5 | 11.10 |  |  |  |  |  |  |
| 3008 | United States of America | 2018 | Mandatory | 1.54 | National Survey | 1479 | 18 |  | Both | Blood serum | HPLC | 38.1 | 54.99 |  |  |  |  |  |  |
| 1041 | United States of America | 2000 | Mandatory | 1.54 | Nationwide Survey | 238 | 19 | - | Both | Blood Serum | - | 58.4 | 81.76 |  |  |  |  |  |  |
| 1033 | United States of America | 1999 | Mandatory | 1.54 | Cross Sectional Survey | 598 | 60 | - | Both | Blood Serum | Radioimmunoassay | 34.2 | NA |  |  |  |  |  |  |
| 3008 | United States of America | 2018 | Mandatory | 1.54 | National Survey | 1318 | 18 |  | Both | Blood serum | Radioimmunoassay | 39.8 | 69.34 |  |  |  |  |  |  |
| 1033 | United States of America | 1999 | Mandatory | 1.54 | Cross Sectional Survey | 527 | 60 | - | Both | Blood Serum | Radioimmunoassay | 30.4 | NA |  |  |  |  |  |  |
| 1033 | United States of America | 1999 | Mandatory | 1.54 | Cross Sectional Survey | 100 | 60 | - | Both | Blood Serum | Radioimmunoassay | 28.6 | NA |  |  |  |  |  |  |
| 1041 | United States of America | 2000 | Mandatory | 1.54 | Nationwide Survey | 785 | 19 | - | Both | Blood Serum | - | 43.6 | 47.63 |  |  |  |  |  |  |
| 1166 | United States of America | 2007 | Mandatory | 1.54 | Prospective Cohort Study | 1546 | 50 | - | Both | Blood Serum | Immunoassay | 34.2 | 22.39 |  |  |  |  |  |  |
| 1214 | Uzbekistan | 1996 | None | - | National Survey | 2563 | 15 | 49 | Female | Blood Serum | Microbiological Assay | 11.9 | 0.13 |  |  |  |  |  |  |
| 1175 | Venezuela | 2001 | None | - | Cross Sectional Study | 1418 | 55 | - | Both | Blood Plasma | Ion Capture Assay | 11.8 | 7.02 |  |  |  |  |  |  |
| 1060 | Viet Nam | 2010 | None | - | Cross Sectional Study | 266 | 15 | 49 | Female | Blood Plasma | Microbiological Assay | 18.0 | 8.15 |  |  |  |  |  |  |
| 1060 | Viet Nam | 2010 | None | - | Cross Sectional Study | 809 | 15 | 49 | Female | Blood Plasma | Microbiological Assay | 18.5 | 11.38 |  |  |  |  |  |  |
| 1060 | Viet Nam | 2010 | None | - | Cross Sectional Study | 286 | 15 | 49 | Female | Blood Plasma | Microbiological Assay | 18.4 | 10.15 |  |  |  |  |  |  |

# Table S3: Characteristics of individual studies included in a systematic review of the prevalence of NTDs by folic acid fortification types

| **Country** | **Fortification type as of 2023**  **(0=none, 1=voluntary, 2=mandatory)** | **Author** | **Ref** | **Year** | **After introduction of folic acid fortification (0=No, 1=Yes)** | **Sum of reported NTDs prevalence per 10,000 pregnancies** | **95% CI** |
| --- | --- | --- | --- | --- | --- | --- | --- |
| Algeria | 0 | Houcher, et al. | 1 | 2006 | 0 | 75.4 | (65.4, 85.4) |
| Argentina | 2 | Groisman B, et al. | 56 | 2012 | 1 | 11.9 | (10.7, 13.2) |
| Argentina | 2 | Lopez-Camelo JS, et al. | 57 | 2007 | 1 | 12.2 | (10.5, 14.0) |
| Australia | 2 | Flood L, et al. | 86 | 2010 | 1 | 19.5 | (13.4, 25.6) |
| Australia | 2 | Macaldowie A and Hilder L | 87 | 2008 | 0 | 8.8 | (8.2, 9.4) |
| Austria | 1 | EUROCAT | 43 | 2009 | 1 | 7.7 | (5.8, 10.0) |
| Bangladesh | 1 | Dey AC, et al. | 73 | 2007 | 0 | 13.8 | (9.2, 20.0) |
| Belgium | 1 | EUROCAT | 43 | 2012 | 1 | 8 | (6.8, 9.3) |
| Belgium | 1 | EUROCAT | 43 | 2012 | 1 | 8.5 | (7.0, 10.3) |
| Brazil | 2 | Orioli IM, et al. | 58 | 2006 | 1 | 1.4 | (1.3, 1.5) |
| Brazil | 2 | Lopez-Camelo JS, et al. | 57 | 2007 | 1 | 24.3 | (21.2, 27.5) |
| Bulgaria | 1 | Kovacheva K, et al. | 44 | 2006 | 1 | 20.2 | (16.2, 24.2) |
| Cameroon | 0 | Njamnshi AK, et al. | 2 | 2006 | 0 | 18.6 | (14.9, 22.3) |
| Canada | 2 | ICBDSR 2011 Report | 20 | 2009 |  | 4.6 | (4.3, 5.0) |
| Chile | 2 | Nazer J and Cifuentes L | 59 | 2010 | 1 | 9.6 | (8.5, 10.7) |
| Chile | 2 | Lopez-Camelo JS, et al. | 57 | 2007 | 1 | 10.1 | (8.8, 11.3) |
| China | 1 | Fan L, et al. | 88 | 2010 | 0 | 5.8 | (3.9, 7.7) |
| China | 1 | Yang M, et al. | 89 | 2008 | 0 | 5.7 | (4.6, 6.8) |
| China | 1 | Li X, et al. | 90 | 2008 | 0 | 14 | (13.4, 14.5) |
| China | 1 | Li X, et al. | 90 | 2008 | 0 | 18.7 | (17.9, 19.5) |
| China | 1 | Li X, et al. | 90 | 2008 | 0 | 9.7 | (9.1, 10.3) |
| China | 1 | Zhang X, et al. | 91 | 2008 | 0 | 20.3^[f]^ | (16.8, 23.8) |
| China | 1 | Dai L, et al. | 92 | 2009 | 0 | 6.5 | (6.1, 6.9) |
| China | 1 | Zhang XH, et al. | 93 | 2009 | 0 | 11.3 | (10.4, 12.2) |
| China | 1 | Chen G, et al. | 94 | 2005 | 0 | 199.4^[d]^ | (165.2, 233.6) |
| China | 1 | Li Y, et al. | 95 | 2009 | 0 | 0.3 | (0.0, 1.2) |
| China | 1 | Liu J, et al. | 96 | 2004 | 0 | 12.2^[d]^ | (10.0, 14.4) |
| China | 1 | Cheng N, et al. | 97 | 2002 | 0 | 66.5 | (46.9, 86.1) |
| China | 1 | Chen BY, et al. | 98 | 2002 | 0 | 2.5 | (1.9, 3.1) |
| Colombia | 2 | Pachajoa H, et al. | 60 | 2008 | 1 | 16.7 | (12.3, 21.1) |
| Colombia | 2 | Zarante I, et al. | 61 | 2008 | 1 | 11 | (8.2, 13.8) |
| Colombia | 2 | ICBDSR 2011 Report | 20 | 2009 | 1 | 3.6 | (1.8, 6.5) |
| **Country** | **Fortification type as of 2023**  **(0=none, 1=voluntary, 2=mandatory)** | **Author** | **Ref** | **Year** | **After introduction of folic acid fortification (0=No, 1=Yes)** | **Sum of reported NTDs prevalence per 10,000 pregnancies** | **95% CI** |
| Costa Rica | 2 | de la Paz Barboza | 63 | 2012 | 1 | 4.8 | (4.3, 5.3) |
| Croatia | 1 | EUROCAT | 43 | 2012 | 1 | 4.5 | (3.1, 6.4) |
| Cuba | 2 | ICBDSR 2011 Report | 20 | 2009 | 0 | 10 | (9.2, 10.8) |
| Czech Republic | 1 | EUROCAT | 43 | 2010 | 1 | 7.6 | (7.0, 8.2) |
| Democratic Republic of Congo | 0 | Ahuka OL, et al. | 3 | 2001 | 0 | 10.2 | (4.7, 19.4) |
| Denmark | 1 | Pasternak B, et al. | 45 | 2011 | 1 | 5.5 | (4.1, 6.8) |
| Denmark | 1 | EUROCAT | 43 | 2012 | 1 | 11.4 | (8.7, 14.7) |
| Ecuador | 2 | Gonzalez-Andrade F and Lopez-Pulles R | 64 | 2007 | 0 | 3.3 | (3.1, 3.5) |
| Egypt | 1 | Mohammed YA, et al. | 12 | 2007 | 0 | 16.0^[b]^ | (6.9, 31.5) |
| Finland | 1 | EUROCAT | 43 | 2011 | 1 | 9 | (8.3, 9.9) |
| France | 1 | Stoll C, et al. | 46 | 2008 | 1 | 10.3^[a]^ | (9.3, 11.3) |
| France | 1 | EUROCAT | 43 | 2002 | 0 | 8.2 | (4.1, 14.7) |
| France | 1 | EUROCAT | 43 | 2012 | 1 | 8.5 | (6.0, 11.8) |
| France | 1 | EUROCAT | 43 | 2012 | 1 | 18.4 | (16.3, 20.7) |
| France | 1 | EUROCAT | 43 | 2003 | 0 | 11.6 | (10.3, 13.0) |
| Germany | 1 | Klusmann A, et al. | 47 | Jul-05 | 0 | 7.1 | (6.5, 7.7) |
| Germany | 1 | EUROCAT | 43 | 2012 | 1 | 14 | (10.0, 19.9) |
| Germany | 1 | EUROCAT | 43 | 2009 | 1 | 9 | (7.6, 10.5) |
| Ghana | 2 | Anyebuno M, et al. | 4 | 1992 | 0 | 11.5 | (7.2, 17.4) |
| Guatemala | 2 | Acevedo CR, et al. | 65 | 2003 | 1 | 27.9 | (25.8, 30.0) |
| Honduras | 2 | Hernandez R and Alvarenga R | 66 | 2000 | 0 | 11.9 | (8.2, 15.5) |
| Hungary | 1 | ICBDSR 2011 Report | 20 | 2011 | 1 | 7 | (6.3, 7.7) |
| India | 1 | Sarkar S, et al. | 74 | 2012 | 0 | 17.8 | (11.3, 26.8) |
| India | 1 | Sood M, et al. | 75 | 1990 | 0 | 66.2 | (49.7, 82.8) |
| India | 1 | Sharma AK, et al. | 76 | 1991 | 0 | 38.8^[d]^ | (35.4, 42.2) |
| India | 1 | Mahadevan B and Bhat BV | 77 | 2004 | 0 | 55.5^[a]^ | (49.3, 61.8) |
| India | 1 | Duttachoudhury A and Pal SK | 78 | 2005 | 0 | 11 | (4.8, 21.8) |
| India | 1 | Ponne Sand Lakshmi UK | 79 | 2004 | 0 | 27.4 | (24.1, 30.7) |
| India | 1 | Grover N | 80 | 1995 | 0 | 44.6 | (31.6, 57.5) |
| India | 1 | ICBDSR 2011 Report | 20 | 2009 | 0 | 26.8 | (25.6, 28.1) |
| India | 1 | Taksande A, et al. | 81 | 2007 | 0 | 7.5 | (3.0, 15.4) |
| Iran | 2 | Ebrahimi S, et al. | 13 | 2011 | 1 | 38.1 | (24.9, 51.3) |
| Iran | 2 | Behrooz AG and Gorjizadeh MH | 14 | 2004 | 0 | 42.2 | (31.2, 53.3) |
| Iran | 2 | Abdollahi Z, et al. | 15 | 2008 | 1 | 21.9 | N/A |
| Iran | 2 | Delshad S, et al. | 16 | 2007 | 1 | 10.1 | (7.6, 12.7) |
| **Country** | **Fortification type as of 2023**  **(0=none, 1=voluntary, 2=mandatory)** | **Author** | **Ref** | **Year** | **After introduction of folic acid fortification (0=No, 1=Yes)** | **Sum of reported NTDs prevalence per 10,000 pregnancies** | **95% CI** |
| Iran | 2 | Rad IA, et al. | 18 | 2005 | 0 | 82.9 | (67.9, 97.8) |
| Iran | 2 | Farhud DD, et al. | 19 | 1997 | 0 | 50.1 | (35.2, 65.0) |
| Iran | 2 | ICBDSR 2011 Report | 20 | 2009 | 1 | 6.5 | (3.6, 10.7) |
| Iraq | 1 | Al-Ani ZR, et al. | 21 | 2011 | 1 | 27.9^[a]^ | (15.9, 45.2) |
| Iraq | 1 | Al-Sadoon I, et al. | 22 | 1990 | 0 | 9.9 | (5.1, 17.2) |
| Ireland | 1 | McDonnell R, et al. | 48 | 2012 | 1 | 10.4 | (9.1, 11.8) |
| Ireland | 1 | EUROCAT | 43 | 2012 | 1 | 11.3 | (9.2, 13.6) |
| Ireland | 1 | EUROCAT | 43 | 2012 | 1 | 5.9 | (5.0, 7.0) |
| Ireland | 1 | EUROCAT | 43 | 2012 | 1 | 8.6 | (6.6, 11.0) |
| Israel | 0 | Zlotogora J, et al. | 49 | 2004 | - |  |  |
| Israel | 0 | Zlotogora J, et al. | 49 | 2004 | - | 8.1 | N/A |
| Israel | 0 | Zlotogora J, et al. | 49 | 2004 | - | 16.7 | N/A |
| Israel | 0 | ICBDSR 2011 Report | 20 | 2009 | - | 4.6 | (3.7, 5.5) |
| Italy | 1 | EUROCAT | 43 | 2012 | 1 | 5.6 | (4.9, 6.4) |
| Italy | 1 | EUROCAT | 43 | 2004 | 0 | 2 | (0.9, 3.9) |
| Italy | 1 | EUROCAT | 43 | 2012 | 1 | 5.7 | (4.9, 6.6) |
| Italy | 1 | ICBDSR 2011 Report | 20 | 2009 | 1 | 7.7 | (6.7, 8.7) |
| Italy | 1 | ICBDSR 2011 Report | 20 | 2009 | 1 | 4.9 | (1.6, 11.5) |
| Italy | 1 | ICBDSR 2011 Report | 20 | 2009 | 1 | 4.5 | (3.7, 5.3) |
| Japan | 1 | Imaizumi Y, et al. | 99 | 1990 | 0 | 8.4 | (5.1, 12.9) |
| Japan | 1 | Seto T, et al. | 100 | 2000 | 1 | 2.6 | (1.7, 3.9) |
| Japan | 1 | ICBDSR 2011 Report | 20 | 2009 | 1 | 6.9 | (6.1, 7.7) |
| Jordan | 2 | Amarin ZO and Obeidat AZ | 23 | 2006 | 0 | 9.5 | (5.5, 15.5) |
| Jordan | 2 | Aqrabawi HE | 24 | 2003 | 0 | 62.9^[a]^ | (41.2, 84.6) |
| Jordan | 2 | Masri AT | 25 | 2002 | 0 | 11.0^[c]^ | (7.1, 14.8) |
| Jordan | 2 | Daoud AS, et al. | 26 | 1993 | 0 | 16.4 | (13.7, 19.1) |
| Kuwait | 2 | Madi SA, et al. | 27 | 2001 | 1 | 6.5 | (2.1, 15.1) |
| Libya | 0 | Singh Rand Al-Sudani O | 28 | 1995 | - | 8 | (4.3, 13.7) |
| Malawi | 2 | Msamati BC, et al. | 5 | 1999 | 0 | 6.3 | (3.6, 10.2) |
| Malaysia | 1 | Boo NY, et al. | 102 | 2009 | 1 | 5.4 | (4.5, 6.2) |
| Malta | 1 | EUROCAT | 43 | 2011 | 1 | 10.2 | (7.2, 14.1) |
| Mexico | 2 | Hernandez-Herrera RJ, et al. | 67 | 1999 | 0 | 16 | (13.9, 18.2) |
| Mexico | 2 | Alfaro N, et al. | 68 | 1999 | 0 | 19.7 | (17.6, 21.8) |
| Mexico | 2 | Navarrete Hernandez E, et al. | 69 | 2010 | 1 | 3.3 | (3.1, 3.5) |
| Mexico | 2 | ICBDSR 2011 Report | 20 | 2009 | 1 | 11.9 | (9.8, 14.1) |
| **Country** | **Fortification type as of 2023**  **(0=none, 1=voluntary, 2=mandatory)** | **Author** | **Ref** | **Year** | **After introduction of folic acid fortification (0=No, 1=Yes)** | **Sum of reported NTDs prevalence per 10,000 pregnancies** | **95% CI** |
| Netherlands | 1 | EUROCAT | 43 | 2012 | 1 | 7.7 | (6.5, 9.1) |
| New Zealand | 2 | ICBDSR 2011 Report | 20 | 2009 | 0 | 3 | (2.4, 3.6) |
| Nigeria | 2 | Ekanem TB, et al. | 6 | 2003 | 0 | 5.2 | (4.0, 6.5) |
| Nigeria | 2 | Airede KI | 7 | 1990 | 0 | 58.6^[a]^ | (39.3, 78.0) |
| Norway | 1 | EUROCAT | 43 | 2012 | 1 | 9.1 | (8.4, 9.9) |
| Oman | 2 | Alasfoor D and ElSayed MK | 29 | 2010 | 1 | 23.2 | N/A |
| Pakistan | 0 | Khattak ST, et al. | 30 | 2007 | - | 124.1^[d]^ | (95.0, 153.2) |
| Pakistan | 0 | Qazi G | 31 | 2009 | - | 68.8 | (46.1, 91.6) |
| Pakistan | 0 | Perveen F and Tyyab S | 32 | 2005 | - | 50.2 | (33.6, 72.1) |
| Pakistan | 0 | Najmi RS | 33 | 1998 | - | 49.3 | (36.3, 62.3) |
| Pakistan | 0 | Jooma R | 34 | 2002 | - | 38.6 | (26.4, 50.9) |
| Palestine | 0 | Dudin A | 106 | 1993 | - | 54.9^[a]^ | (46.1, 63.7) |
| Papua New Guinea | 0 | Dryden R | 103 | 1986 | - | 7 | (2.6, 14.4) |
| Peru | 2 | Sanabria Rojas HA, et al. | 70 | 2010 | 1 | 8.2^[a]^ | (6.3, 10.0) |
| Poland | 1 | EUROCAT | 43 | 2010 | 1 | 5.9 | (5.7, 6.2) |
| Poland | 1 | EUROCAT | 43 | 2010 | 1 | 8.5 | (7.5, 9.6) |
| Portugal | 1 | EUROCAT | 43 | 2011 | 1 | 3.2 | (2.4, 4.2) |
| Qatar | 1 | Bener A, et al. | 35 | 2009 | 0 | 10.9 | (9.7, 12.2) |
| Russia | 0 | Petrova JG and Vaktskjold A | 50 | 2004 | - | 21.1 | (18.7, 23.5) |
| Russia | 0 | ICBDSR 2011 Report | 20 | 2009 | - | 7.6 | (6.6, 8.6) |
| Saudi Arabia | 1 | Al-Jama F, et al. | 36 | 1997 | 1 | 53.5 | (41.7, 65.3) |
| Saudi Arabia | 1 | Asindi A and Al-Shehri A. | 37 | 1997 | 1 | 7.5^[a]^ | (5.6, 9.4) |
| Saudi Arabia | 1 | Safdar OY, et al. | 38 | 2005 | 1 | 7.6 | N/A |
| Saudi Arabia | 1 | Murshid WR | 39 | 1997 | 1 | 10.9 | (6.5, 17.2) |
| Saudi Arabia | 1 | Hakami WS and Majeed-Saidan MA | 40 | 2010 | 1 | 4.5 | (3.2, 5.9) |
| Singapore | 1 | Shi LM, et al. | 104 | 1998 | - | 1.2^[b]^ | (0.8, 1.8) |
| Slovak Republic | 0 | ICBDSR 2011 Report | 20 | 2009 | 1 | 3.8 | (3.1, 4.5) |
| South Africa | 2 | Sayed AR, et al. | 8 | 2005 | 0 | 9.8 | (6.9, 12.7) |
| South Africa | 2 | Venter PA, et al. | 9 | 1992 | 0 | 35.4 | (23.4, 51.6) |
| South Africa | 2 | Viljoen DL, et al. | 10 | 1992 | 0 | 11.7 | (10.8, 12.6) |
| South Korea | 0 | Kim MA, et al. | 101 | 2006 | 0 | 3.1 | (2.7, 3.5) |
| Spain | - | EUROCAT | 43 | 2007 | 1 | 9 | (7.0, 11.4) |
| Spain | - | EUROCAT | 43 | 2011 | 1 | 10 | (8.6, 11.5) |
| Spain | - | EUROCAT | 43 | 2012 | 1 | 1.3 | (1.0, 1.6) |
| Spain | - | EUROCAT | 43 | 2011 | 1 | 6.4 | (5.5, 7.4) |
| **Country** | **Fortification type as of 2023**  **(0=none, 1=voluntary, 2=mandatory)** | **Author** | **Ref** | **Year** | **After introduction of folic acid fortification (0=No, 1=Yes)** | **Sum of reported NTDs prevalence per 10,000 pregnancies** | **95% CI** |
| Sweden | 1 | EUROCAT | 43 | 2011 | 1 | 7.5 | (6.8, 8.3) |
| Switzerland | 0 | Poretti A, et al. | 51 | 2007 | - | 10.7^[a]^ | (8.7, 12.6) |
| Switzerland | 0 | EUROCAT | 43 | 2012 | - | 10.4 | (8.2, 12.9) |
| Tanzania | 2 | Kinasha AD and Manji K | 11 | 2002 | 0 | 30.2 | (24.4, 36.0) |
| Thailand | 1 | Jaruratanasirikul S, et al. | 83 | 2012 | 1 | 1.9 | (1.3, 2.7) |
| Thailand | 1 | Kitisomprayoonkul N and Tongsong T | 84 | 2000 | 1 | 6.6 | (4.7, 8.6) |
| Thailand | 1 | Wasant P and Sathienkijkanchai A | 85 | 1999 | 1 | 6.7[b, d] | (5.5,7.9) |
| Turkey | 0 | Onrat ST, et al. | 52 | 2004 | - | 35.9 | (23.3, 48.5) |
| Turkey | 0 | Mandiracioglu A, et al. | 53 | 2000 | - | 14.3[a, b] | (10.4, 18.2) |
| Turkey | 0 | Tuncbilek E, et al. | 54 | 1994 | - | 30.1 | (22.9, 37.4) |
| Turkey | 0 | Himmetoglu O, et al. | 55 | 1995 | - | 34.9 | (22.6, 46.6) |
| Ukraine | 0 | EUROCAT | 43 | 2011 | - | 17.7 | (16.0, 19.6) |
| United Arab Emirates | 1 | Al Hosani H, et al. | 42 | 2001 | 0 | 2.1^[f]^ | (1.4, 2.8) |
| United Kingdom | 1 | EUROCAT | 43 | 2012 | 1 | 11.3 | (10.5, 12.1) |
| United Kingdom | 1 | EUROCAT | 43 | 2000 | 0 | 16.9 | (14.7, 19.4) |
| United Kingdom | 1 | EUROCAT | 43 | 1999 | 0 | 12.9 | (11.1, 15.0) |
| United Kingdom | 1 | EUROCAT | 43 | 2004 | 1 | 10.9 | (8.9, 13.2) |
| United Kingdom | 1 | EUROCAT | 43 | 2012 | 1 | 13.8 | (12.6, 15.1) |
| United Kingdom | 1 | EUROCAT | 43 | 2012 | 1 | 10.7 | (9.7, 11.7) |
| United Kingdom | 1 | EUROCAT | 43 | 2012 | 1 | 10.8 | (9.6, 12.1) |
| United Kingdom | 1 | EUROCAT | 43 | 2012 | 1 | 13.5 | (12.3, 14.8) |
| United Kingdom | 1 | EUROCAT | 43 | 2012 | 1 | 11.7 | (10.5, 13.0) |
| United States | 2 | Canfield MA, et al. | 72 | 2007 | 1 | 5.3 | (5.1, 5.4) |
| Uruguay | 2 | Castilla EE, et al. | 71 | 2001 | 0 | 17.5 | (11.9, 23.1) |
| Venezuela | 0 | Castilla EE, et al. | 71 | 2001 | - | 14.9 | (11.0, 18.8) |
| Vietnam | 2 | Hoang T, et al. | 105 | 2010 | 0 | 4.3 | (1.6, 9.4) |

# References S1: References for individual population studies of plasma folate levels

1001. Omer B, Telci A, Turker F, Tutuncu Y, Dinccag N, Karsidag K, Yilmaz T, Satman I, Genc S. The prevalence of folate deficiency in the adult population in Turkey: The association with prediabetes and diabetes. Clinica Chimica Acta. 2019;493:S626-7.

1002. Han YY, Forno E, Rosser F, Celedón JC. Serum folate metabolites, asthma, and lung function in a nationwide US study. Journal of Allergy and Clinical Immunology. 2020;146(1):220-2.

1003. Céline S, Johansson I, Hefni M, Witthöft C. Legume consumption in Sweden: a descriptive cross-sectional study. Proceedings of the Nutrition Society. 2020;79(OCE2).

1004. Otero TM, Monlezun DJ, Christopher KB, Camargo CA, Quraishi SA. Vitamin D status and elevated red cell distribution width in community-dwelling adults: Results from the National Health and Nutrition Examination Survey 2001–2006. The journal of nutrition, health & aging. 2017 Dec;21(10):1176-82.

1005. Møllehave LT, Skaaby T, Simonsen KS, Thuesen BH, Mortensen EL, Sandholt CH, Pedersen O, Grarup N, Hansen T, Linneberg A. Association studies of genetic scores of serum vitamin B12 and folate levels with symptoms of depression and anxiety in two danish population studies. European journal of clinical nutrition. 2017 Sep;71(9):1054-60.

1006. Houghton SC, Eliassen AH, Zhang SM, Selhub J, Rosner BA, Willett WC, Hankinson SE. Plasma B-vitamin and one-carbon metabolites and risk of breast cancer before and after folic acid fortification in the US. Cancer Research. 2018 Jul 1;78(13_Supplement):3221-.

1007. Laird EJ, O’Halloran AM, Carey D, O’Connor D, Kenny RA, Molloy AM. Voluntary fortification is ineffective to maintain the vitamin B12 and folate status of older Irish adults: evidence from the Irish Longitudinal Study on Ageing (TILDA). British Journal of Nutrition. 2018 Jul;120(1):111-20.

1009. Long P, Liu X, Li J, He S, Chen H, Yuan Y, Qiu G, Yu K, Liu K, Jiang J, Yang H. Circulating folate concentrations and risk of coronary artery disease: a prospective cohort study in Chinese adults and a Mendelian randomization analysis. The American Journal of Clinical Nutrition. 2020 ;111(3):635-43.

1013. McQuillan BM, Beilby JP, Nidorf M, Thompson PL, Hung J. Hyperhomocysteinemia but not the C677T mutation of methylenetetrahydrofolate reductase is an independent risk determinant of carotid wall thickening: the Perth Carotid Ultrasound Disease Assessment Study (CUDAS). Circulation. 1999 May 11;99(18):2383-8.

1017. Seshadri S, Beiser A, Selhub J, Jacques PF, Rosenberg IH, D'Agostino RB, Wilson PW, Wolf PA. Plasma homocysteine as a risk factor for dementia and Alzheimer's disease. New England journal of medicine. 2002 Feb 14;346(7):476-83.

1018. Ray JG, Vermeulen MJ, Boss SC, Cole DE. Declining rate of folate insufficiency among adults following increased folic acid food fortification in Canada. Canadian journal of public health. 2002 Jul;93(4):249-53.

1019. Ardawi MS, Rouzi AA, Qari MH, Dahlawi FM, Al-Raddadi RM. Influence of age, sex, folate and vitamin B12 status on plasma homocysteine in Saudis. Saudi medical journal. 2002 Aug 1;23(8):959-68.

1020. Than LC, Watkins M, Daniel KL. Serum folate levels among women attending family planning clinics: Georgia, 2000. MMWR Recomm Rep. 2002 Sep 13;51(RR-13):4-8.

1022. de Bree A, Verschuren WM, Blom HJ, Nadeau M, Trijbels FJ, Kromhout D. Coronary heart disease mortality, plasma homocysteine, and B-vitamins: a prospective study. Atherosclerosis. 2003 Feb 1;166(2):369-77.

1023. Dufouil C, Alpérovitch A, Ducros V, Tzourio C. Homocysteine, white matter hyperintensities, and cognition in healthy elderly people. Annals of Neurology: Official Journal of the American Neurological Association and the Child Neurology Society. 2003 Feb;53(2):214-21.

1024. de Bree A, Verschuren WM, Bjørke-Monsen AL, Van der Put NM, Heil SG, Trijbels FJ, Blom HJ. Effect of the methylenetetrahydrofolate reductase 677C→ T mutation on the relations among folate intake and plasma folate and homocysteine concentrations in a general population sample. The American journal of clinical nutrition. 2003 Mar 1;77(3):687-93.

1026. Lindeman RD, Romero LJ, Yau CL, Koehler KM, Baumgartner RN, Garry PJ. Serum Homocysteine Concentrations and Their Relation to Serum Folate and Vitamin B₁₂ Concentrations and Coronary Artery Disease Prevalence in an Urban, Bi-Ethnic Community. Ethnicity & Disease. 2003 Apr 1;13(2):178-85.

1028. Bjelland I, Tell GS, Vollset SE, Refsum H, Ueland PM. Folate, vitamin B12, homocysteine, and the MTHFR 677C→ T polymorphism in anxiety and depression: the Hordaland Homocysteine Study. Archives of general psychiatry. 2003 Jun 1;60(6):618-26.

1029. Ray JG, Vermeulen MJ, Langman LJ, Boss SC, Cole DE. Persistence of vitamin B12 insufficiency among elderly women after folic acid food fortification. Clinical biochemistry. 2003 Jul 1;36(5):387-91.

1030. Hao L, Ma J, Stampfer MJ, Ren A, Tian Y, Tang YI, Willett WC, Li Z. Geographical, seasonal and gender differences in folate status among Chinese adults. The Journal of nutrition. 2003 Nov 1;133(11):3630-5.

1031. Clarke R, Grimley Evans J, Schneede J, Nexo E, Bates C, Fletcher A, Prentice A, Johnston C, Ueland PM, Refsum H, Sherliker P. Vitamin B12 and folate deficiency in later life. Age and ageing. 2004 Jan 1;33(1):34-41.

1032. Nurk E, Tell GS, Vollset SE, Nygård O, Refsum H, Nilsen RM, Ueland PM. Changes in lifestyle and plasma total homocysteine: the Hordaland Homocysteine Study. The American journal of clinical nutrition. 2004 May 1;79(5):812-9.

1033. Ramos MI, Allen LH, Haan MN, Green R, Miller JW. Plasma folate concentrations are associated with depressive symptoms in elderly Latina women despite folic acid fortification. The American journal of clinical nutrition. 2004 Oct 1;80(4):1024-8.

1036. Gori AM, Corsi AM, Fedi S, Gazzini A, Sofi F, Bartali B, Bandinelli S, Gensini GF, Abbate R, Ferrucci L. A proinflammatory state is associated with hyperhomocysteinemia in the elderly–. The American journal of clinical nutrition. 2005 Aug 1;82(2):335-41.

1037. Chen KJ; Pan WH, Shaw NS, Huang RF, Lin BF. Association between dietary folate-rich food intake and folate status of elderly Taiwanese. Asia Pacific Journal of Clinical Nutrition 2005 (14): 244-249.

1041. Song WO, Chung CE, Chun OK, Cho S. Serum homocysteine concentration of US adults associated with fortified cereal consumption. Journal of the American College of Nutrition. 2005 Dec 1;24(6):503-9.

1042. Fakhrzadeh H, Ghotbi S, Pourebrahim R, Nouri M, Heshmat R, Bandarian F, Shafaee A, Larijani B. Total plasma homocysteine, folate, and vitamin b12 status in healthy Iranian adults: the Tehran homocysteine survey (2003–2004)/a cross–sectional population based study. BMC public health. 2006 Dec;6(1):1-8.

1045. de Lau LM, Refsum H, Smith AD, Johnston C, Breteler MM. Plasma folate concentration and cognitive performance: Rotterdam Scan Study. The American journal of clinical nutrition. 2007 Sep 1;86(3):728-34.

1050. Clarke R, Birks J, Nexo E, Ueland PM, Schneede J, Scott J, Molloy A, Evans JG. Low vitamin B-12 status and risk of cognitive decline in older adults. The American journal of clinical nutrition. 2007 Nov 1;86(5):1384-91.

1053. Clarke R, Sherliker P, Hin H, Molloy AM, Nexo E, Ueland PM, Emmens K, Scott JM, Evans JG. Folate and vitamin B12 status in relation to cognitive impairment and anaemia in the setting of voluntary fortification in the UK. British journal of nutrition. 2008 Nov;100(5):1054-9.

1056. Bakulski KM, Park SK, Weisskopf MG, Tucker KL, Sparrow D, Spiro III A, Vokonas PS, Nie LH, Hu H, Weuve J. Lead exposure, B vitamins, and plasma homocysteine in men 55 years of age and older: the VA normative aging study. Environmental Health Perspectives. 2014 Oct;122(10):1066-74.

1057. Cabrera S, Benavente D, Alvo M, De Pablo P, Ferro CJ. Vitamin B12 deficiency is associated with geographical latitude and solar radiation in the older population. Journal of Photochemistry and Photobiology B: Biology. 2014 Nov 1;140:8-13.

1059. Zhang W, Li Y, Wang TD, Meng HX, Min GW, Fang YL, Niu XY, Ma LS, Guo JH, Zhang J, Sun MZ. Nutritional status of the elderly in rural North China: a cross-sectional study. The journal of nutrition, health & aging. 2014 Oct;18:730-6.

1060. Laillou A, Yakes E, Le TH, Wieringa FT, Le BM, Moench-Pfanner R, Berger J. Intra-individual double burden of overweight and micronutrient deficiencies among Vietnamese women. PLoS One. 2014 Oct 15;9(10):e110499.

1062. Liu XD, Gao B, Sun D, Shi M, Ma YY, Liu ZR, Wang B, Xu X, Xu X, Ji QH, Zhao G. Prevalence of hyperhomocysteinaemia and some of its major determinants in Shaanxi Province, China: a cross-sectional study. British Journal of Nutrition. 2015 Feb;113(4):691-8.

1068. Horvat P, Gardiner J, Kubinova R, Pajak A, Tamosiunas A, Schöttker B, Pikhart H, Peasey A, Jansen E, Bobak M. Serum folate, vitamin B-12 and cognitive function in middle and older age: The HAPIEE study. Experimental gerontology. 2016 Apr 1;76:33-8.

1076. Stiefel F, Zdrojewski C, Hadj FB, Boffa D, Dorogi Y, So A, Ruiz J, De Jonge P. Effects of a multifaceted psychiatric intervention targeted for the complex medically ill: a randomized controlled trial. Psychotherapy and psychosomatics. 2008;77(4):247-56.

1077. Hao L, Yang QH, Li Z, Bailey LB, Zhu JH, Hu DJ, Zhang BL, Erickson JD, Zhang L, Gindler J, Li S. Folate status and homocysteine response to folic acid doses and withdrawal among young Chinese women in a large-scale randomized double-blind trial. The American journal of clinical nutrition. 2008 Aug 1;88(2):448-57.

1081. Gopinath B, Wang JJ, Flood VM, Burlutsky G, Wong TY, Mitchell P. The associations between blood levels of homocysteine, folate, vitamin B12, and retinal vascular caliber. American journal of ophthalmology. 2009 Dec 1;148(6):902-9.

1082. Semmler A, Moskau S, Grigull A, Farmand S, Klockgether T, Smulders Y, Blom H, Zur B, Stoffel-Wagner B, Linnebank M. Plasma folate levels are associated with the lipoprotein profile: a retrospective database analysis. Nutrition Journal. 2010 Dec;9(1):1-4.

1085. Brosnan JT, Mills JL, Ueland PM, Shane B, Fan R, Chiu CY, Pangilinan F, Brody LC, Brosnan ME, Pongnopparat T, Molloy AM. Lifestyle, metabolite, and genetic determinants of formate concentrations in a cross-sectional study in young, healthy adults. The American Journal of Clinical Nutrition. 2018 Mar 1;107(3):345-54.

1086. Saraswathy KN, Joshi S, Yadav S, Garg PR. Metabolic distress in lipid & one carbon metabolic pathway through low vitamin B-12: a population based study from North India. Lipids in health and disease. 2018 Dec;17(1):1-8.

1088. Yang J, Yang A, Wang Z, Wang W, Wang Z, Wang Y, Wang J, Song J, Li L, Lv W, Zhao W. Interactions between serum folate and human papillomavirus with cervical intraepithelial neoplasia risk in a Chinese population-based study. The American journal of clinical nutrition. 2018 Nov 1;108(5):1034-42.

1089. Msemo OA, Bygbjerg IC, Møller SL, Nielsen BB, Ødum L, Perslev K, Lusingu JP, Kavishe RA, Minja DT, Schmiegelow C. Prevalence and risk factors of preconception anemia: A community based cross sectional study of rural women of reproductive age in northeastern Tanzania. PLoS one. 2018 Dec 18;13(12):e0208413.

1091. Gylling B, Myte R, Ulvik A, Ueland PM, Midttun Ø, Schneede J, Hallmans G, Häggström J, Johansson I, Van Guelpen B, Palmqvist R. One‐carbon metabolite ratios as functional B‐vitamin markers and in relation to colorectal cancer risk. International journal of cancer. 2019 Mar 1;144(5):947-56.

1094. Pfeiffer CM, Sternberg MR, Zhang M, Fazili Z, Storandt RJ, Crider KS, Yamini S, Gahche JJ, Juan W, Wang CY, Potischman N. Folate status in the US population 20 y after the introduction of folic acid fortification. The American journal of clinical nutrition. 2019 Nov 1;110(5):1088-97

1097. Chen MY, Rose CE, Qi YP, Williams JL, Yeung LF, Berry RJ, Hao L, Cannon MJ, Crider KS. Defining the plasma folate concentration associated with the red blood cell folate concentration threshold for optimal neural tube defects prevention: a population-based, randomized trial of folic acid supplementation. The American journal of clinical nutrition. 2019 May 1;109(5):1452-61.

1104. Cohen E, Margalit I, Shochat T, Goldberg E, Krause I. Sex Differences in Folate Levels: A Cross Sectional Study of a Large Cohort from Israel. The Israel Medical Association Journal: IMAJ. 2021 Jan 1;23(1):17-22.

1105. Tamura T, Kuriyama N, Koyama T, Ozaki E, Matsui D, Kadomatsu Y, Tsukamoto M, Kubo Y, Okada R, Hishida A, Sasakabe T. Association between plasma levels of homocysteine, folate, and vitamin B12, and dietary folate intake and hypertension in a cross-sectional study. Scientific reports. 2020 Oct 28;10(1):1-9.

1109. Nalder L, Zheng B, Chiandet G, Middleton LT, De Jager CA. Vitamin B12 and folate status in cognitively healthy older adults and associations with cognitive performance. The journal of nutrition, health & aging. 2021 Mar;25(3):287-94.

1112. Jia X, Ren M, Zhang Y, Ye R, Zhang L, Li Z. Association between tea drinking and plasma folate concentration among women aged 18–30 years in China. Public Health Nutrition. 2021 Oct;24(15):4929-36.

1113. Boumenna T, Scott TM, Lee JS, Palacios N, Tucker KL. Folate, vitamin B-12, and cognitive function in the Boston Puerto Rican Health Study. The American Journal of Clinical Nutrition. 2021 Jan;113(1):179-86.

1122. Lee JE, Li H, Giovannucci E, Lee IM, Selhub J, Stampfer M, Ma J. Prospective study of plasma vitamin B6 and risk of colorectal cancer in men. Cancer epidemiology, biomarkers & prevention. 2009 Apr 1;18(4):1197-202.

1127. Ilozumba MN, Cheng TY, Neuhouser ML, Miller JW, Beresford SA, Duggan DJ, Toriola AT, Song X, Zheng Y, Bailey LB, Shadyab AH. Associations between plasma choline metabolites and genetic polymorphisms in one-carbon metabolism in postmenopausal women: The Women's Health Initiative Observational Study. The Journal of nutrition. 2020 Nov 19;150(11):2874-81.

1128. Shen M, Tan H, Zhou S, Retnakaran R, Smith GN, Davidge ST, Trasler J, Walker MC, Wen SW. Serum folate shows an inverse association with blood pressure in a cohort of Chinese women of childbearing age: a cross-sectional study. PLoS One. 2016 May 16;11(5):e0155801.

1129. Han YY, Song JY, Talbott EO. Serum folate and prostate-specific antigen in the United States. Cancer Causes & Control. 2013 Aug;24(8):1595-604.

1139. Lin BF, Lin RF, Yeh WT, Pan WH. The Folate Status in Taiwanese Population. Nutritional Sciences Journal. 1999;24(1):99-17.

1142. Fakhrzadeh H, Ghotbi S, Pourebrahim R, Nouri M, Heshmat R, Bandarian F, Shafaee A, Larijani B. Total plasma homocysteine, folate, and vitamin b12 status in healthy Iranian adults: the Tehran homocysteine survey (2003–2004)/a cross–sectional population based study. BMC public health. 2006 Dec;6(1):1-8.

1157. Van De Rest O, Durga J, Verhoef P, Melse-Boonstra A, Brants HA. Validation of a food frequency questionnaire to assess folate intake of Dutch elderly people. British journal of nutrition. 2007 Nov;98(5):1014-20.

1160. Bates CJ, Mansoor MA, Pentieva KD, Hamer M, Mishra GD. Biochemical risk indices, including plasma homocysteine, that prospectively predict mortality in older British people: the National Diet and Nutrition Survey of People Aged 65 Years and Over. British journal of nutrition. 2010 Sep;104(6):893-9.

1166. Odewole OA, Williamson RS, Zakai NA, Berry RJ, Judd SE, Qi YP, Adedinsewo DA, Oakley Jr GP. Near-elimination of folate-deficiency anemia by mandatory folic acid fortification in older US adults: Reasons for Geographic and Racial Differences in Stroke study 2003–2007. The American journal of clinical nutrition. 2013 Oct 1;98(4):1042-7.

1168. Hopkins SM, Gibney MJ, Nugent AP, McNulty H, Molloy AM, Scott JM, Flynn A, Strain JJ, Ward M, Walton J, McNulty BA. Impact of voluntary fortification and supplement use on dietary intakes and biomarker status of folate and vitamin B-12 in Irish adults. The American journal of clinical nutrition. 2015 Jun 1;101(6):1163-72.

1170. Abuawad A, Spratlen MJ, Parvez F, Slavkovich V, Ilievski V, Lomax-Luu AM, Saxena R, Shahriar H, Uddin MN, Islam T, Graziano JH. Association between body mass index and arsenic methylation in three studies of Bangladeshi adults and adolescents. Environment International. 2021 Apr 1;149:106401.

1175. Castro F, Melgarejo J, Chavez CA, De Erausquin GA, Terwilliger JD, Lee JH, Maestre GE. Total plasma homocysteine and depressive symptoms in older Hispanics. Journal of Alzheimer's Disease. 2021 Jan 1;82(s1):S263-9.

1178. Ndiaye NF, Dossou NI, Sadji M, Diouf A, Badiane A, Lo NB, Guiro AT, Wade S. Folic acid, iron deficiencies and anemia among Senegalese women of childbearing age: national study. Inannals OF Nutrition And Metabolism 2015 (Vol. 67, pp. 164-165).

1184. Laird E, Casey MC, Ward M, Hoey L, Hughes CF, McCarroll K, Cunningham C, Strain JJ, McNulty H, Molloy AM. Dairy intakes in older Irish adults and effects on vitamin micronutrient status: Data from the TUDA study. The journal of nutrition, health & aging. 2017 Nov;21(9):954-61.

1190. Chen KJ, Pan WH, Lin YC, Lin BF. Trends in folate status in the Taiwanese population aged 19 years and older from the Nutrition and Health Survey in Taiwan 1993-1996 to 2005-2008. Asia Pacific Journal of Clinical Nutrition. 2011 Jan;20(2):275-82.

1193. Zhu JH, Hu DJ, Hao L, Zhang BL, Cogswell ME, Bailey LB, Li Z, Berry RJ. Iron, Folate, and B. Int. J. Vitam. Nutr. Res. 2010;80(2):144-54.

1194. Rosenthal J, Lopez-Pazos E, Dowling NF, Pfeiffer CM, Mulinare J, Vellozzi C, Zhang M, Lavoie DJ, Molina R, Ramirez N, Reeve ME. Folate and vitamin B12 deficiency among non-pregnant women of childbearing-age in Guatemala 2009–2010: prevalence and identification of vulnerable populations. Maternal and child health journal. 2015 Oct;19(10):2272-85.

1195. Ma R, Wang L, Jin L, Li Z, Ren A. Plasma folate levels and associated factors in women planning to become pregnant in a population with high prevalence of neural tube defects. Birth Defects Research. 2017 Aug 1;109(13):1039-47.

1197. Ndiaye NF, Idohou-Dossou N, Diouf A, Guiro AT, Wade S. Folate deficiency and anemia among women of reproductive age (15-49 Years) in Senegal: Results of a national cross-sectional survey. Food and nutrition bulletin. 2018 Mar;39(1):65-74.

1198. Wirth JP, Rajabov T, Petry N, Woodruff BA, Shafique NB, Mustafa R, Tyler VQ, Rohner F. Micronutrient deficiencies, over-and undernutrition, and their contribution to anemia in Azerbaijani preschool children and non-pregnant women of reproductive age. Nutrients. 2018 Oct 11;10(10):1483.

1200. Andreev A, Erdinc B, Shivaraj K, Schmutz J, Levochkina O, Bhowmik D, Farag F, Money KM, Primavera LH, Gotlieb V, Sahni S. The association between anemia of chronic inflammation and Alzheimer’s disease and related dementias. Journal of Alzheimer's Disease Reports. 2020 Jan 1;4(1):379-91.

1203. De Vogel S, Meyer K, Fredriksen Å, Ulvik A, Ueland PM, Nygård O, Vollset SE, Tell GS, Tretli S, Bjørge T. Serum folate and vitamin B12 concentrations in relation to prostate cancer risk—a Norwegian population-based nested case–control study of 3000 cases and 3000 controls within the JANUS cohort. International journal of epidemiology. 2013 Feb 1;42(1):201-10.

1208. McNamee T, Hyland T, Harrington J, Cadogan S, Honari B, Perera K, Fitzgerald AP, Perry IJ, Cahill MR. Haematinic deficiency and macrocytosis in middle-aged and older adults. PLoS One. 2013 Nov 7;8(11):e77743.

1209. Gall S, Seal J, Taylor R, Dwyer T, Venn A. Folate status and socio‐demographic predictors of folate status, among a national cohort of women aged 26–36 in Australia, 2004–2006. Australian and New Zealand journal of public health. 2012 Oct;36(5):421-6.

1212. Collin SM, Metcalfe C, Refsum H, Lewis SJ, Zuccolo L, Smith GD, Chen L, Harris R, Davis M, Marsden G, Johnston C. Circulating Folate, Vitamin B12, Homocysteine, Vitamin B12 Transport Proteins, and Risk of Prostate Cancer: a Case-Control Study, Systematic Review, and Meta-analysisFolate, B12, and Prostate Cancer. Cancer epidemiology, biomarkers & prevention. 2010 Jun 1;19(6):1632-42.

1214. Hund L, Northrop-Clewes CA, Nazario R, Suleymanova D, Mirzoyan L, Irisova M, Pagano M, Valadez JJ. A novel approach to evaluating the iron and folate status of women of reproductive age in Uzbekistan after 3 years of flour fortification with micronutrients. PloS one. 2013 Nov 19;8(11):e79726.

1216. Haidar J, Melaku U, Pobocik RS. Folate deficiency in women of reproductive age in nine administrative regions of Ethiopia: an emerging public health problem. South African Journal of Clinical Nutrition. 2010 Jan 1;23(3):132-7.

1220. Finkelstein JL, Fothergill A, Johnson CB, Guetterman HM, Bose B, Jabbar S, Zhang M, Pfeiffer CM, Qi YP, Rose CE, Williams JL. Anemia and vitamin B-12 and folate status in women of reproductive age in southern India: estimating population-based risk of neural tube defects. Current Developments in Nutråition. 2021 May;5(5):nzab069.

1221. Shams M, Homayouni K, Omrani GR. Serum folate and vitamin B [12] status in healthy Iranian adults. EMHJ-Eastern Mediterranean Health Journal, 15 (5), 1285-1292, 2009. 2009.

1222. Yakub M, Iqbal MP. Association of blood lead (Pb) and plasma homocysteine: a cross sectional survey in Karachi, Pakistan. PLoS One. 2010 Jul 21;5(7):e11706.

2001. Hustad S, Midttun Ø, Schneede J, Vollset SE, Grotmol T, Ueland PM. The Methylenetetrahydrofolate Reductase 677C→T Polymorphism as a Modulator of a B Vitamin Network with Major Effects on Homocysteine Metabolism. Am J Hum Genet. 2007 May;80(5):846–55.

2002. Rosenthal J, Largaespada N, Bailey LB, Cannon M, Alverson CJ, Ortiz D, et al. Folate Deficiency Is Prevalent in Women of Childbearing Age in Belize and Is Negatively Affected by Coexisting Vitamin B-12 Deficiency: Belize National Micronutrient Survey 2011. The Journal of nutrition. 2017;147(6):1183–93.

2003. Castillo-Lancellotti C, Margozzini P, Valdivia G, Padilla O, Uauy R, Rozowski J, et al. [Serum folate and vitamin B12 in older people: results from the Chilean National Health Survey 2009-2010]. Revista medica de Chile. 2013;141(9):1107–16.

2004. Ministry of Health. Encuesta Nacional de Nutricion Costa Rica 2008-2009. [Internet]. San Jose, Costa Rica; 2012. Available from: http://www.ministeriodesalud.go.cr/index.php/centro-de-informacion/material-publicado

2005. Ecuador National Health Survey 2012 [Internet]. Ecuador: Ministry of public health; 2014 [cited 2023 Jan 26]. Available from: https://www.ecuadorencifras.gob.ec/documentos/web-inec/Estadisticas_Sociales/ENSANUT/MSP_ENSANUT-ECU_06-10-2014.pdf

2006. Report on the micronutrient status of women in Fiji: results of the 2004 National Nutrition Survey [Internet]. Fiji: Government of Fiji; 2004. Available from: http://www.nutrition.gov.fj/wp-content/uploads/2013/02/2004.FIJI-NNS-MAIN-REPORT_11.04.13.pdf

2007. Labadarios D. Executive summary of the National Food Consumption Survey Fortification Baseline (NFCS-FB-1) South Africa, 2005 – ScienceOpen. S Afr J Clin Nutr. 2005;21:245–300.

2008. National Nutrition Survey in Tajikistan [Internet]. Ministry of Health and Social Protection of hte Reupublic of Tajikistan; 2016 [cited 2023 Jan 26]. Available from: https://www.unicef.org/tajikistan/media/491/file/National%20Nutrition%20Survey%20in%20Tajikistan%202016%20.pdf

2009. Phillipines 8th National Nutrition Survey [Internet]. Phillipines: Department of Science and Technolog; 2013 [cited 2023 Jan 26]. Available from: https://www.fnri.dost.gov.ph/index.php/nutrition-statistic/19-nutrition-statistic/118-8th-national-nutrition-survey

2010. Castetbon K, Vernay M, Malon A, Salanave B, Deschamps V, Roudier C, et al. Dietary intake, physical activity and nutritional status in adults: the French nutrition and health survey (ENNS, 2006–2007). British Journal of Nutrition. 2009 Sep;102(5):733–43.

2011. Parnell W, New Zealand, Ministry of Health. Methodology report for the 2008/09 New Zealand adult nutrition survey [Internet]. Wellington, N.Z.: Ministry of Health; 2011 [cited 2021 Oct 1]. Available from: http://www.moh.govt.nz/moh.nsf/pagesmh/10807/$File/methodology-report.pdf

2012. Encuesta Nacional de Salud y Nutricion [Internet]. Mexico; 2012 [cited 2023 Jan 26]. Available from: https://ensanut.insp.mx/encuestas/ensanut2012/doctos/informes/ENSANUT2012ResultadosNacionales.pdf

2013. Rohner F, Northrop-Clewes C, Tschannen AB, Bosso PE, Kouassi-Gohou V, Erhardt JG, et al. Prevalence and public health relevance of micronutrient deficiencies and undernutrition in pre-school children and women of reproductive age in Côte d’Ivoire, West Africa. Public Health Nutrition. 2014 Sep;17(9):2016–28.

2014. Folsäureversorgung von Frauen im gebärfähigen Alte [Internet]. Germany: Thieme Group; Available from: https://www.thieme.de/statics/dokumente/thieme/final/de/dokumente/zw_das-gesundheitswesen/gesu-suppl_klein.pdf#page=153

2016. Australian Health Survey: Biomedical Results for Nutrients, 2011-12 | Australian Bureau of Statistics [Internet]. [cited 2023 Jan 26]. Available from: https://www.abs.gov.au/statistics/health/health-conditions-and-risks/australian-health-survey-biomedical-results-nutrients/latest-release

2017. Pfeiffer CM, Hughes JP, Lacher DA, Bailey RL, Berry RJ, Zhang M, et al. Estimation of Trends in Serum and RBC Folate in the U.S. Population from Pre- to Postfortification Using Assay-Adjusted Data from the NHANES 1988–2010123. J Nutr. 2012 May;142(5):886–93.

2018. Gebremichael B, Roba HS, Getachew A, Tesfaye D, Asmerom H (2023) Folate deficiency among women of reproductive age in Ethiopia: A systematic review and meta-analysis. PLoS ONE 18(5): e0285281. [https://doi.org/10.1371/journal. pone.0285281](https://doi.org/10.1371/journal.%20pone.0285281)

2019. Jiang Y, Cao H, Chen X, Yu G, Song C, Duan H, Tian F, Wan H and Shen J (2022) Associations of serum folate and vitamin C levels with metabolic dysfunction-associated fatty liver disease in US adults: A nationwide cross-sectional study. Front. Public Health 10:1022928. doi: 10.3389/fpubh.2022.1022928

2020. Xu, L, Wu, QX, Li, X, Fang YJ, Zhou RL, Che MM, Ma T, Zhang CX, Serum flavin mononucleotide but not riboflavin is inversely associated with the risk of colorectal cancer. Food Funct, 2022,13, 12246-12257

2021. Lee, M.-R.; Jung, S.M. Serum Folate Related to Five Measurements of Obesity and High-Sensitivity C-Reactive Protein in Korean Adults. Nutrients 2022, 14, 3461. https://doi.org/10.3390/ nu14173461

2022. Chung M, Hsu H, Mao Y, Wu C, Ho C, Liu C Chung C, Association and mediation analyses among multiple metals exposure, plasma folate, and community-based impaired estimated glomerular filtration rate in central Taiwan Environmental Health (2022) 21:44 <https://doi.org/10.1186/s12940-022-00855-x>

2023. Cheng M, Xue H, Li, X, Yan Q, Zhu D, Wang Y, shi Y, Fu C Prevalence of hyperhomocysteinemia (HHcy) and its major determinants among hypertensive patients over 35 years of age. European Journal of Clinical Nutrition (2022) 76:616–623; <https://doi.org/10.1038/s41430-021-00983-6>

2024. Du Y, X, Zhang J, Zhang L, Liu j, Plasma folate deficiency increases the risk for abnormal blood pressure in Chinese women of childbearing age. doi.org/10.1016/j.nutres.2021.12.003

2025. Jingzhu Fu, Qian Liu, Meilin Zhang, Changqing Sun, Yue Du, Yun Zhu, Hongyan Lin, Mengdi Jin, Fei Ma, Wen Li, Huan Liu, Jing Yan, Yongjie Chen, Guangshun Wang & Guowei Huang (2022) Association between methionine cycle metabolite-related diets and mild cognitive impairment in older Chinese adults: a population-based observational study, Nutritional Neuroscience, 25:7, 1495-1508, DOI: [10.1080/1028415X.2021.1872959](https://doi.org/10.1080/1028415X.2021.1872959)

2026. Lan, K.-M.; Wang, L.-K.; Lin, Y.-T.; Hung, K.-C.; Wu, L.-C.; Ho, C.-H.; Chang, C.-Y.; Chen, J.-Y. Suboptimal Plasma Vitamin C Is Associated with Lower Bone Mineral Density in Young and Early Middle-Aged Men: A Retrospective Cross-Sectional Study. Nutrients 2022, 14, 3556. https://doi.org/10.3390/ nu14173556

| 3002. | Gebremichael B, Roba HS, Getachew A, Tesfaye D, Asmerom H. Folate deficiency among women of reproductive age in Ethiopia: A systematic review and meta-analysis. PLoS One.2023;18(5):e0285281. |
| --- | --- |
| 3008. | Jiang Y, Cao H, Chen X, Yu G, Song C, Duan H, et al. Associations of serum folate and vitamin C levels with metabolic dysfunction-associated fatty liver disease in US adults: A nationwide cross-sectional study. Front Public Health. 2022 Oct 26;10:1022928. |
| 3009. | Xu L, Wu QX, Li X, Fang YJ, Zhou RL, Che MM, et al. Serum flavin mononucleotide but not riboflavin is inversely associated with the risk of colorectal cancer. Food Funct. 2022 Nov 28;13(23):12246–57. |
| 3011. | Lee MR, Jung SM. Serum Folate Related to Five Measurements of Obesity and High-Sensitivity C-Reactive Protein in Korean Adults. Nutrients. 2022 Aug 24;14(17):3461. |
| 3014. | Chung MC, Hsu HT, Mao YC, Wu CC, Ho CT, Liu CS, et al. Association and mediation analyses among multiple metals exposure, plasma folate, and community-based impaired estimated glomerular filtration rate in central Taiwan. Environmental Health. 2022 Apr 23;21(1):44. |
| 3017. | Cheng M, Xue H, Li X, Yan Q, Zhu D, Wang Y, et al. Prevalence of hyperhomocysteinemia (HHcy) and its major determinants among hypertensive patients over 35 years of age. Eur J Clin Nutr. 2022 Apr;76(4):616–23. |
| 3019. | Du Y, Xia S, Zhang J, Zhang L, Liu J. Plasma folate deficiency increases the risk for abnormal blood pressure in Chinese women of childbearing age. Nutrition Research. 2022 Feb 1;98:9–17. |
| 3021. | Fu J, Liu Q, Zhang M, Sun C, Du Y, Zhu Y, et al. Association between methionine cycle metabolite-related diets and mild cognitive impairment in older Chinese adults: a population-based observational study. Nutritional Neuroscience. 2022 Jul 3;25(7):1495–508. |
| 3026. | Lan KM, Wang LK, Lin YT, Hung KC, Wu LC, Ho CH, et al. Suboptimal Plasma Vitamin C Is Associated with Lower Bone Mineral Density in Young and Early Middle-Aged Men: A Retrospective Cross-Sectional Study. Nutrients. 2022 Aug 29;14(17):3556. |

# References S2: References for studies of NTD prevalence by country, calendar year and fortification types

1 Houcher B, Akar N, Begag S, Egin Y. Neural tube defects in Algeria. INTECH Open Access Publisher; 2012.

2 Njamnshi AK, Djientcheu VDP, Lekoubou A, Guemse M, Obama MT, Mbu R, et al. Neural tube defects are rare among black Americans but not in sub-Saharan black Africans: the case of Yaounde—Cameroon. Journal of the Neurological Sciences. 2008; 270: 13–17. pmid:18295800

3 Ahuka OL, Toko RM, Omanga FU, Tshimpanga BJ. Congenital malformations in the North-Eastern Democratic Republic of Congo during civil war. East African Medical Journal. 2006; 8: 95–99.

4 Anyebuno M, Amofa G, Peprah S, Affram A. Neural tube defects at Korle Bu Teaching Hospital, Accra, Ghana. East African Medical Journal. 1993; 70: 572–574. pmid:8181439

5 Msamati BC, Igbigbi PS, Chisi JE. The incidence of cleft lip, cleft palate, hydrocephalus and spina bifida at Queen Elizabeth Central Hospital, Blantyre, Malawi. The Central African Journal of Medicine. 2000; 46: 292–296. pmid:12002118

6 Ekanem TB, Okon DE, Akpantah AO, Mesembe OE, Eluwa MA, Ekong MB. Prevalence of congenital malformations in Cross River and Akwa Ibom states of Nigeria from 1980–2003. Congenital Anomalies. 2008; 48: 167–170. pmid:18983583

7 Airede KI. Neural tube defects in the middle belt of Nigeria. Journal of Tropical Pediatrics. 1992; 38: 27–30. pmid:1573689

8 Sayed AR, Bourne D, Pattinson R, Nixon J, Henderson B. Decline in the prevalence of neural tube defects following folic acid fortification and its cost-benefit in South Africa. Birth Defects Research Part A: Clinical and Molecular Teratology. 2008; 82: 211–216.

9 Venter PA, Christianson AL, Hutamo CM, Makhura MP, Gericke GS. Congenital anomalies in rural black South African neonates—a silent epidemic?. South African Medical Journal. 1995; 85: 15–20. pmid:7784908

10 Viljoen DL, Buccimazza S, Dunne T, Molteno C. The prevalence and prevention of neural tube defects in Cape Town. South African Medical Journal. 1995; 85: 630–632. pmid:7482075

11 Kinasha AD, Manji K. The incidence and pattern of neural tube defects in Dar es Salaam, Tanzania. European Journal of Pediatric Surgery. Supplement, 2002; S38–S39.

12 Mohammed YA, Shawky RM, Soliman AA, Ahmed MM. Chromosomal study in newborn infants with congenital anomalies in Assiut University hospital: Cross-sectional study. Egyptian Journal of Medical Human Genetics. 2011; 12: 79–90.

13 Ebrahimi S, Ashkani S, Bagheri F. Prevalence of neural tube defects in Yasuj, Southwest Iran. Shiraz E-Medical Journal. 2013; 14: 1–9.

14 Behrooz AG, Gorjizadeh MH. Prevalence and Correlates of Neural Tube Defect in South West Iran. Sultan Qaboos University Medical Journal. 2007; 7: 31–34. pmid:21654942

15 Abdollahi Z, Elmadfa I, Djazayery A, Golalipour MJ, Sadighi J, Salehi F, et al. Efficacy of flour fortification with folic acid in women of childbearing age in Iran. Annals of Nutrition and Metabolism. 2011; 58: 188–196. pmid:21757892

16 Delshad S, Tabar AK, Samae H, Mollaean M, Nasiri SJ, Jazayeri SM, et al. The incidence of selected congenital malformations during a two-year period in Tehran, Iran. Tropical Doctor. 2009; 39; 156–158. pmid:19535752

17 Afshar M, Golalipour MJ, Farhud D. Epidemiologic aspects of neural tube defects in South East Iran. Neurosciences. 2006; 11: 289–292. pmid:22266439

18 Rad IA, Farrokh-Islamlou HR, Khoshkalam M. Neural tube defects prevalence in a hospital-based study in Urmia. Iranian Journal of Child Neurology. 2008; 2: 19–23.

19 Farhud DD, Hadavi V, Sadighi H. Epidemiology of neural tube defects in the world and Iran. Iranian journal of Public Health. 2000; 29: 83–90.

20 International Clearinghouse for Birth Defects Surveillance and Research. Annual report 2011 with data for 2009. Rome, Italy: The Centre of the International Clearinghouse for Birth Defects Surveillance and Research. 2011.

21 Al-Ani ZR, Al-Haj S, Al-Ani MM, Al-Dulaimy KM, Al-Maraie AK, Al-Ubaida BK. Incidence, types, geographical distribution, and risk factors of congenital anomalies in Al-Ramadi Maternity and Children’s Teaching Hospital, Western Iraq. Saudi Medical Journal. 2012; 33: 979–989. pmid:22964810

22 Al-Sadoon I, Hassan GG, Yacoub A. Depleted Uranium and health of people in Basrah: Epidemiological evidence: Incidence and pattern of congenital anomalies among birth in Basrah during the period 1990–1998. Medical Journal of Basrah University: 1999; 17: 27–33.

23 Amarin ZO, Obeidat AZ. Effect of folic acid fortification on the incidence of neural tube defects. Paediatric and Perinatal Epidemiology. 2010; 24; 349–351. pmid:20618724

24 Aqrabawi HE. Incidence of neural tube defects among neonates at King Hussein Medical Centre, Jordan. Eastern Mediterranean Health Journal. 2005; 11: 819–823. pmid:16700398

25 Masri AT. Neural tube defects in Jordan: A hospital based study. Journal of Pediatric Neurology. 2006; 4: 245–249.

26 Daoud AS, Al-Kaysi F, El-Shanti H, Batieha A, Obeidat A, Al-Sheyyab M. Neural tube defects in northern Jordan. Saudi Medical Journal. 1996; 17: 78–81.

27 Madi SA, Al-Naggar RL, Al-Awadi SA, Bastaki LA. Profile of major congenital malformations in neonates in Al-Jahra region of Kuwait. Eastern Mediterranean Health Journal. 2005; 11: 700–706. pmid:16700386

28 Singh R, Al-Sudani O. Major congenital anomalies at birth in Benghazi, Libyan Arab Jamahiriya, 1995. Eastern Mediterranean Health Journal. 2000; 6: 65–75. pmid:11370343

29 Alasfoor D, ElSayed MK. Fortification of Flour and Outcomes: Oman’s Perspective—Contextual Considerations and Outcome. In: Handbook of Food Fortification and Health 2013 (pp. 321–334). Springer New York.

30 Khattak ST, Khan M, Naheed T, Khattak I, Ismail M. Prevalence and management of anencephaly at Saidu Teaching Hospital, Swat. Journal of Ayub Medical College, Abbottabad. 2009; 22: 61–63.

31 Qazi G. Relationship of selected prenatal factors to pregnancy outcome and congenital anomalies. Journal of Ayub Medical College, Abbottabad. 2010; 22: 41–45. pmid:22455258

32 Perveen F, Tyyab S. Frequency and pattern of distribution of congenital anomalies in the newborn and associated maternal risk factors. Journal of the College of Physicians and Surgeons—Pakistan. 2007; 17: 340–343. pmid:17623582

33 Najmi RS. Risk factors, clinical presentation and perinatal outcome of congenital malformations in a hospital based study. Journal of the College of Physicians and Surgeons—Pakistan. 1998; 8: 265–269.

34 Jooma R. Preventing neural tube defects by folic acid fortification of flour. The Journal of the Pakistan Medical Association. 2004; 54: 540–541. pmid:15623176

35 Bener A, Kurdi B, Hoffmann GF, Caksen H, Teebi A. The pattern of neural tube defects in a highly endogamous society: A 25-year incidence trends. Journal of Pediatric Neurology. 2012; 10: 193–198.

36 Al-Jama F. Congenital malformations in newborns in a teaching hospital in eastern Saudi Arabia. Journal of Obstetrics & Gynecology. 2001; 21: 595–598.

37 Asindi A, Al-Shehri A. Neural tube defects in the Asir region of Saudi Arabia. Annals of Saudi Medicine. 2001; 21: 26–29. pmid:17264584

38 Safdar OY, Al-Dabbagh AA, AbuElieneen WA, Kari JA. Decline in the incidence of neural tube defects after the national fortification of flour (1997–2005). Saudi Medical Journal. 2007; 28: 1227–1229. pmid:17676207

39 Murshid WR. Spina bifida in Saudi Arabia: is consanguinity among the parents a risk factor?. Pediatric Neurosurgery. 2000; 32: 10–12. pmid:10765132

40 Hakami WS, Majeed-Saidan MA. The incidence and spectrum of central nervous system malformations in newborns over a decade (2001–2010) in the Central Region of Saudi Arabia. Saudi Medical Journal. 2011; 32: 1137–1142. pmid:22057601

41 Elsheikh GEA, Ibrahim SA. Neural tube defects in Omdurman Maternity Hospital, Sudan. Khatoum Medical Journal. 2009; 2: 185–190.

42 Al Hosani H, Salah M, Abu-Zeid H, Farag HM, Saade D. The national congenital anomalies register in the United Arab Emirates. East Mediterranean Health Journal. 2005; 11: 690–699.

43 European Surveillance of Congenital Anomalies (EUROCAT). Prevalence Tables; 2012. Available: http://www.eurocat-network.eu/accessprevalencedata/prevalencetables.

44 Kovacheva K, Simeonova M, Velkova A. Trends and causes of congenital anomalies in the Pleven region, Bulgaria. Balkan Journal of Medical Genetics. 2009; 12: 37–43.

45 Pasternak B, Svanström H, Mølgaard-Nielsen D, Melbye M, Hviid A. Metoclopramide in pregnancy and risk of major congenital malformations and fetal death. The Journal of the American Medical Association. 2013; 310: 1601–1611. pmid:24129464

46 Stoll C, Dott B, Alembik Y, Roth MP. Associated malformations among infants with neural tube defects. American Journal of Medical Genetics Part A. 2011; 155: 565–568.

47 Klusmann A. Heinrich B, Stöpler H, Gärtner J, Mayatepek E, Kries R. A decreasing rate of neural tube defects following the recommendations for periconceptional folic acid supplementation. Acta Paediatrica. 2005; 94: 1538–1542. pmid:16303691

48 McDonnell R, Delany V, Mahony MT, Mullaney C, Lee B, Turner MJ. Neural tube defects in the Republic of Ireland in 2009–11. Journal of Public Health. 2014; 1–7.

49 Zlotogora J, Amitai Y, Leventhal A. Surveillance of neural tube defects in Israel: the effect of the recommendation for periconceptional folic acid. Israel Medical Association Journal. 2006; 8: 601–604. pmid:17058407

50 Petrova JG, Vaktskjold A. The incidence of neural tube defects in Norway and the Arkhangelskaja Oblast in Russia and the association with maternal age. Acta Obstet Gynecol Scand. 2009; 88: 667–672. pmid:19353336

51 Poretti A, Anheier T, Zimmermann R, Boltshausei E. Neural tube defects in Switzerland from 2001 to 2007: are periconceptual folic acid recommendations being followed?. Swiss Medical Weekly. 2008; 138: 608–613. pmid:18941947

52 Onrat ST, Seyman H, Konuk M. Incidence of neural tube defects in Afyonkarahisar, Western Turkey. Genetics and Molecular Research. 2009; 8: 154–161. pmid:19283682

53 Mandıracıoğlu A, Ulman I, Lüleci E, Ulman C. The incidence and risk factors of neural tube defects in İzmir, Turkey: A nested case-control study. The Turkish Journal of Pediatrics. 2004; 46: 214–220. pmid:15503473

54 Tuncbilek E, Boduroğlu K, Alikaşifoğlu M. Neural tube defects in Turkey: prevalence, distribution and risk factors. The Turkish Journal of Pediatrics. 1998; 41: 299–305.

55 Himmetoglu O, Tiras MB, Gursoy R, Karabacak O, Sahin I, Onan A. The incidence of congenital malformations in a Turkish population. International Journal of Gynecology & Obstetrics. 1996; 55: 117–121.

56 Groisman B, Bidondo MP, Barbero P, Gili JA, Liascovich R. RENAC: Registro Nacional de Anomalías Congénitas de Argentina. Archivos Argentinos de Pediatría. 2013; 111: 484–494. pmid:24196761

57 López‐Camelo JS, Castilla EE, Orioli IM. Folic acid flour fortification: impact on the frequencies of 52 congenital anomaly types in three South American countries. American Journal of Medical Genetics Part A. 2010; 152: 2444–2458.

57 López‐Camelo JS, Castilla EE, Orioli IM. Folic acid flour fortification: impact on the frequencies of 52 congenital anomaly types in three South American countries. American Journal of Medical Genetics Part A. 2010; 152: 2444–2458.

57 López‐Camelo JS, Castilla EE, Orioli IM. Folic acid flour fortification: impact on the frequencies of 52 congenital anomaly types in three South American countries. American Journal of Medical Genetics Part A. 2010; 152: 2444–2458.

58 Orioli IM, Lima do Nascimento R, López‐Camelo JS, Castilla EE. Effects of folic acid fortification on spina bifida prevalence in Brazil. Birth Defects Research Part A: Clinical and Molecular Teratology. 2011; 91: 831–835

59 Nazer J, Cifuentes L. Resultados del Programa de Prevención de Defectos de Tubo Neural en Chile mediante la fortificación de la harina con ácido fólico: Período 2001–2010. Revista Médica de Chile. 2013; 141: 751–757.

60 Pachajoa H, Ariza Y, Isaza C, Méndez F. Defectos congénitos mayores en un hospital de tercer nivel en Cali, Colombia. Revista de Salud Pública. 2011; 13: 152–162.

61 Zarante I, Franco L, López C, Fernández N. Frecuencia de malformaciones congénitas: evaluación y pronóstico de 52.744 nacimientos en tres ciudades colombianas. Biomédica. 2010; 30: 65–71.

62 Ostos H, Astaiza G, Garcia F, Bautista M, Rojas F, Bermúdez A. Disminución de la incidencia de defectos de cierre del tubo neural en el Hospital Universitario de Neiva: posible efecto de la promoción del consumo de ácido fólico. Biomédica. 2000; 20: 18–24.

63 de la Paz Barboza-Argüello M, Umaña-Solís LM, Azofeifa A, Valencia D, Flores AL, Rodríguez-Aguilar S, et al. Neural Tube Defects in Costa Rica, 1987–2012: origins and Development of Birth Defect Surveillance and Folic Acid Fortification. Maternal and child Health Journal. 2015; 19: 583–590. pmid:24952876

64 González-Andrade F, López-Pulles R. Congenital malformations in Ecuadorian children: urgent need to create a National Registry of Birth Defects. The Application of Clinical Genetics. 2010; 3: 29–39. pmid:23776361

65 Acevedo CR, Anzueto ER, Mendez AG, Ramirez FM. Prevalencia de anomalías congénitas mayores externas, en recién nacidos atendidos en hospitales nacionales y regionales de Guatemala 2001–2003. Undergraduate. Thesis, School of Medicine, Universidad de San Carlos de Guatemala. 2004.

66 Hernández R, Alvarenga R. Frecuencia de malformaciones congenitas externas en recién nacidos de la Unidad Materno Inantil del Hospital Escuela. Factores de Riesgo. Rev Med Post INAH. 2001; 6: 148–153.

67 Hernandez-Herrera RJ, Alcala-Galvan LG, Flores-Santos R. Prevalencia de defectos del tubo neural en 248 352 nacimientos consecutivos. Revista Medica del Instituto Mexicano Seguro Social. 2008; 46: 201–204.

68 Alfaro N, de Jesús Pérez J, Figueroa I, González Y. Malformaciones congénitas externas en la zona metropolitana de Guadalajara. 10 años de estudio. Investigación en Salud, 2004; 6: 180–187.

69 Navarrete Hernández E, Canún Serrano S, Reyes Pablo AE, del Carmen Sierra Romero M, Valdés Hernández J. Prevalencia de malformaciones congénitas registradas en el certificado de nacimiento y de muerte fetal: México, 2009–2010. Boletín médico del Hospital Infantil de México. 2013; 70: 499–505.

70 Sanabria Rojas HA, Tarqui-Mamani CB, Arias Pachas J, Lam Figueroa NM. Impacto de la fortificación de la harina de trigo con ácido fólico en los defectos del tubo neural, en Lima, Perú. In Anales de la Facultad de Medicina. 2013; 74:175–180.

71 Castilla EE, Orioli IM, Lopez‐Camelo JS, da Graça Dutra M, Nazer‐Herrera J. Preliminary data on changes in neural tube defect prevalence rates after folic acid fortification in South America. America89.Castilla EE, Orioli IM, Lopez‐Camelo JS, da Graça Dutra M, Nazer‐Herrera J. Preliminary data on changes in neural tube defect prevalence rates after folic acid fortification in South America. American Journal of Medical Genetics Part A. 2003; 123: 123–128.n Journal of Medical Genetics Part A. 2003; 123: 123–128.

72 Canfield MA, Mai CT, Wang P, O’Halloran A, Marengo LK, Olney RS, et al. The association between race/ethnicity and major birth defects in the United States, 1999–2007. American Journal of Public Health. 2014; e1–e10.

73 Dey AC, Shahidullah M, Mannan MA, Noor MK, Saha L, Rahman SA. Maternal and neonatal serum zinc level and its relationship with neural tube defects. Journal of Health, Population, and Nutrition. 2010; 28: 343–350. pmid:20824977

74 Sarkar S, Patra C, Dasgupta MK, Nayek K, Karmakar PR. Prevalence of congenital anomalies in neonates and associated risk factors in a tertiary care hospital in eastern India. Journal of Clinical Neonatology. 2013; 2: 131–134. pmid:24251257

75 Sood M, Agarwal N, Verma S, Bhargava SK. Neural tubal defects in an east Delhi hospital. The Indian Journal of Pediatrics. 1991; 58: 363–365. pmid:1937649

76 Sharma AK, Upreti M, Kamboj M, Mehra P, Das K, Misra A, et al. Incidence of neural tube defects at Lucknow over a 10 year period from 1982–1991. Indian Journal of Medical Research. 1994; 99: 223–226. pmid:7927580

77 Mahadevan B, Bhat BV. Neural tube defects in Pondicherry. The Indian Journal of Pediatrics. 2005; 72: 557–559. pmid:16077237

78 Duttachoudhury A, Pal SK. Congenital abnormalities in Durgapur Steel Plant Hospital with special reference to neural tube defect. Journal of the Indian Medical Association. 1997; 95: 135–141. pmid:9357258

79 Ponne S, Lakshmi UK. Incidence of neural tube defects in Erode town. The Indian Journal of Nutrition and Dietetics. 2006; 43: 272–281.

80 Grover N. Congenital malformations in Shimla. The Indian Journal of Pediatrics. 2000; 6: 249–251.

81 Taksande A, Vilhekar K, Chaturvedi P, Jain M. Congenital malformations at birth in Central India: A rural medical college hospital based data. Indian Journal of Human Genetics. 2010; 16: 159–163. pmid:21206705

82 Malla BK. One year review study of congenital anatomical malformation at birth in Maternity Hospital (Prasutigriha), Thapathali, Kathmandu. Kathmandu University Medical Journal. 2007; 5: 557–560.

83 Jaruratanasirikul S, Kor-anantakul O, Limpitikul W, Dissaneevate P, Khunnarakpong N, Sattapanyo A. Prevalence of neural tube defect in southern Thailand: a population-based survey during 2009–2012. Child's Nervous System. 2014; 30: 1269–1275. pmid:24740441

84 Kitisomprayoonkul N, Tongsong T. Neural tube defects: a different pattern in northern Thai population. Journal of the Medical Association of Thailand. 2001; 84: 483–488. pmid:11460957

85 Wasant P, Sathienkijkanchai A. Neural tube defects at Siriraj Hospital, Bangkok, Thailand—10 years review (1990–1999). Journal of the Medical Association of Thailand. 2005; 88; S92–S99.

86 Flood L, Scheil W, Nguyen AM, Sage L, Scott J. An increase in neural tube defect notifications, South Australia, 2009–2010. Western Pacific Surveillance and Response Journal. 2013; 4: 30–39. pmid:24015369

87 Macaldowie A, Hilder L. Neural tube defects in Australia: prevalence before mandatory folic acid fortification. 2011. Canberra, Australia: Australian Institute of Health and Welfare.

88 Fan L, Gong T, Cao X, Du Y. Epidemiologic characteristics of birth defects in the Hainan Province from 2000 to 2010, China. Birth Defects Research Part A: Clinical and Molecular Teratology. 2013; 97: 750–754.

89 Yang M, Zhang S, Du Y. Epidemiology characteristics of birth defects in Shenzhen city during 2003 to 2009, China. The Journal of Maternal-Fetal & Neonatal Medicine. 2015; 28: 799–803.

90 Li X, Zhu J, Wang Y, Mu D, Dai L, Zhou G, et al. Geographic and urban—rural disparities in the total prevalence of neural tube defects and their subtypes during 2006–2008 in China: a study using the hospital-based birth defects surveillance system. BMC Public Health. 2013; 13: 161. pmid:23433029

91 Zhang X, Li S, Wu S, Hao X, Guo S, Suzuki K, et al. Prevalence of birth defects and risk-factor analysis from a population-based survey in Inner Mongolia, China. BMC Pediatrics. 2012; 12: 125. pmid:22900612

92 Dai L, Zhu J, Liang J, Wang YP, Wang H, Mao M. Birth defects surveillance in China. World Journal of Pediatrics. 2011; 7: 302–310. pmid:22015723

93 Zhang XH, Qiu LQ, Huang JP. Risk of birth defects increased in multiple births. Birth Defects Research Part A: Clinical and Molecular Teratology. 2011; 91: 34–38.

94 Chen G, Pei LJ, Huang J, Song XM, Lin LM, Gu X, et al. Unusual patterns of neural tube defects in a high risk region of northern China. Biomedical and Environmental Sciences. 2009; 22: 340–344. pmid:19950530

95 Li Y, Liu XH, Wang FY, Zhao XL, Zhang X, Zhang YP. Analysis of the birth defects among 61 272 live born infants in Beijing. Journal of Peking University, Health Sciences. 2009; 41: 414–417.

96 Liu J, Yang GZ, Zhou JL, Cao SP, Chau DH, Kung HF, et al. Prevalence of neural tube defects in economically and socially deprived area of China. Child's Nervous System. 2007; 23: 1119–1124. pmid:17450368

97 Cheng N, Bai Y, Hu X, Pei H, Li Y, Zhang W, et al. A base-line survey on birth defects in Gansu province, West China. Annals of Tropical Paediatrics: International Child Health. 2003; 23: 25–29.

98 Chen BY, Hwang BF, Guo YL. Epidemiology of congenital anomalies in a population-based birth registry in Taiwan, 2002. Journal of the Formosan Medical Association. 2009; 108: 460–468. pmid:19515626

99 Imaizumi Y, Yamamura H, Nishikawa M, Matsuoka M, Moriyama I. The prevalence at birth of congenital malformations at a maternity hospital in Osaka City, 1948–1990. Japanese Journal of Human Genetics, 1991; 36: 275–287. pmid:1753441

100 Seto T, Nakagawa H, Morikawa Y, Nishijo M, Miura K, Kadoshima Y. Trend of congenital anomalies over 20 years ascertained by population-based monitoring in Ishikawa Prefecture, Japan. Congenital Anomalies. 2003; 43: 286–293. pmid:15041780

101 Kim MA, Yee NH, Choi JS, Choi JY, Seo K. Prevalence of birth defects in Korean livebirths, 2005–2006. Journal of Korean Medical Science. 2012; 27: 1233–1240. pmid:23091323

102 Boo NY, Cheah IG, Thong MK. Neural tube defects in Malaysia: data from the Malaysian National Neonatal Registry. Journal of Tropical Pediatrics. 2013; 59: 338–342. pmid:23583959

103 Dryden R. Birth defects recognized in 10,000 babies born consecutively in Port Moresby General Hospital, Papua New Guinea. Papua New Guinea Medical Journal. 1997; 40: 4–13. pmid:10365565

104 Shi LM, Chia SE, Chan OY, Chew SK, Foong BH. Prevalence of birth defects and parental work in Singapore live births from 1994 to 1998: a population-based study. Occupational Medicine. 2002; 52: 325–331. pmid:12361994

105 Hoang T, Nguyen PVN, Tran DA, Gillerot Y, Reding R, Robert A. External birth defects in southern Vietnam: a population-based study at the grassroots level of health care in Binh Thuan province. BMC Pediatrics. 2013; 13: 67. pmid:23631673

106 Dudin A. Neural tube defect among Palestinians: a hospital-based study. Annals of Tropical Paediatrics. 1997; 17: 217–222. pmid:9425376

# References S3: References for individual trials included in a meta-analysis of folic acid trials for prevention of stroke

1. Toole JF, Malinow MR, Chambless LE, Spence JD, Pettigrew LC, Howard VJ, Sides EG, Wang CH, Stampfer M. Lowering homocysteine in patients with ischemic stroke to prevent recurrent stroke, myocardial infarction, and death: the Vitamin Intervention for Stroke Prevention (VISP) randomized controlled trial. JAMA, 2004; 291:565–575.

2. Jamison RL, Hartigan P, Kaufman JS, Goldfarb DS, Warren SR, Guarino PD, Gaziano JM. Effect of homocysteine lowering on mortality and vascular disease in advanced chronic kidney disease and end-stage renal disease: a randomized controlled trial. JAMA 2007, 298: 1163-70

3. Albert CM, Cook NR, Gaziano JM, Zaharris E, MacFadyen J, Danielson E, Buring JE, Manson JE. Effect of folic acid and B vitamins on risk of cardiovascular events and total mortality among women at high risk for cardiovascular disease: a randomized trial. JAMA. 2008; 299:2027–2036.

4. Lonn E, Yusuf S, Arnold MJ, Sheridan P, Pogue J, Micks M, McQueen MJ, Probstfield J, Fodor G, Held C, Genest J. Homocysteine lowering with folic acid and B vitamins in vascular disease. N Engl J Med, 2006; 354:1567–1577.

5. VITATOPS Trial Study Group. B vitamins in patients with recent transient ischaemic attack or stroke in the VITAmins TO Prevent Stroke (VITATOPS) trial: a randomised, double-blind, parallel, placebo-controlled trial. Lancet Neurology, 2010 1;9(9):855- 65.18705976.

6. Bostom AG, Carpenter MA, Hunsicker L, Jacques PF, Kusek JW, Levey AS, McKenney JL, Mercier RY, Pfeffer MA, Selhub J, FAVORIT Study Investigators. Baseline characteristics of participants in the Folic Acid for Vascular Outcome Reduction in Transplantation (FAVORIT) trial. Am J Kidney Diseases, 2009 Jan 1;53(1):121-8.

7. Galan P, Kesse-Guyot E, Czernichow S, Briancon S, Blacher J, Hercberg S. Effects of B vitamins and omega 3 fatty acids on cardiovascular diseases: a randomised placebo controlled trial. BMJ, 2010; 341:c6273.

8. Armitage JM, Bowman L, Clarke RJ, Wallendszus K, Bulbulia R, Rahimi K, Haynes R, Parish S, Sleight P, Peto R, Collins R. Effects of homocysteine-lowering with folic acid plus vitamin b12 vs placebo on mortality and major morbidity in myocardial infarction survivors: a randomized trial. JAMA, 2010; 303:2486–2494.

9. van Wijngaarden JP, Swart KM, Enneman AW, Dhonukshe-Rutten RA, van Dijk SC, Ham AC, Brouwer-Brolsma EM, van der Zwaluw NL, Sohl E, van Meurs JB, Zillikens MC. Effect of daily vitamin B-12 and folic acid supplementation on fracture incidence in elderly individuals with an elevated plasma homocysteine concentration: BPROOF, a randomized controlled trial. Am J Clin Nutr, 2014; 100: 1578-86.

10. Bonaa KH, Njolstad I, Ueland PM, Schirmer H, Tverdal A, Steigen T, Wang H, Nordrehaug JE, Arnesen E, Rasmussen K. Homocysteine lowering and cardiovascular events after acute myocardial infarction. N Engl J Med, 2006; 354:1578–1588.

11. Ebbing M, Bleie O, Ueland PM, Nordrehaug JE, Nilsen DW, Vollset SE, Refsum H, Pedersen EK, Nygard O. Mortality and cardiovascular events in patients treated with homocysteine-lowering B vitamins after coronary angiography: a randomized controlled trial. JAMA, 2008; 300:795–804.

12. Huo Y, Li J, Qin X, et al. Efficacy of Folic Acid Therapy in Primary Prevention of Stroke Among Adults With Hypertension in China: The CSPPT Randomized Clinical Trial. JAMA, 2015; 313:1325–1335.

# Search strategies

Medline (Ovid MEDLINE® Epub Ahead of Print, In-Process & Other Non-Indexed Citations, Ovid MEDLINE® Daily and Ovid MEDLINE®) 1946 to present (July 2023)

1 Folic Acid/bl [Blood]

2 ((serum or plasma or status or concentration) adj5 folate).mp.

3 1 or 2

4 randomized controlled trial.pt.

5 controlled clinical trial.pt.

6 randomized.ab.

7 placebo.ab.

8 clinical trials as topic.sh.

9 randomly.ab.

10 trial.ti.

11 exp case control studies/

12 Case control.tw.

13 cross sectional.tw.

14 Cross-sectional studies/

15 health surveys/ or exp population surveillance/

16 exp Registries/

17 ((population or public health or health or national) adj3 (surveillance or survey or prevalence)).mp.

18 (registry or registeries).mp.

19 4 or 5 or 6 or 7 or 8 or 9 or 10 or 11 or 12 or 13 or 14 or 15 or 16 or 17 or 18

20 exp animals/ not humans.sh.

21 19 not 20

22 (exp child/ or exp infant/) not (exp adult/ or adolescent/)

23 21 not 22

24 3 and 23

25 limit 24 to yr="1990 - 2021"

26 Folic Acid/bl [Blood]

27 ((serum or plasma or status or concentration) adj5 folate).mp.

28 26 or 27

29 randomized controlled trial.pt.

30 controlled clinical trial.pt.

31 randomized.ab.

32 placebo.ab.

33 clinical trials as topic.sh.

34 randomly.ab.

35 trial.ti.

36 exp case control studies/

37 Case control.tw.

38 cross sectional.tw.

39 Cross-sectional studies/

40 health surveys/ or exp population surveillance/

41 exp Registries/

42 ((population or public health or health or national) adj3 (surveillance or survey or prevalence)).mp.

43 (registry or registeries).mp.

44 29 or 30 or 31 or 32 or 33 or 34 or 35 or 36 or 37 or 38 or 39 or 40 or 41 or 42 or 43

45 exp animals/ not humans.sh.

46 44 not 45

47 (exp child/ or exp infant/) not (exp adult/ or adolescent/)

48 46 not 47

49 28 and 48

Embase 1974 to present (July 2023)

1 folic acid blood level/

2 ((serum or plasma or status or concentration) adj5 folate).ti,ab.

3 1 or 2

4 randomized controlled trial/

5 single blind procedure/ or double blind procedure/

6 crossover procedure/

7 random*.tw.

8 (((singl* or doubl*) adj (blind* or mask*)) or crossover or cross over or factorial* or latin square or assign* or allocat* or volunteer*).ti,ab.

9 trial.ti.

10 Case control study.tw.

11 (Case control adj (study or studies)).tw.

12 (cross sectional adj (study or studies)).tw.

13 health survey/

14 Register/

15 ((population or public health or health or national) adj3 (surveillance or survey or prevalence?)).mp.

16 (registry or registries).mp.

17 4 or 5 or 6 or 7 or 8 or 9 or 10 or 11 or 12 or 13 or 14 or 15 or 16

18 3 and 17

19 (exp animals/ or nonhuman/) not human/

20 18 not 19

21 exp child/ not (exp adult/ or exp adolescent/)

22 20 not 21

Global Health – 1973 to present (July 2023)

1 Folic Acid.ti,ab.

2 ((serum or plasma or status or concentration) adj5 folate).ti,ab.

3 1 or 2

4 randomized controlled trials/

5 clinical trials/

6 randomized.ti.

7 placebo.ti.

8 randomly.ti.

9 trial.ti.

10 Case control study.tw.

11 (cross sectional adj (study or studies)).tw.

12 disease surveys/ or epidemiological surveys/

13 ((population or public health or health or national) adj3 (surveillance or survey or prevalence?)).mp.

14 (registry or registries).mp.

15 (Case control adj (study or studies)).tw.

16 4 or 5 or 6 or 7 or 8 or 9 or 10 or 11 or 12 or 13 or 14 or 15

17 3 and 16

18 (exp infants/ or exp children/) not (exp adults/ or adolescents/)

19 17 not 18
